# The postsynaptic scaffolding protein SAPAP3 shapes mitochondrial activity: the case of Huntington’s disease

**DOI:** 10.1038/s41419-026-09156-4

**Published:** 2026-08-07

**Authors:** Patrícia Coelho, Ildete Luísa Ferreira, Ana Sofia Lourenço, Daniela Marinho, Sandra Isabel Anjo, Zongwei Fang, Lígia Fão, Sandra I. Mota, Philippe J. Mas, Mário Carvalho, Rui Jorge Nobre, Carina Henriques, Joana Fraga, Dongqing Wang, Sandra Macedo Ribeiro, Luís Pereira de Almeida, Patrícia Monteiro, Isaura Simões, Darren J. Hart, Bruno Manadas, João Peça, Pedro Castanheira, A. Cristina Rego

**Affiliations:** 1 CNC-UC—Center for Neuroscience and Cell Biology, University of Coimbra, Coimbra, Portugal; 2 III-UC—Institute of Interdisciplinary Research, University of Coimbra, Coimbra, Portugal; 3 Biocant—Biotechnology Innovation Center, Cantanhede, Portugal; 4 FMUC—Faculty of Medicine, University of Coimbra, Coimbra, Portugal; 5 EMBL—European Molecular Biology Laboratory, Grenoble, France; 6 ISBG—Integrated Structural Biology Grenoble, CNRS, CEA, Université Grenoble Alpes, EMBL, Grenoble, France; 7 Department of Neuroscience, University of Copenhagen, Copenhagen, Denmark; 8 ViraVector—Viral Vectors for Gene Transfer Core Facility, University of Coimbra, Coimbra, Portugal; 9 i3S—Instituto de Investigação e Inovação em Saúde Portugal, Universidade do Porto, Porto, Portugal; 10 McGovern Institute for Brain Research, Department of Brain and Cognitive Sciences, Massachusetts Institute of Technology, Cambridge, MA USA; 11 Faculty of Pharmacy, University of Coimbra, Coimbra, Portugal; 12 Life and Health Sciences Research Institute (ICVS), School of Medicine, University of Minho, Braga, Portugal; 13 Department of Biological Sciences, Old Dominion University, Norfolk, VA USA; 14 Institut de Biologie Structurale, CEA, CNRS, Université Grenoble Alpes, Grenoble, France; 15 Department of Life Sciences, Faculty of Science and Technology, University of Coimbra, Coimbra, Portugal; 16 Present Address: Immunethep SA, Biocant Park, Nucleo 4, Cantanhede, Portugal

**Keywords:** Pathogenesis, Huntington's disease, Huntington's disease, Mitochondria

## Abstract

Postsynaptic scaffolding protein SAP90/PSD95-associated protein 3 (SAPAP3) modulates cortico-striatal signalling and regulates the maintenance of synaptic structure. Notably, SAPAP3 defects have been reported in several human psychiatric disorders that share pathophysiological features with Huntington’s disease (HD), a neurodegenerative disorder characterized by the expression of mutant huntingtin (mHTT) and marked dysfunction of cortico-striatal synapses and mitochondria. However, the role of SAPAP3 in mitochondrial function and HD pathophysiology remains unexplored. SAPAP3 was extracted from striatal synaptoneurosomes and analyzed by SWATH-MS proteomics to identify potential interactors, revealing SAPAP3 association with several mitochondrial proteins, particularly Mic60. These data were further complemented with proximity ligation assay (PLA) and immunoprecipitation experiments, supporting a direct or indirect interaction between SAPAP3 and Mic60. SAPAP3 knockout (SAPAP3-KO) mouse striatum exhibited reduced Mic60 protein levels, decreased mitochondrial respiratory chain activity and Ca^2+^ uptake, as well as increased mitochondrial roundness and circularity, accompanied by decreased perimeter and area, consistent with mitochondrial fragmentation. Analysis of SAPAP3 protein levels in *postmortem* caudate tissue from HD patients, striatal tissue and isolated mitochondria from YAC128 mice, as well as mitochondria in primary striatal YAC128 neurons and ST*Hdh* Q111 cells, showed consistently reduced SAPAP3 levels. HD cells and WT cells subjected to SAPAP3 silencing showed alterations in mitochondrial morphology, movement and function. In contrast, HD striatal cells transfected with ovSAPAP3 constructs to restore SAPAP3 levels showed a rescue of mitochondrial morphology, motility, function and Ca^2+^ handling, together with decreased levels of reactive oxygen species (ROS). These findings uncover a novel role for postsynaptic scaffolding protein SAPAP3 in preserving mitochondrial health, particularly mitochondrial dynamics and function, under neuropathological conditions characterized by mitochondrial abnormalities such as HD.

**Figure:**
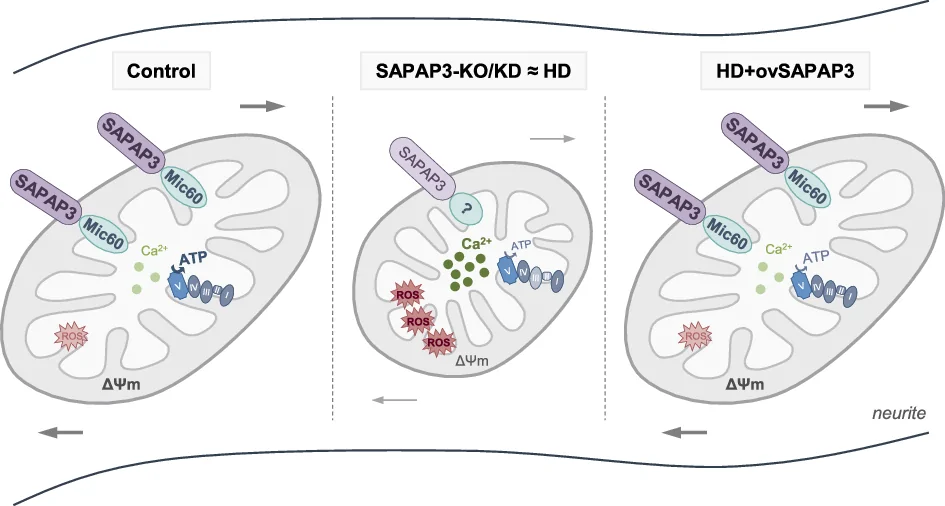

## Introduction

Postsynaptic scaffold proteins have been described to play an important role in regulating synaptic dysfunction in neurodegenerative conditions e.g. [1]. Among these proteins, SAP90/PSD95-associated proteins (SAPAPs) are scaffolding proteins located at the postsynaptic density (PSD), regulating synaptic transmission and homeostatic plasticity [2]. Within the SAPAP family, *SAPAP3* mRNA is highly expressed in the striatum, where it orchestrates the organization of key postsynaptic components, including AMPA and NMDA receptors at cortico-striatal synapses [3]. Disruption of SAPAP3 in knockout (KO) models results in aberrant synaptic activity, accompanied by the emergence of obsessive-compulsive disorder (OCD)-like behaviors [3].

Huntington’s disease (HD) is a polyglutamine-expansion neurodegenerative disorder that selectively affects striatal GABAergic medium spiny neurons that receive glutamatergic excitatory projections mainly from the cortex, perturbing the cortico-striatal circuitry that is essential for motor control (e.g. McColgan and Tabrizi, 2018). HD clinical manifestations encompass a spectrum of psychiatric disturbances, such as OCD and depression, motor impairments, cognitive decline and progressive dementia. Interestingly, OCD was observed in premanifest mutant huntingtin (mHTT) carriers [4, 5], while excessive self-grooming, an abnormal repetitive behavioral phenotype linked to OCD, was observed in the R6/2 HD mouse model e.g. [6], suggesting a potential link between SAPAP3 and HD pathogenesis.

Evidence of mitochondrial and synaptic dysfunction caused by intracellular aggregates of mHTT at the early stages of HD may be accounted for by a direct effect of mHTT on the organelle [7], transcriptional deregulation affecting mitochondrial biogenesis e.g. [8] and/or compromised mitochondrial integrity e.g. [9]. In this respect, mitochondrial morphology and motility were shown to depend on Mic60 [10, 11]. In our previous work, ultrastructural mitochondrial abnormalities, diminished oxidative capacity, and perturbed ATP synthesis were evident in skin-derived fibroblasts from presymptomatic HD carriers and in animal and neuronal models of HD [12, 13], along with changes in mitochondrial dynamics [13] and altered Ca^2+^ handling [14]. Moreover, altered activity of mitochondrial complexes II-IV and decreased mitochondrial membrane potential were observed in human HD-induced pluripotent stem cells and derived neural stem cells [15]. However, apart from Ca^2+^ dyshomeostasis, the identification of molecular mitochondrial targets linked to postsynaptic glutamatergic terminals with the potential to ameliorate or delay HD progression remains scarce. PSD proteins have long been studied due to their important role in postsynaptic signaling, regulating synaptic plasticity, while their relationship with mitochondrial function has also been highlighted [16, 17].

Apart from changes in cortico-striatal synapses and behavioral manifestations, mitochondrial deregulation has been described in HD, yet the role of SAPAP3 in this context is unknown. In this study, we identify interactions between the C-terminal domain of SAPAP3 and several mitochondrial proteins, including Mic60, within striatal synaptoneurosomes, shedding light on a potential link between SAPAP3 and mitochondrial dysfunction in HD pathogenesis. Data obtained in brain mitochondria from SAPAP3 mutant mice and HD cell models suggest that SAPAP3 constitutes a critical modulator of mitochondrial health, regulating mitochondrial bioenergetics and morphodynamics. These findings open new research directions for exploring SAPAP3 as a potential therapeutic molecular target for mitochondrial dysfunction in neurodegenerative diseases as HD.

## Methods

### Animals

SAPAP3 knockout (SAPAP3-KO) mice (B6.129-DLGAP3tm1Gfng/J strain) generated as described in [3] and matching wild-type (WT) mice at 3 months of age were obtained from the Life and Health Sciences Research Institute, School of Medicine, University of Minho and, upon quarantine, readily used for experiments. Colonies of hemizygous YAC128 [line HD53; generated as described in [18] with FVB/N background] at 3, 6 and 10-12 months of age and WT mice at 2, 3, 6 and 10-12 months of age were maintained at CNC-UC/FMUC animal house under a 12 h light/12 h dark cycle, in a controlled temperature and humidity environment with food and water provided *ad libitum*. Wistar rats were purchased from Charles River (Barcelona, Spain) and kept under the same environmental conditions until use. All studies were performed according to guidelines of EU directive 2010/63/EU and protocols approved by the FMUC (ORBEA_189_2018/11042018 and ORBEA_357_2024/17062024). All efforts were made to minimize stress and animal suffering and to reduce the number of animals used.

### Human samples

Human HD (Vonsattel grade II-III) and control *postmortem* tissue were obtained from the Neurobiobank München (Zentrum für Neuropathologie und Prionforschung, Germany [http://www.neurobiobank.org]). The ethical permit to use *postmortem* human samples was approved by Institutional Review Board from Faculty of Medicine, University of Coimbra, Coimbra, Portugal (reference no. CE-136/2015).

### ESPRIT construct generation

A cDNA encoding for full-length human SAPAP3 (RefSeq accession number NP_001073887) was synthesised with codons optimised for expression in *E. coli* (Genscript, Piscataway, NJ - USA) and then cloned between the AscI and NotI sites of pESPRIT002 [19] for use in the ESPRIT screening workflow, resulting in a library of SAPAP3 gene fragments spanning the reading frame [20, 21]. To avoid any non-intended interference with the ESPRIT screen, where immobilised metal affinity chromatography (IMAC) is used, the His rich region between amino acids 223 and 234 was deleted by PCR. The amplification of the whole plasmid construction was performed using Phusion polymerase (New England Biolabs) and phosphorylated primers flanking the His rich region in the outside direction (F: CAGTCTCGTCATGGTAAACGC; R: TGAGGTGTGCGGGCC - IDT - Integrated DNA Technologies, Coralville, IA - USA). The DNA amplification was confirmed by agarose gel electrophoresis, and the parent DNA was digested by DpnI (New England Biolabs) for 2 h followed by DNA recircularization using T4 DNA Ligase (New England Biolabs). The sequence of both constructs was confirmed by DNA sequencing. High-throughput screening of SAPAP3 for soluble domains by incremental truncation was performed [20, 21]; described in detail in supplementary information.

### Isolation of synaptoneurosomes

Synaptoneurosomes were prepared at 4 °C as previously described with minor modifications [22]. Briefly, striata from four adult female Wistar rats were dissected and homogenised using a loose-fitting pestle in purification buffer (10 mM HEPES-Tris pH 7.4, 0.32 M sucrose, 0.1 M EGTA), followed by homogenisation with a tight-fitting pestle. After centrifugation for 3 min at 800 × *g*, the supernatants were collected and filtered sequentially through nylon membranes with decreasing pore sizes: 150-, 50- and 8 μm, and the flow-through was centrifuged at 10,000 × *g* for 15 min for synaptoneurosomes sedimentation. The pellets were resuspended in purification buffer supplemented with inhibitors cocktails of protease (cOmplete, EDTA free, Roche Diagnostics) and phosphatase (PhosStop, Roche Diagnostics) and stored at -80 °C.

### SAPAP3 affinity purification assay

Frozen synaptoneurosomes were thawed on ice, centrifuged at 10,000 × *g* for 15 min at 4 °C and resuspended in lysis buffer (20 mM Tris-HCl, pH 7.4, 100 mM NaCl, 1% (v/v) Triton X-100, 0.5 mM DTT, 5 mM MgCl_2_, supplemented with protease and phosphatase inhibitors and kept on ice for 15 min. Lysed synaptoneurosomes were then centrifuged at 10,000 × *g* for 15 min at 4 °C and the supernatants collected and protein concentration measured using the Pierce BCA protein assay kit (Thermo Fisher Scientific).

SAPAP3_G^754^-I^968^ domain, biotinylated at the C-terminal biotin acceptor peptide tag (20 μg) was added to the magnetic beads of Streptavidin Mag Sepharose (Cytiva, Marlborough, MA, USA) according to the instructions of the supplier. The magnetic beads were then incubated with 180 μg of synaptoneurosomal extracts during 3 h, at 4 °C, followed by 3 wash steps. To monitor sample loss and to adjust retention times, samples were spiked with recombinant MBP-GFP - a fusion protein between maltose-binding protein (N-terminal) and green fluorescent protein (C-terminal domain), at 1.84 mg/mL before digestion [23]. Samples were denatured using Laemmli sample buffer at 95 °C for 15 min.

### SWATH-MS

Sequential Windowed data-independent Acquisition of the Total High-resolution Mass Spectra (SWATH-MS) data acquisition and analysis was performed as previously described [24] with minor modifications; detailed in supplementary information.

### Primary striatal and cortical neurons

Primary cultures of striatum and cortex were obtained from the offspring at embryonic day 16 (E16)-E17 of crosses between heterozygous male YAC128 mice and WT females as described in [25]. Brain areas were maintained in hibernate E medium (Thermo Fisher, Waltham, MA USA) and pooled together according to the respective genotype. Alternatively, one animal was used for one cell culture and genotyping (detailed in supplementary information) performed subsequently. Cells were seeded in poly-D-lysine (0.1 mg/mL)-coated plates or coverslips and cultured in a humidified incubator containing 95% air and 5% CO_2_ at 37 °C. To avoid glial proliferation, 10 μM 5-fluoro-2’-deoxyuridine (Sigma, St Louis, MO, USA) was added 72 h after plating. After 7 days in vitro (DIV) half of the medium was replaced with fresh supplemented Neurobasal medium (Thermo Fisher, Waltham, MA USA) and cells used at DIV12.

### Immortalized striatal cell lines

Striatal ST*Hdh* cells expressing WT Htt (Q7 cells; #CHDI-90000073) or mHtt (Q111 cells; #CHDI-90000071) [26] were obtained from Coriell Institute for Medical Research (Hadden Avenue Camden, USA). Cells were grown adherent at 33 °C in DMEM (Sigma, St Louis, MO, USA) supplemented with 10% (v/v) fetal bovine serum (Thermo Fisher, Waltham, MA USA), 4 mM glutamine, 100 U/mL penicillin plus 0.1 mg/mL streptomycin (GIBCO, Paisley, UK) and 400 μg/mL geneticin (GIBCO, Paisley, UK) in a humidified incubator chamber containing 95% air and 5% CO_2_.

### SAPAP3 plasmid constructs

For SAPAP3 silencing, a short-hairpin RNA (shRNA) targeting SAPAP3 was cloned downstream of the U6 promoter in an adeno-associated virus vector plasmid (shSAPAP3). The construct also included a GFP reporter gene driven by the cytomegalovirus (CMV) enhancer/promoter (Vigene Biosciences, Rockville, MD, USA). For SAPAP3 overexpression, the lentiviral vector backbone FUGW was used. To enhance neuronal expression, the ubiquitin promoter was replaced with a CMV promoter. GFP was fused to the amino terminus of SAPAP3 and inserted downstream of the CMV promoter, between the BamHI and EcoRI restriction sites, generating the ovSAPAP3 construct [3]. As a control, a plasmid containing a U6 promoter and a GFP reporter gene under the regulation of the CMV enhancer/promoter was used.

### Transfection

Cell lines were transfected 24 h after plating with 0.5 μg DNA (control, shSAPAP3 or ovSAPAP3, and MitoDsRed plasmids (Takara Bio, Kusatsu, Japan) per cm^2^ of plating area in Opti-MEM reduced serum medium (Thermo Fisher, Waltham, MA USA) using the Lipofectamine 3000 kit (Thermo Fisher, Waltham, MA USA), and medium changed 4 h after transfection. Primary cultures were transfected at DIV8 with 0.4 μg DNA (control, shSAPAP3 or ovSAPAP3, and MitoDsRed plasmids) per cm^2^ of plating area using the calcium phosphate co-precipitation transfection kit (Thermo Fisher, Waltham, MA USA). Both cells were cultured for additional 48 h post-transfection. The effect of transfection on cell viability was evaluated (detailed in supplementary information).

### Immunocytochemistry and image analysis

Cells were fixed with 4% (w/v) paraformaldehyde for 20 min, permeabilized in 0.1% (v/v) Triton X-100 in PBS for 2 min and blocked for 1 h at room temperature in 3% (w/v) BSA in PBS. SAPAP3 (rabbit, 1:500 (provided by Guoping Feng lab); or mouse, 1:2 (NeuroMab, Davis, CA, USA), Mic60 (1:150) (Abcam, Cambridge, UK), MCU (1:150) (Cell Signaling, Danvers, MA, USA) and Drp1 (1:300 (BD Biosciences), TOM20 (rabbit, 1:250) (Proteintech, Planegg, Germany), LAMP1 (rat, 1:100) (DSHB, Iowa City, Iowa, USA) and IP3R3 (rabbit, 1:250) (Cell Signaling, Danvers, MA, USA) primary antibodies were prepared in 3% (w/v) BSA in PBS and incubated overnight at 4 °C, and secondary antibodies, Alexa Fluor-594 anti-rabbit, Alexa Fluor-568 anti-mouse, Alexa Fluor-647 anti-rabbit, Alexa Fluor-647 anti-mouse (Thermo Fisher, Waltham, MA USA) were incubated for 1 h at room temperature. Nuclei were labeled with 4 μg/mL Hoechst 33342 for 20 min and slides mounted using Mowiol 40-88. Confocal images were obtained as stacks along the z axis (z-stacks) using a plan-apochromat/1.4NA 63x lens on a Carl Zeiss LSM 710 microscope. FIJI was used for image analysis. Proximal neurites were defined as those located within 50 μm of the soma ( < 50 μm), whereas distal neurites were analyzed at distances greater than 50 μm from the soma ( > 50 μm) as in [27, 28]. For SAPAP3- and Mic60 total cellular quantification of protein levels, z-stacks images were transformed into a z-projection corresponding to the maximum intensity and normalised for the background (rolling ball radius of 15). To quantify SAPAP3-, Mic60- and MCU protein levels in mitochondrial sites, a screen along the imaged z-stack was performed and single plains were extracted and all signals were normalised for the background (rolling ball radius of 5 for mitochondria and Mic60, 15 for SAPAP3 and 20 for MCU). GFP signal, an indicator of SAPAP3-transfected cells, was used to draw regions of interest (ROIs) which defined cellular boundaries; total cellular protein levels were calculated by the ratio of the mean fluorescent intensity (integrated density) to the designed ROI (corresponding to the cell area). Mitochondrial ROIs were defined by using the FindFoci function and a threshold was applied to optimally resolve individual mitochondria, followed by the Analyze Particles function to trace mitochondrial outlines. Then, relative protein levels were calculated by the ratio of the integrated density to the selected mitochondrial ROIs. Mitochondrial morphology analysis was achieved using macros designed by Doctor Jorge Valero (CNC-UC, presently at Achucarro – Basque Center for Neuroscience, Spain). For correlation studies, images were normalised for the background (rolling ball radius of 5 for MitoDsRed and Mic60; 15 for SAPAP3, LAMP1 and TOM20; 30 for IP3R3) and in the cellular ROIs, JACoP FIJI-plugin [29] was applied and the Manders’ colocalization coefficients were calculated (after automatic thresholding to exclude background signal) to assess colocalization levels between SAPAP3 and MitoDsRed or TOM20 (mitochondria), LAMP1 (lysosomes), IP3R3 (endoplasmic reticulum), PSD95 and Mic60 signals, leading to a pixel-by-pixel quantification [30]. Specifically, Manders’ M1 coefficient was used to quantify the fraction of SAPAP3 signal overlapping the second channel. For colocalization analyses, images were background-normalized using a rolling ball radius of 5 for MitoDsRed and Mic60, 15 for SAPAP3, LAMP1, and TOM20, and 30 for IP3R3. Analyses were performed within cellular ROIs using the JACoP FIJI plugin [29]. After automatic thresholding to exclude background signal, Manders’ colocalization coefficients were calculated to assess signal overlap between SAPAP3 and MitoDsRed or TOM20 (mitochondria), LAMP1 (lysosomes), IP3R3 (endoplasmic reticulum), PSD95, and Mic60 [30]. Specifically, the Manders’ M1 coefficient was calculated as the summed intensity of SAPAP3 pixels overlapping with the second channel divided by the total SAPAP3 intensity, thereby quantifying the fraction of SAPAP3 signal colocalizing with the corresponding marker.

### Proximity ligation assay

Proximity ligation assay (PLA) was performed by Duolink in situ Red Starter Kit (DUO92101, Sigma, St Louis, MO, USA) in cells previously fixed with 4% (w/v) paraformaldehyde for 20 min and permeabilized in 0.1% (v/v) Triton X-100 in PBS for 2 min. Primary antibodies (rabbit SAPAP3 antibody, 1:500; mouse Mic60 antibody, 1:150) were used. Confocal images were obtained as z-stacks using a plan-apochromat/1.4NA 63x lens on Carl Zeiss LSM 710 microscope and then transformed into a z-projection normalised for the background (rolling ball radius of 5 for Mic60 and 15 for SAPAP3) using the FIJI software. The number of PLA dots *per* cell was calculated by the ratio of the number of red dots to the area of the cellular ROI (GFP signal).

### Mitochondrial movement analysis

Single-cell analysis was performed for 15 min at 33 °C using a Carl Zeiss cell observer spinning disk microscope with a LCI plan-neofluar/1.3NA 63x lens. Time-lapse images were processed using FIJI software; a bleach correction plugin was applied to correct cellular fluorescence intensity, followed by a StackReg plugin to correct Z movement throughout the experiment. Next, a KymoAnalyser plugin was applied to assess multiple mitochondrial parameters, as described in [31].

### Mitochondrial isolation

SAPAP3-KO, YAC128 and respective WT mice were sacrificed by cervical dislocation and decapitation. Mitochondria from cortex, striatum, hippocampus and cerebellum were isolated using discontinuous Percoll (GE Healthcare, Chicago, IL, USA) density gradient centrifugation, according to [32]. The protein content was evaluated by the Bio-Rad Bradford protein assay. Isolated mitochondria were kept on ice for 2 h for further functional analysis with additional samples for western blot analysis (detailed in supplementary information) being frozen at -80 °C.

### Oxygen consumption rate measurements

Oxygen consumption rate (OCR) was measured by using the Seahorse XF24 flux analyzer (Agilent) as previously described [32] in both freshly isolated mitochondria and cell lines. The sequential determination of mitochondrial respiration was performed by using different modulators and inhibitors as indicated in figure legends. Results are expressed in picomoles of O_2_ per minute per microgram of protein.

### Ca^2+^ handling in isolated mitochondria

Mitochondrial Ca^2+^ uptake was measured fluorometrically in the presence of the Ca^2+^-sensitive fluorescent dye Calcium Green 5 N (150 nM; Thermo Fisher, Waltham, MA USA), using excitation and emission wavelengths of 506 nm and 532 nm, respectively [32]. Data were expressed as traces reflecting the rate of decrease of Calcium Green-5N fluorescence and thus, the capacity of mitochondria to take up Ca^2+^. To better understand the differences in Ca^2+^ handling capacity in response to a 2^nd^ calcium pulse, the area under the curve, which represents the extra-mitochondrial calcium, was also calculated and plotted.

### ΔΨm and mitochondrial Ca^2+^ levels in striatal cells and neurons

Striatal cells were loaded with TMRM^+^ (300 nM, quenched mode, Molecular Probes, Invitrogen) under quenched mode conditions, for 30 min at 33 °C) or with Rhod-2/AM probe (1 μM, Molecular Probes, Invitrogen) for 40 min at 33 °C, to assess ΔΨm and mitochondrial Ca^2+^ (mitoCa^2+^), respectively, in Na^+^ medium. To directly assess the role of mitochondria, glycolysis was inhibited, replacing glucose by 2-deoxy-D-glucose (2 mM) and by adding pyruvate (10 mM) in Na^+^ medium (pyruvate-based medium). Fluorescence was measured using a Spectrofluorometer Gemini EM microplate reader. To assess ΔΨm, basal fluorescence was recorded (excitation at 550 nm; emission at 573 nm) and then oligomycin (2 μg/mL) plus FCCP (2 μM) were added to completely depolarize the mitochondria under conditions of ATP synthase inhibition. For mitoCa^2+^ evaluation, fluorescence levels of Ca^2+^-bound Rhod2 were measured for 15 min (excitation at 552 nm; emission at 581 nm).

Striatal neurons were loaded with 10 nM TMRM^+^ (unquenched mode) in Na^+^ medium for 30 min at 37 °C. TMRM+ fluorescence was monitored at 37 °C using a ZEISS spinning disk confocal microscope with a 63x objective lens (excitation at 543 nm; emission at 458 nm). After a baseline, neurons were exposed to FCCP (2 μM) to completely depolarize mitochondria. Fluorescence intensity at each time point was analyzed in Fiji software using the time series analyser plugin (v 3.0).

### Reactive oxygen species (ROS) levels measurement

Cellular H_2_O_2_ levels were measured by using 10 μM AmplexRed (Thermo Fisher, Waltham, MA USA) plus 0.5 units/mL horseradish peroxidase (Sigma, St Louis, MO, USA) for 12 min at 33 °C (571 nm excitation; 585 nm emission) [32], using a microplate reader Spectrofluorometer Gemini EM (Molecular Devices).

Superoxide radical (O_2_^• –^) levels were measured following a 40 min loading with 2 µM MitoSOX Red (Thermo Fisher, Waltham, MA USA) at 33 °C. Cells were exposed to the mitochondrial complex III inhibitor myxothiazol (4 μM; Sigma, St Louis, MO, USA) and then fluorescence was recorded at 510 nm excitation and 580 nm emission using an Axio Observer Z1 system with an EC plan-neofluar/1.3NA 40x lens. Fluorescence intensities were calculated using FIJI software.

### Transmission electron microscopy (TEM)

For transmission electron microscopic (TEM) ultrastructural analyses striatum was dissected out from 3-month-old WT and SAPAP3-KO mice brain and processed as detailed in supplementary information.

### Statistical analysis

SAPAP3_G^754^-I^968^ domain potential interactors were identified by applying a Student’s t-test with a 5% false discovery rate (FDR) correction using the Perseus 1.6.14.0 Software (Max Planck Institute of Biochemistry). Gene ontology analysis was performed with BiNGO app (v. 3.0.4) in Cytoscape (v. 3.8.2) for the list of the 30 genes identified with a statistically significant difference between the two experimental conditions. Data were analysed using Excel and GraphPad Prism 8.0 software, and expressed as mean ± S.D. of the number of independent experiments indicated in figure legends. Specifically, striatal cell lines and neurons were performed using 3-9 independent experiments, considering 5-21 cells *per* condition in single cell analysis or in quadruplicates. In mouse experiments 3-9 animals *per* genotype were used. Four human *postmortem* brain samples *per* genotype were analysed. For TEM images analyses, 281-286 mitochondria *per* genotype were considered. No power analysis was performed. Investigators were blinded to the group allocation during the experiments, but not when assessing the outcome. No randomization of samples was performed. Statistical significance was assessed using the Mann-Whitney test, unpaired Student’s t-test, Kruskal-Wallis followed by uncorrected Dunn’s multiple comparison test, one-way ANOVA followed by uncorrected Fisher’s LSD multiple comparison test or two-way ANOVA followed by the Tukey’s multiple comparison test. Data met the assumptions of the statistical tests. Significance was defined as *p* < 0.05. Outliers were detected using ROUT method (Q = 1%).

## Results

### SAPAP3 interactome is enriched in mitochondrial proteins and involves Mic60

SAPAP3 is a 979 amino acid protein that is poorly understood at functional and structural levels. To characterize SAPAP3, putative domains expressed in *E. coli* (Fig. S1) were first identified by ESPRIT. In total, 27,642 clones were screened, and 19 promising clones selected after small-scale expression testing. Three of them, covering the C-terminal region (SAPAP3_G^754^-I^968^, SAPAP3_G^706^-S^967^ and SAPAP3_L^745^-Q^941^), yielded purifiable amounts of protein after large scale protein expression and purification, with the best results obtained for the SAPAP3_G^754^-I^968^ domain (Figs. S2 and S3a, b), which was selected for further experiments. Quality control analysis of the purified SAPAP_G^754^-I^968^ domain revealed a well-folded protein domain with high alpha-helical content (Fig. S3), compatible with the secondary structure prediction algorithms based on its amino acid sequence, as well as with the structural model of rat SAPAP1 C-terminal domain, which also displays a high content of alpha-helical secondary structure [33].

**Fig. 1:**
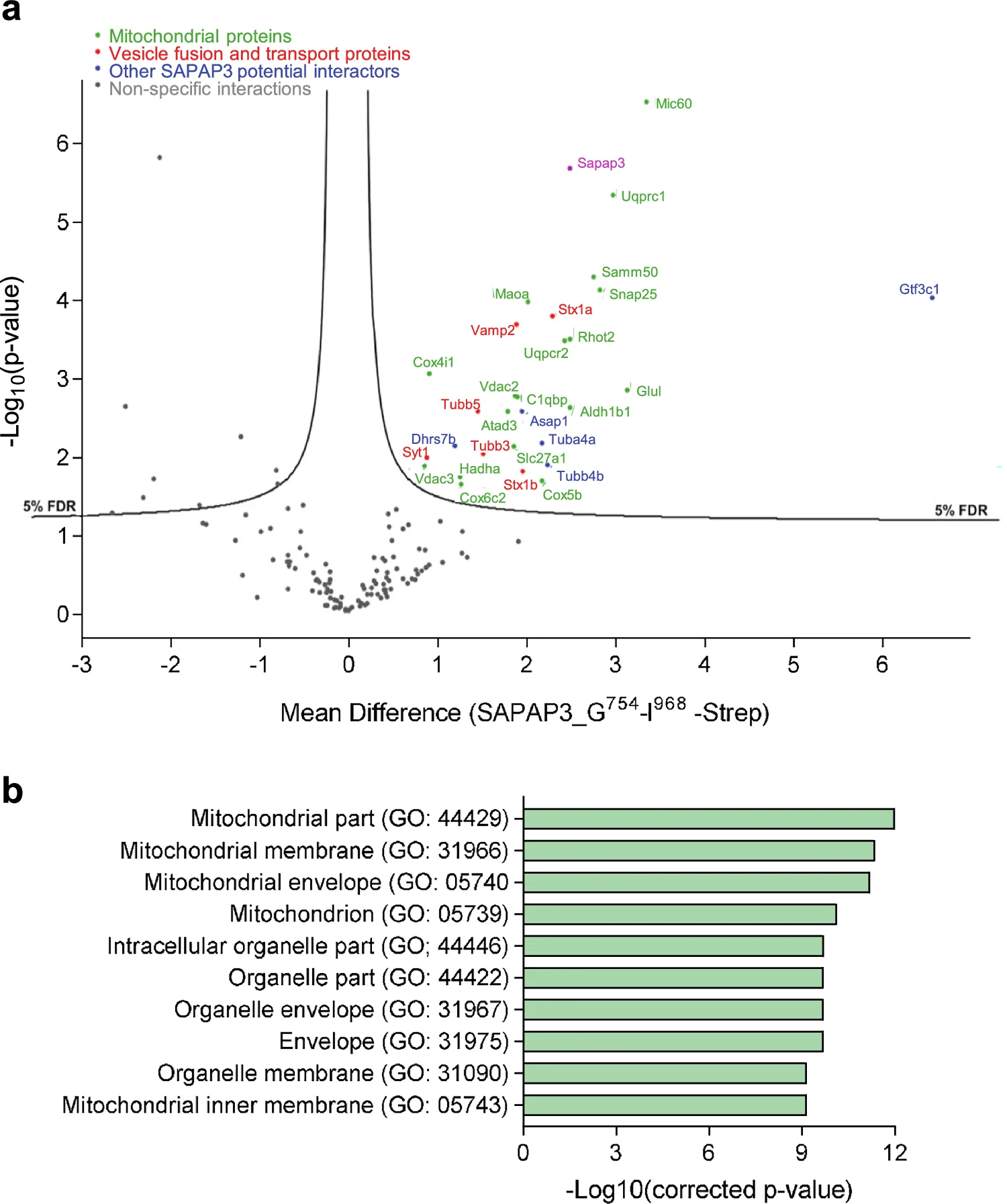
SAPAP3_G^754^-I^968^ domain interactome. Volcano plot with the results from the statistical analysis of all 148 synaptoneurosomal proteins quantified among the pull-down assay of the SAPAP3_G^754^-I^968^ domain versus the negative control (naked beads). Highlighted data points represent the 29 putative interactors of SAPAP3_G^754^-I^968^ domain considering an FDR of 5% using a Student’s t-test (**a**). Gene ontology (cellular localisation) analysis of the list of proteins identified as SAPAP3_G^754^-I^968^ domain putative interactors, using BiNGO app in Cytoscape (**b**).

To shed light onto the function of the C-terminal domain of SAPAP3, we investigated the interaction partners of the SAPAP3_G^754^-I^968^ domain. The purified proteins were analysed by SWATH-MS allowing the relative quantification of 148 proteins (Fig. 1a and Table S1), including the SAPAP3_G^754^-I^968^ domain (Fig. S2). From these, 29 proteins were shown to be enriched with statistical significance in the presence of the SAPAP3_G^754^-I^968^ domain (Fig. 1a; Table S1), corresponding to putative SAPAP3 interactors, namely mitochondrial- and vesicle function- and transport-related proteins (Fig. 1a, green and red dots, respectively). When the list of putative interactors was analysed for Gene Ontology by BiNGO app in Cytoscape, weighted through Cellular Component, the most statistically significant group of proteins was related to mitochondria (Fig. 1b). Consistently, apart from its high colocalization with the postsynaptic protein PSD95 in striatal mouse proximal and distal neurites, as analysed by Manders’ coefficient, SAPAP3 also shows high colocalization with the mitochondrial marker TOM20, when compared to lysosomal (LAMP1) or endoplasmic reticulum (IP3R3) markers (Fig. S4a, b).

Mic60, a crucial component of the mitochondrial contact site and cristae reorganizing system (MICOS) at the mitochondrial inner membrane, e.g. [10], was shown to be the highest-confidence mitochondrial protein to act as a specific interactor of SAPAP3 G^754^-I^968^ domain (Table S1). To assess the association of SAPAP3 with mitochondria, SAPAP3 levels were modulated by SAPAP3 silencing (shSAPAP3) and overexpression (ovSAPAP3), followed by characterization of its mitochondrial interactor, Mic60. shSAPAP3 and ovSAPAP3 (main sequences depicted in Fig. S5a) were confirmed by fluorescence microscopy in both mouse striatal WT/Q7 cell line (Fig. S5b, c) and WT striatal primary neurons (Fig. S5d–g). Results show that shSAPAP3 reduced SAPAP3 levels whereas ovSAPAP3 increased SAPAP3 levels in Q7 cells (Fig. S5b, c) and in soma and neurites of striatal neurons (Fig. S5d–g). Importantly, neither SAPAP3 silencing nor overexpression significantly affected cell viability (Fig. S5h–j).

**Fig. 2:**
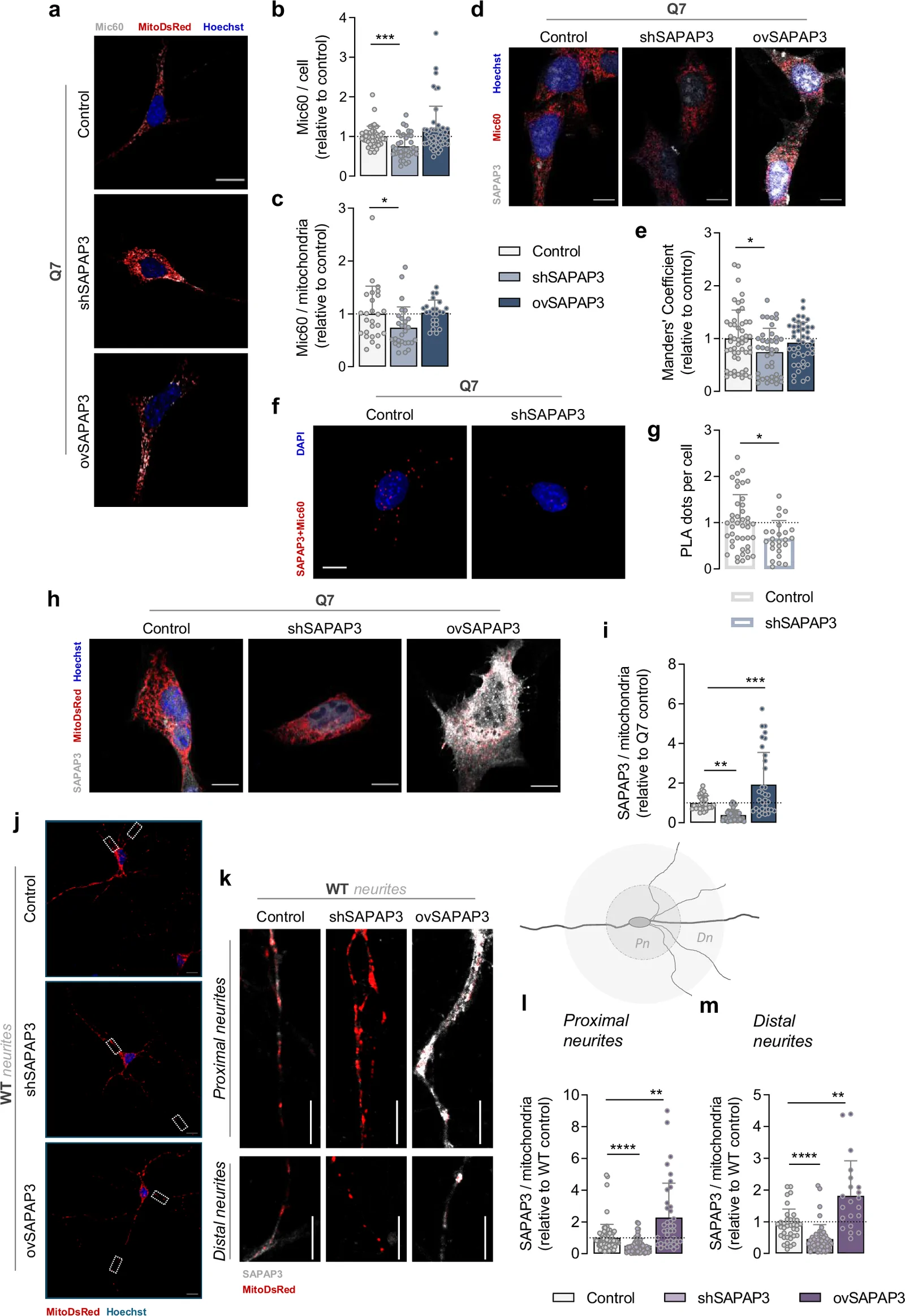
Mic60 interaction with SAPAP3 and mitochondrial SAPAP3 levels in striatal cells. Striatal cells (Q7 cells and WT neurons) were transfected with either GFP-control, -shSAPAP3 or -ovSAPAP3 plasmids. Representative images of Mic60 (grey) in Q7 cells labelled with MitoDsRed (red) (**a**) and quantification of the cellular (**b**) and mitochondrial (**c**) levels. Representative images of colocalization between Mic60 (red) and SAPAP3 (grey) (**d**) and quantification using Manders’ coefficient (**e**). Representative images of physical interaction between SAPAP3 and Mic60 (red) by in situ PLA (**f**) and quantification of number of PLA puncta (**g**). Representative images of SAPAP3 levels (grey) within mitochondria (red, labelled by MitoDsRed) in Q7 cells (**h**) and respective quantification (**i**). Representative images of SAPAP3 levels (grey) within mitochondria (red, labelled by MitoDsRed) in WT neurons (**k**) and quantification at proximal (**l**) and distal (**m**) neurites. Pictogram: proximal neurites (Pn) were defined as those located within 50 μm of the soma ( < 50 μm), whereas distal neurites (Dn) were analyzed at distances greater than 50 μm from the soma ( > 50 μm). Images in **k** are derived from the corresponding low-magnification images in **j** and Fig. S5k (complete set of images showing GFP expression). Nuclei are presented in blue (Hoechst staining). Scale bar = 10 μm. Data are the mean ± SD of 3 independent experiments, considering 5-15 cells per condition. Statistical analysis: *p < 0.05, **p < 0.01, ***p < 0.001 and ****p < 0.0001 by Kruskal-Wallis’s followed by uncorrected Dunn’s test, one-way ANOVA followed by uncorrected Fisher’s LSD test, or Mann Whitney test.

Interestingly, we found a decrease in total Mic60 protein levels following SAPAP3 silencing, when compared to the control, whereas SAPAP3 overexpression did not significantly affect Mic60 protein levels in a striatal cell line (Fig. 2a, b). A similar decrease was found in mitochondrial Mic60 levels in shSAPAP3-transfected cells (Fig. 2a, c). Moreover, in cells transfected with shSAPAP3, colocalization between SAPAP3 and Mic60 was reduced as analysed by Manders’ coefficient, when compared to the control (Fig. 2d, e). Of note, this decrease in Mic60 levels is unrelated to a reduction in mitochondrial area, as quantification was normalized to the whole mitochondrial region. Additionally, a direct or indirect interaction between SAPAP3 and Mic60 was significantly decreased following SAPAP3 silencing, as evaluated by the PLA assay (Fig. 2f, g). As such, silencing SAPAP3 decreased mitochondrial SAPAP3 levels, whereas ovSAPAP3 significantly increased SAPAP3 within mitochondria in striatal cell lines (Fig. 2h, i) and proximal and distal neurites from striatal neurons (Fig. 2j–m). Complementary validation of SAPAP3-Mic60 association was assessed after endogenous Mic60 immunoprecipitation (IP) experiments in 2- and 6-month-old mouse striatum (Fig. S4c). Although SAPAP3 was detected in the negative control (IP-), corresponding to non-specific binding to the IP matrix and/or the antibodies, the amount of SAPAP3 (both α and β isoforms) co-purified with Mic60 (IP Mic60) was markedly higher. Our data reveal an enrichment of SAPAP3 interactome in mitochondrial proteins, being Mic60 a relevant direct or indirect interactor. These data point to a potential novel role of SAPAP3 in interacting with mitochondrial proteins.

### Mitochondrial deregulation in brain regions of SAPAP3 knockout mice

**Fig. 3:**
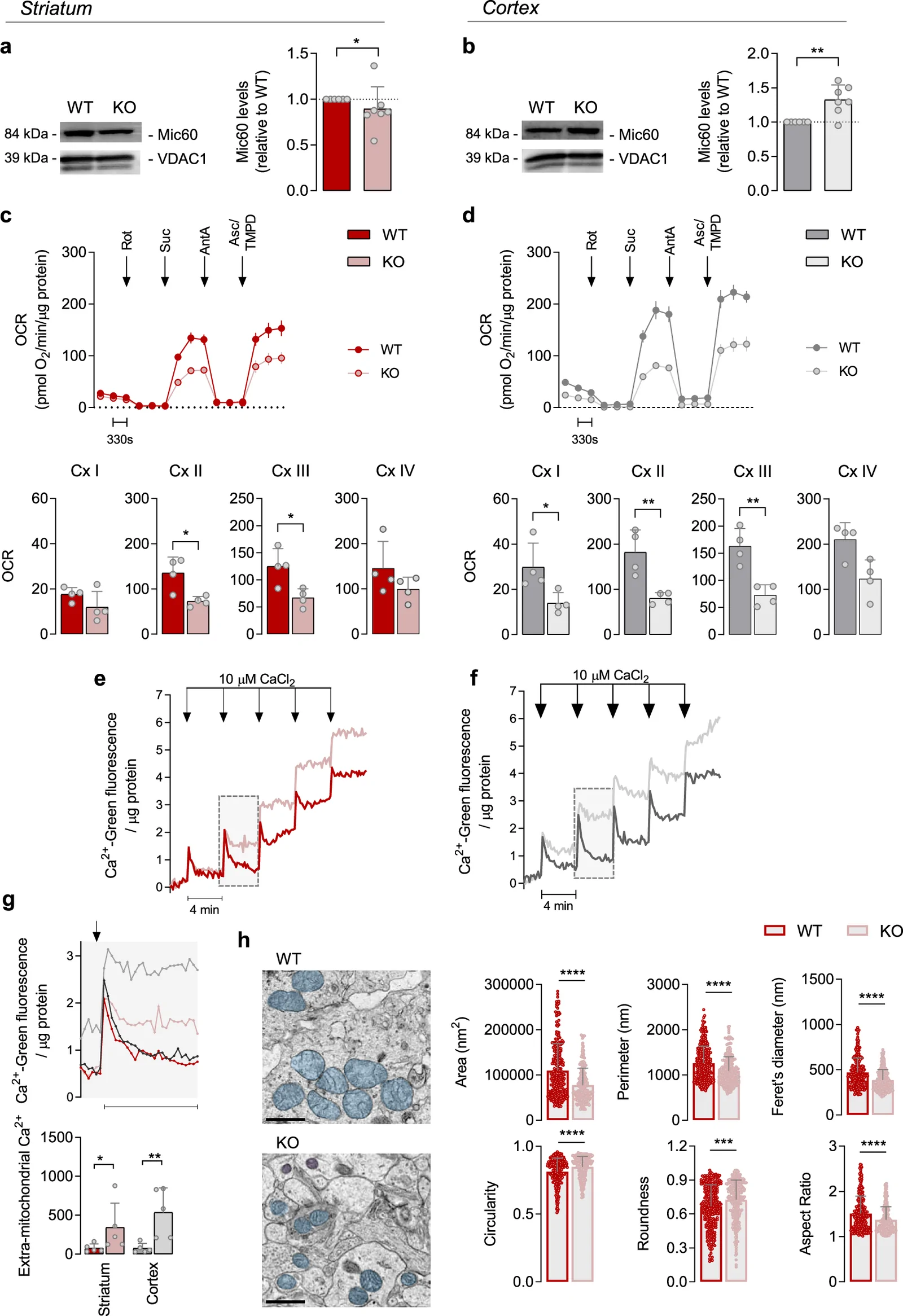
Mic60 levels, activity of respiratory chain complexes, mitochondrial Ca^2+^-handling and morphology in SAPAP3-KO mouse striatal and cortical mitochondria. Levels of Mic60 in isolated mitochondria from striatum (**a**) and cortex (**b**) were analysed by western blotting. VDAC1 was used as loading control. The activity of mitochondrial respiratory chain complexes Cx I-IV was evaluated in 3-month-old SAPAP3-KO and WT mice isolated striatal (**c**) and cortical (**d**) mitochondria following sequential addition of rotenone (2 µM), succinate (10 mM), antimycin A (4 µM; Sigma, St Louis, MO, USA) and ascorbate (10 mM) plus 100 µM N,N,N’,N’-tetramethyl-p-phenylenediamine dihydrochloride (TMPD, Sigma, St Louis, MO, USA). Striatal (**e**) and cortical (**f**) mitochondria were also analysed for their capacity to handle Ca^2+^ using a calcium sensitive probe. Five pulses of 10 μM Ca^2+^ (arrows) were applied to raise intramitochondrial Ca^2+^concentrations until the Ca^2+^ retention capacity was reached, as shown by the upward deflection of the trace. The decrease in Ca^2+^ handling capacity after the second Ca^2+^ pulse observed for striatal and cortical mitochondria is detailed in the light grey window (**g** above) and results presented as the increase in the extra-mitochondrial Ca^2+^ (**g** below). Data are the mean ± S.D. of independent mitochondrial preparations derived from 4-7 mice. Transmission electron micrographs were obtained from 4-month-old SAPAP3-KO and WT mouse striatum and mitochondrial morphology characterized by the analysis of area, perimeter, Feret’s diameter, circularity, roundness and aspect ratio, as evaluated by Fiji-imageJ software (scale bar: 1000 nm) (**h**). Data are the mean ± SD of 281-286 mitochondria *per* genotype. Statistical analysis: *p < 0.05, **p < 0.01, ***p < 0.001 and ****p < 0.0001 by Mann Whitney test.

To test the role of SAPAP3 in mitochondria, the activity of isolated mitochondria derived from SAPAP3-KO mouse brain was analysed. For this purpose, striatal, cortical, hippocampal and cerebellar-enriched mitochondrial fractions were isolated from 3-month-old SAPAP3-KO and WT mice. SAPAP3 was shown to be associated with ex vivo purified WT-derived mitochondria and absent in mitochondria derived from SAPAP3-KO mice, as expected (Figs. S6a and S12). SAPAP3 levels were lower in cerebellar mitochondria when compared to cortical mitochondria in WT mice (Fig. S6bi), whereas no differences were observed on VDAC1 levels, evaluated as a loading control, in SAPAP3-KO as compared to WT mice for all brain areas tested (Fig. S6bii). In agreement with SAPAP3-Mic60 association in striatum (Figs. 1, 2 and S4c), Mic60 was shown to be decreased in striatal mitochondria from SAPAP3-KO mice (Fig. 3a). Conversely, SAPAP3-KO cortical mitochondria exhibited an increase in Mic60 protein levels (Fig. 3b).

To address the impact of the absence of SAPAP3 on mitochondrial function, the respiratory activity of mitochondria isolated from SAPAP3-KO and WT mice was evaluated by determining the electron flow through the mitochondrial respiratory chain (MRC), which is indicative of the activity of complexes (Cx) I-IV (Fig. 3c, d). SAPAP3-KO striatal and cortical mitochondria presented a significant decrease in Cx activities, namely in Cx II and III in striatum, and Cx I, II and III in cortex, when compared with WT mitochondria from the same brain regions (Fig. 3c, d). No differences were found in mitochondria from hippocampal or cerebellar regions (Fig. S6c, d). Interestingly, protein levels of the 70 kDa-Cx II subunit were shown to be increased in striatal- (Figs. S7a and S12) and cortical-derived mitochondria (Figs. S7b and S12). To further analyse the protein levels of mitochondrial respiratory chain complexes, striatal and cortical brain samples of WT and SAPAP3-KO mice were labelled with total OXPHOS antibody cocktail. Data show increased levels in Cx I - NDUFB8, Cx II - SDHB and Cx IV - MTCO1 (normalized to VDAC1) in SAPAP3-KO striatum but preserved levels in the cortex (Figs. S8 and S13). These data suggest that increased levels of selective Cx subunits occur through an apparent compensatory process.

Because reduced mitochondrial Ca^2+^ handling may result from modified mitochondrial function, Ca^2+^ uptake was monitored in mitochondrial fractions from the same brain regions of WT and SAPAP3-KO mice (Figs. 3e–g and S6e, f). Ca^2+^ was rapidly taken up by isolated mitochondria after a single Ca^2+^ pulse, as evaluated by measuring the uptake kinetics of extra-mitochondrial Ca^2+^. A significant decrease in Ca^2+^ uptake was observed in striatal (Fig. 3e, g) and cortical (Fig. 3f, g) mitochondria obtained from SAPAP3-KO mice after the second pulse of Ca^2+^ (grey rectangle), suggesting abrogated Ca^2+^ handling capacity. Consistent with the activity of mitochondrial complexes (Fig. S6c, d), neither hippocampal (Fig. S6e) nor cerebellar (Fig. S6f) mitochondria Ca^2+^ uptake was affected by the loss of SAPAP3.

An abnormal ability to retain Ca^2+^ may be accounted for by dysregulated mitochondrial Ca^2+^ levels. Mitochondrial Ca^2+^ uptake occurs through the mitochondrial Ca^2+^ uniporter (MCU) complex. Interestingly, a significant increase in MCU protein levels was observed in striatal mitochondria (Figs. S7a and S12) and a tendency for an increase in cortical mitochondria (Figs. S7b and S12) from SAPAP3-KO mouse brain. Similarly to Cx II subunit, enhanced protein levels of MCU in striatal mitochondria, and apparent increased MCU and OPA1 in cortical mitochondria from SAPAP3-KO mouse appear to act as compensatory mechanisms for the absence of SAPAP3 (Fig. S7a, b). No differences in these protein levels were observed in hippocampal (Figs. S7c and S12) or cerebellar (Figs. S7d and S12) mitochondria, in accordance with unchanged mitochondrial complexes activities or Ca^2+^ uptake (Fig. S6).

These results show that the lack of SAPAP3 has a significant impact on striatal and cortical mitochondrial function, suggesting a brain area-specific defect in MRC activity, with a concomitant deficiency in Ca^2+^ buffering capacity in SAPAP3-KO mice, despite enhanced levels of proteins involved in Cx II activity, mitochondrial Ca^2+^ uptake or cristae organization.

In addition, we observed alterations in mitochondrial morphology in the WT and SAPAP3-KO mouse striatum. As depicted in Fig. 3h, mitochondria from SAPAP3-KO mice exhibit decreased area, perimeter, Feret’s diameter and aspect ratio, and increased circularity and roundness compared to WT mouse mitochondria, suggesting enhanced mitochondrial fragmentation associated with the loss of SAPAP3.

### Mitochondrial SAPAP3 levels are altered in HD models

Previous studies using SAPAP3-KO mice revealed that SAPAP3 mediated-OCD like behaviors were associated with impairment in cortical-striatal pathway [3], features also shared in HD mouse models, like the YAC128 and the R6/2 mice [34, 35]. In HD, neuronal dysfunction has been linked to impaired mitochondrial function, a hallmark of the disease since early stages (e.g. [12]).

**Fig. 4:**
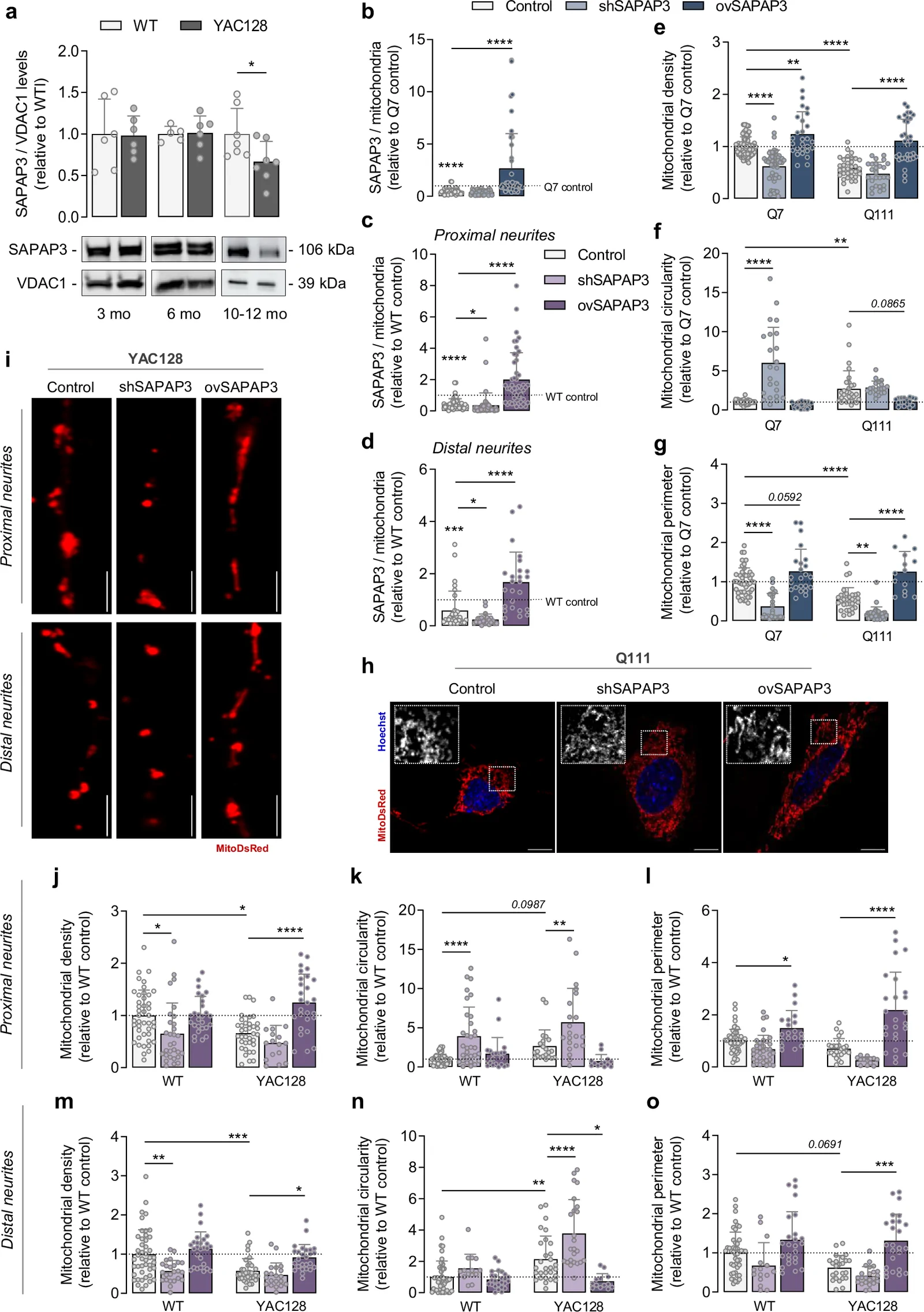
SAPAP3 levels within HD mitochondria and SAPAP3-dependent mitochondrial morphology in striatal cells. Mitochondrial fractions prepared from WT or YAC128 mouse striatum were probed with the affinity-purified SAPAP3 antibody, using VDAC1 as the loading control. Densitometric analysis of SAPAP3 in YAC128 mice relative to WT at 3, 6 and 10-12 months (**a**). Data are the mean ± SD of independent mitochondrial preparations derived from 5-7 mice. Striatal cells (Q7 or Q111 cells and WT or YAC128 neurons) were transfected with either GFP-control, -shSAPAP3 or -ovSAPAP3 plasmids. SAPAP3 levels in mitochondria were assessed in Q111 relative to Q7 control cells (**b**; representative images shown in Fig. S10a) and in YAC128 striatal proximal (**c**) and distal (**d**) neurites relative to WT control neurons (representative images shown in Fig. S10b). Mitochondrial density (**e**, **j**, **m**), circularity (**f**, **k**, **n**) and perimeter (**g**, **l**, **o**) were evaluated in Q7 or Q111 cell lines and WT or YAC128 proximal and distal neurites, respectively. Representative images of mitochondria (red, labelled by MitoDsRed) are shown in Q111 cells (**h**) and in YAC128 proximal and distal neurites (**i**). The complete panel of images is shown in Fig. S10c, d. Proximal neurites were defined within 50 μm of the soma ( < 50 μm), whereas distal neurites were analyzed at distances greater than 50 μm from the soma ( > 50 μm). Scale bar = 10 μm (**h**); 5 μm (**i**). Data are the mean ± SD of 3-4 independent experiments, considering 5-15 cells *per* condition. Statistical analysis: *p < 0.05, **p < 0.01, ***p < 0.001 and ****p < 0.0001 by Mann Whitney test, Kruskal-Wallis’s followed by uncorrected Dunn’s test, or two-way ANOVA followed by the Tukey’s multiple comparison test.

The protein levels of SAPAP3 were analyzed in HD human *postmortem* brain tissue revealing decreased SAPAP3 in the caudate, but not in the parietal cortex (Figs. S9a and S14). We also analysed SAPAP3 protein levels in isolated mitochondria and neuronal cultures derived from YAC128 mouse brain and in striatal Q111 cell line. A significant decrease in mitochondria-associated SAPAP3 was detected in symptomatic YAC128 mouse striatum (at 10-12 months of age) (Figs. 4a and S12), but not in cortical mitochondria (Figs. S9d and S15), when compared with age-matched WT mice. This is consistent with the decrease in SAPAP3 levels in total striatal fractions from 10-12 month-old YAC128 mice, which is not observed in total cortical tissue (Fig. S9b, c).

SAPAP3 differentially co-localizes with mitochondria in wild-type and HD striatal cells and neurons (Fig. S10a, b). A decrease in mitochondrial SAPAP3 levels was observed in striatal Q111 cells (Fig. 4b) and in YAC128 striatal proximal and distal neurites (Fig. 4c, d), in comparison to Q7 or WT control (depicted in dashed lines), respectively. Given that SAPAP3 associated with mitochondria is decreased in HD conditions, we sought to analyze the influence of modulating cellular SAPAP3 levels. Mitochondrial SAPAP3 levels were shown to be unaffected upon SAPAP3 silencing in Q111 cells (Fig. 4b); nevertheless, under the same conditions, an additional reduction of SAPAP3 in both proximal and distal neurites from YAC128 striatal neurons was observed (Fig. 4c, d). Moreover, ovSAPAP3 increased SAPAP3 protein levels within mitochondria as compared to control Q111 cells and YAC128 neurons (Fig. 4b–d).

These results demonstrate that SAPAP3 associates with mitochondria, that this association is significantly reduced in HD models, and that modulation of SAPAP3 levels directly impacts its mitochondrial localization.

### SAPAP3 modulates mitochondrial morphology in HD cells

Changes in mitochondrial morphology are described as causally linked to HD neuronal dysfunction [36, 37]. Our results showed a decrease in mitochondrial density in both Q111 cells and striatal YAC128 neurites (Fig. 4e, j, m), with Q111 cells presenting more circular and smaller mitochondria (Figs. 4f, g and S10c). Similarly, an increase in mitochondrial circularity was observed in distal neurites (Figs. 4n and S10d) and, at a lower extent, in proximal neurites (Figs. 4k and S10d) from striatal YAC128 neurons, whereas a tendency for a decrease in mitochondrial perimeter was observed only in distal neurites (Fig. 4o). These data indicate a fragmented mitochondrial network in Q111 cells and YAC128 mouse neurons.

SAPAP3 silencing induced a decrease in mitochondrial density in both Q7 cell line (Fig. 4e) and WT neurites (Fig. 4j, m), but no additional changes were observed in Q111 cells (Fig. 4e) or YAC128 neurites (Fig. 4j, m). Mitochondrial circularity was shown to be increased in proximal neurites from WT mice (Figs. 4k and S10d) and in Q7 cells (Figs. 4f and S10c), the latter exhibiting decreased mitochondrial perimeter (Fig. 4g), following shSAPAP3. Interestingly, shSAPAP3 decreased even further the mitochondrial perimeter of Q111 cells (Fig. 4g), and exacerbated the increased mitochondrial circularity observed in YAC128 neurites (Fig. 4i, k, n).

Upon ovSAPAP3, mitochondrial perimeter was significantly increased in proximal neurites from WT mice (Fig. 4l), but only slightly in Q7 cells (Fig. 4g), along with augmented mitochondrial density in Q7 cells (Fig. 4e). Of note, ovSAPAP3 increased mitochondrial density in both Q111 cells (Fig. 4e) and YAC128 neurites (Fig. 4j, m). ovSAPAP3 also increased mitochondrial perimeter in both Q111 cells (Fig. 4g) and YAC128 neurites (Fig. 4l, o); in contrast, mitochondrial circularity was significantly decreased in YAC128 distal (Fig. 4i, n), but not significantly in YAC128 proximal (Fig. 4k) neurites, and slightly reduced in Q111 cells (Fig. 4f, h) following ovSAPAP3.

In accordance with these data, Q111 cells showed increased levels of mitochondrial Drp1 fission protein (Fig. S11a, b) and decreased levels of OPA1 fusion protein (Fig. S11c, d) as compared to Q7 cells, which were exacerbated following SAPAP3 silencing (Fig. S11a–d). Of relevance, ovSAPAP3 restored both Drp1 and OPA1 protein levels (Fig. S11a–d).

These results demonstrate that silencing SAPAP3 mimics the HD fragmented mitochondrial network observed in Q111 cells and YAC128 neurons, which is rescued following SAPAP3 overexpression.

### SAPAP3 regulates mitochondrial dynamics in HD cells

**Fig. 5:**
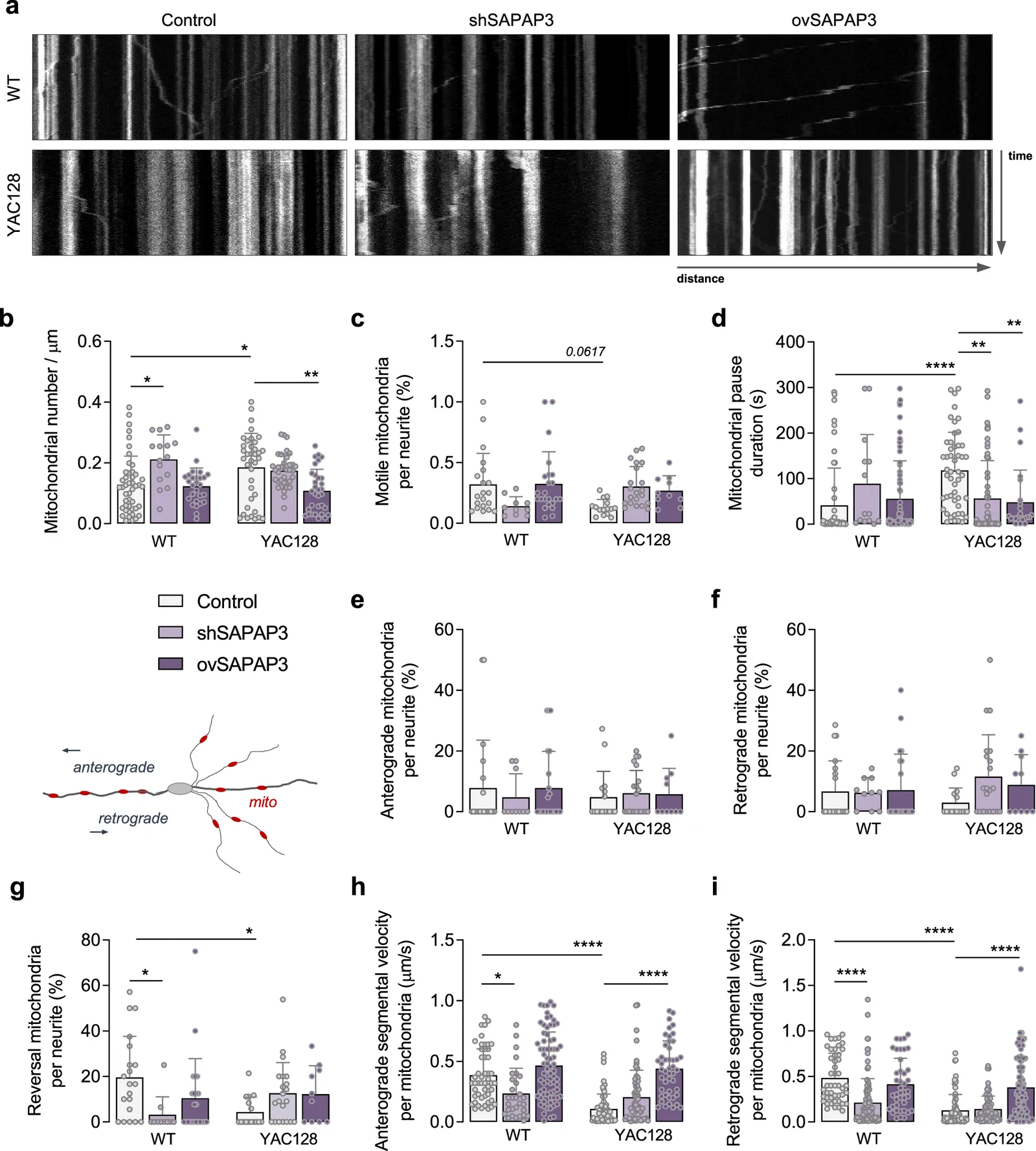
SAPAP3 influence on mitochondrial dynamics in striatal neurons. Striatal neurons from WT or YAC128 mice were transfected with either GFP-control, -shSAPAP3 or -ovSAPAP3 plasmids. Representative kymographs from time-lapse image sequences of moving mitochondria (**a**). Number of mitochondria per μm of imaged neurite (**b**). Percentage of mitochondrial motility per neurite (**c**). Quantification of mitochondrial pause duration (**d**). Percentage of anterograde (**e**), retrograde (**f**) or reversal (**g**) mitochondria per neurite. Quantification of the velocity of anterograde (**h**) or retrograde (**i**) mitochondria within a single segment. Only neurites having motile mitochondria were analysed. Data are the mean ± SD of 6-9 independent experiments. Statistical analysis: *p < 0.05, **p < 0.01 and ****p < 0.0001 by two-way ANOVA followed by the Tukey’s multiple comparison test.

Next, it was analysed whether SAPAP3 modulates mitochondrial transport along the neurites (Fig. 5a). Accordingly, we observed an increase in the mitochondrial number in YAC128 neurons, which was counteracted by ovSAPAP3; an increase in mitochondrial number was also detected in WT neurons upon shSAPAP3 (Fig. 5b). YAC128 mitochondria also exhibited a slight decrease in motility (Fig. 5c), along with an increase in pause duration (Fig. 5d). Although no major changes were observed in the percentage of motile mitochondria upon shSAPAP3 or ovSAPAP3 (Fig. 5c), both conditions decreased pause duration in YAC128, but not in WT neurons (Fig. 5d). Data showed no differences in the percentage of mitochondria moving anterogradely or retrogradely (Fig. 5e, f). However, YAC128 and shSAPAP3-WT neurons, but not ovSAPAP3-WT or ovSAPAP3-YAC128, presented a decrease in reversal mitochondria (Fig. 5g), which change their movement direction along the microtubules (Fig. 5g). Of relevance, both anterograde and retrograde mitochondrial segmental velocities were shown to be decreased in YAC128 neurons and WT neurons subjected to shSAPAP3; this effect on YAC128 neurites was counterbalanced after ovSAPAP3 (Fig. 5h, i). Mitochondrial number, percentage of reversal mitochondria, and anterograde and retrograde segmental velocities remained unaltered under shSAPAP3 in YAC128 neurons (Fig. 5b, g–i).

These results indicate that SAPAP3 can modulate mitochondrial dynamics; in particular, enhancing SAPAP3 levels ameliorates mitochondrial dynamics in YAC128 neurons by promoting mitochondrial movement along neurites.

### SAPAP3 silencing impairs mitochondrial function and calcium retention in striatal cells

**Fig. 6:**
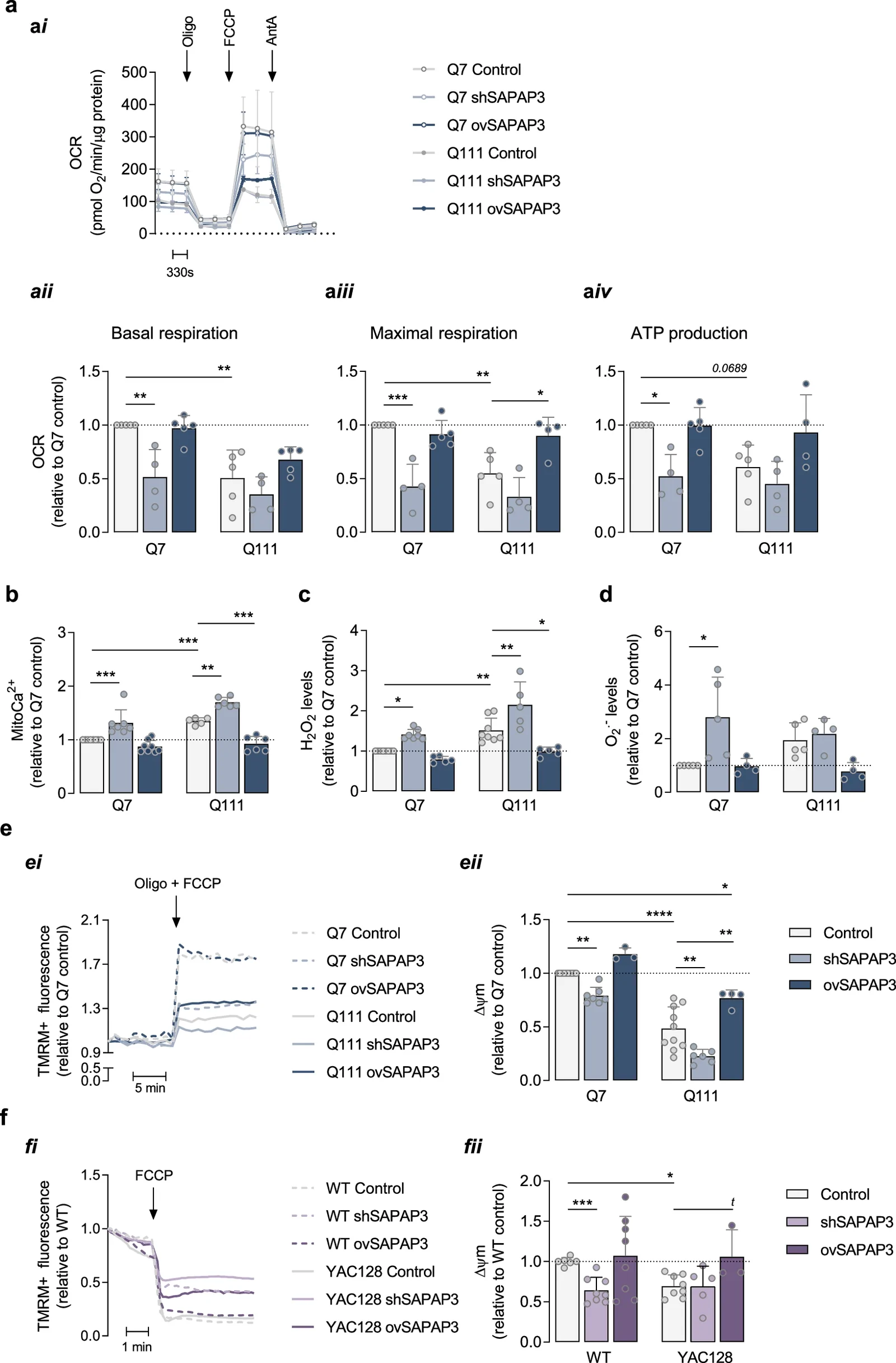
Effect of SAPAP3 on mitochondrial function in striatal cells. Striatal Q7 or Q111 cells and WT or YAC128 striatal neurons were transfected with either GFP-control, -shSAPAP3 or -ovSAPAP3 plasmids. Mitochondrial respiration was measured using a Seahorse flux analyser (**a**). OCR traces (**ai**) and quantification of basal respiration (**aii**), maximal respiration (**aiii**) and ATP production (**aiv**) following sequential additions of oligomycin (1 µM), FCCP (1 µM) and antimycin A (1 µM) plus rotenone (1 µM). Levels of mitochondrial Ca^2+^ (**b**), cellular H_2_O_2_ (**c**), mitochondrial O_2_^•–^ after exposure to myxothiazol (4 µM) (**d**) and ∆Ψm in response to oligomycin (2 µg/mL) plus FCCP (2 µM) (**e**) were evaluated using fluorescent probes. ∆Ψm in primary neurons (**f**) was assessed after FCCP (2 µM)-induced depolarization. Data are the mean ± S.D. of 3-10 independent experiments. Statistical analysis: *p < 0.05, **p < 0.01, ***p < 0.001 and ****p < 0.0001 by two-way ANOVA followed by the Tukey’s multiple comparison test and ^*t*^*p* < 0.05 by Mann Whitney test.

The role of SAPAP3 on mitochondrial function was also assessed. A decrease in mitochondrial basal and maximal respiration and ATP production was observed in Q111 cells and similar results were found under shSAPAP3 in Q7 cells (Fig. 6ai-iv). Overexpressing SAPAP3 recovered maximal respiration in Q111 cells (Fig. 6ai, iii), without significantly affecting basal respiration or ATP production (Fig. 6ai, ii, iv). On the other hand, no differences were found in Q7 cells upon ovSAPAP3 as compared to the control (Fig. 6ai-iv).

Moreover, our results indicate increased mitochondrial Ca^2+^ and H_2_O_2_ levels in Q111 cells, which were counteracted by ovSAPAP3, while silencing SAPAP3 exacerbated these effects (Fig. 6b–d). Moreover, ovSAPAP3 counteracted the decrease in ΔΨm in both Q111 cells (Fig. 6e) and YAC128 striatal neurons (Fig. 6f). In addition, increased mitochondrial Ca^2+^ retention, H_2_O_2_ and mitochondrial O_2_^•–^ levels and decreased ΔΨm were observed after shSAPAP3 in Q7 cells and WT neurons (Fig. 6b–f).

Our findings demonstrate that SAPAP3 has a major impact in regulating mitochondrial function in WT and HD neurons/cells.

## Discussion

In this study, we identify a previously unrecognized role for the postsynaptic scaffolding protein SAPAP3 in the regulation of mitochondrial function, particularly in the context of HD. Our findings demonstrate that SAPAP3 contributes to mitochondrial dynamics and activity, thereby establishing a mechanistic link between altered levels of a postsynaptic scaffolding protein and mitochondrial impairment in HD pathology.

Deregulation of mitochondrial function is a hallmark of several neurological disorders associated with compromised synaptic transmission, a fundamental mechanism that affects processing information in the brain. The interplay between PSD proteins and mitochondria has previously been reported. Proteomic studies of PSD-enriched fractions from the cingulate cortex of individuals with bipolar disorder revealed altered expression of proteins implicated in mitochondrial oxidative phosphorylation and calcium signalling [16]. Similarly, synaptic proteomic profiling of individuals with schizophrenia and bipolar disorder identified selective downregulation of proteins related to synaptic and mitochondrial function [17], linking PSD-associated components and synaptic function to mitochondrial-driven energy pathways.

Our interactome analysis identifies Mic60 as the most enriched mitochondria-associated SAPAP3 interactor. At the cellular level, SAPAP3 silencing in striatal cells resulted in reduced Mic60 protein levels and impaired SAPAP3-Mic60 colocalization, suggesting a functional association between these proteins. A direct or indirect interaction between SAPAP3 and Mic60 was further supported by PLA and immunoprecipitation analyses. Mic60 is a core component of the MICOS complex and plays a central role in cristae organization, mitochondrial morphology and organelle dynamics [10, 11]. Consistent with this, mitochondria from Mic60-deficient HeLa cells exhibited disrupted cristae structure, affecting the integrity of MICOS and its interaction with Sam50, which are relevant for cristae organization and mitochondrial function [38]. Also, Mic60 acts as key player in regulating mitochondrial dynamics and morphology [39]. Therefore, the present findings uncover a previously unknown connection between SAPAP3 and mitochondrial physiology and morphology regulation.

Association of SAPAP3 with mitochondrial components, particularly in the striatum, might indicate a broader role in cellular homeostasis, potentially linking postsynaptic dysfunction with mitochondrial abnormalities, as observed in neuropsychiatric disorders such as OCD [3, 40, 41] and in HD, in which OCD was also described (e.g., [42, 43]). Although dendritic mitochondrial organization was shown to be independent of dendritic spine morphology, mitochondria associated with spines may support synaptic strength and postsynaptic signalling, as calcium buffering [44]. Therefore, discrete proteomic profiles may characterize mitochondrial subcellular localization and affect synaptic morphology [45], influencing local organelle activity.

Mitochondrial functional analysis in SAPAP3-KO mice provides evidence for the involvement of this protein in mitochondrial activity. The impairment in MRC complexes activities (Cx I-III) and in calcium handling in mitochondria isolated from the cortex and striatum of SAPAP3-KO mice, while hippocampal and cerebellar mitochondria were largely unaffected, strongly suggests a brain region-specific mitochondrial dysfunction. This observation highlights a critical role for SAPAP3 in mitochondria in the cortico-striatal circuitry. The relevance of SAPAP3 in this circuitry was previously demonstrated, as synaptic and behavioural deficits in SAPAP3-mutant mice were rescued by selective expression of SAPAP3 in the striatum [3]. It should also be noted that brain isolated mitochondria used in this study include mitochondria originating from different cell types. Apart from its neuronal location, recent evidence showed high levels of SAPAP3 in striatal astrocytes, particularly in astrocyte sub-compartments regulating actin cytoskeleton organization [46]. These data suggest that SAPAP3-dependent mitochondrial regulation may extend beyond neurons.

The differences in protein levels between brain circuits may reflect a redundancy and compensatory role of other SAPAP family members [47]. The upregulation of selected mitochondrial proteins in SAPAP3-deficient striatum and cortex, including respiratory chain subunits highlights a mechanism to partially counterbalance mitochondrial dysfunction. Increased MCU protein levels in the striatum and the cortex may promote mitochondrial Ca^2+^ overload and oxidative stress. Elevation of MCU-induced Ca^2+^ levels was demonstrated to be a major cause of mitochondrial ROS, leading to altered cellular metabolism [48]. Interestingly, Mic60 levels were decreased in SAPAP3-KO striatal mitochondria and increased in cortical mitochondria, which may reflect compensatory mechanisms or alternative pathways of mitochondrial regulation in the absence of SAPAP3, underscoring the complexity of mitochondrial modulation. However, such mechanisms are largely insufficient to restore normal mitochondrial function, as evidenced by the defects in complex activities and Ca^2+^ handling. Importantly, ultrastructural analyses confirmed enhanced mitochondrial fragmentation in SAPAP3-KO striatum, reinforcing the functional deficits observed.

These findings are particularly relevant given the role of cortico-striatal connectivity in motor and cognitive functions, which are disrupted in traumatic brain injury or in HD, among other pathological conditions, contributing to cognitive and executive dysfunction [49, 50]. These data suggest that SAPAP3 plays a critical, non-redundant role in maintaining mitochondrial function, particularly in the striatum and cortex.

In HD models, SAPAP3 protein levels were reduced in human HD caudate tissue and in striatal tissue and mitochondria from YAC128 mice. SAPAP3’s role in HD mitochondrial function and dynamics is also critical. Notably, mitochondrial phenotypes observed in HD cells closely mirrored those induced by SAPAP3 silencing in WT cells. Indeed, functional analyses in HD striatal cells revealed mitochondrial alterations resulting from SAPAP3 modulation. Accordingly, restoration of SAPAP3 expression in HD cells (*via* ovSAPAP3) consistently rescued mitochondrial activity, namely oxygen consumption and transmembrane potential, mitochondrial movement and morphology, decreased ROS levels, and ameliorated mitochondrial Ca^2+^ retention. Previous studies demonstrated that physical proximity between mitochondria and synapses is regulated by neuronal activity [51, 52]. We show that SAPAP3 silencing diminishes mitochondrial transport within neurites, possibly contributing to mitochondrial deregulation and synaptic malfunction. Additionally, enhanced mitochondrial number in shSAPAP3 and YAC128 neurons is concordant with a more fragmented mitochondrial network, while ovSAPAP3 in HD neurons reduces mitochondrial number, aligning with WT-like tubular morphology. Restoration of normal mitochondrial dynamics (movement and morphology) upon SAPAP3 overexpression in YAC128 neurons suggests that SAPAP3 is a key modulator of mitochondrial health. Interestingly, Mic60 has been correlated with the regulation of mitochondrial motility, mainly by its interaction with Miro proteins [53]. Miro1 and Miro2 Rho-GTPases control mitochondrial transport along microtubules [54], and our proteomic data also show Miro2 (also referred as RhoT2) as a SAPAP3 interactor, providing a link between SAPAP3 and mitochondrial trafficking regulation.

Given the well-recognized role of SAPAP3 in synaptic organization, it is intriguing to consider that SAPAP3 might serve as a bridge between synaptic signaling and mitochondrial health, which is affected in the presence of mHTT. In this context, the present study strongly suggests that SAPAP3 plays a critical role in maintaining mitochondrial integrity, particularly in the context of HD. This dual role of SAPAP3 might help explain the complex striatal neuropathology of HD, where both synaptic and mitochondrial dysfunctions are prominent features. Thus, further research is needed to fully elucidate the molecular mechanisms underlying SAPAP3’s influence on postsynaptic mitochondria, which will be essential for understanding how synaptic-mitochondrial coupling contributes to HD progression.

In conclusion, our study demonstrates that the postsynaptic scaffolding protein SAPAP3 associates with mitochondrial proteins, including Mic60, and regulates mitochondrial structure, dynamics and function. Reduced mitochondrial-associated SAPAP3 in cells expressing full-length mHTT/mHtt contributes to functional and morphological deficits, whereas restoration of SAPAP3 ameliorates disease-associated mitochondrial phenotypes. These results identify SAPAP3 as a previously unrecognized regulator of mitochondrial integrity and suggest its potential relevance as a therapeutic target in HD.

## Supplementary information

Supplementary material

Dataset 2

## Data Availability

All data generated and analysed in this study are included in this published article and Supplementary Information file. Mass spectrometry proteomics data were deposited at the ProteomeXchange Consortium via the PRIDE partner repository with the dataset identifier PXD023969. All remaining data can be made available upon reasonable request.
