# Supplementary material for: The postsynaptic scaffolding protein SAPAP3 shapes mitochondrial activity: the case of Huntington’s disease
Source: Cell Death Dis. 2026 Aug 7;17(1):936. doi: 10.1038/s41419-026-09156-4 (PMC13731651; doi:10.1038/s41419-026-09156-4)
Supplement: Supplementary file 1 [file 41419_2026_9156_MOESM1_ESM.pdf]

Supplementary Materials

Title: "The postsynaptic scaffolding protein SAPAP3 shapes mitochondrial activity: the case of Huntington's disease**"**

This file contains the Supplementary Methods, Supplementary Figures S1–S15, and Supplementary Tables S1 and S2, plus the respective legends. Figures S12–S15 show the original full-length (uncropped) Western blots.

1. Supplementary Methods

ESPRIT

After random truncation in both the 3' and 5' ends of SAPAP3 gene, the DNA fragments with sizes corresponding to SAPAP3 regions ranging from 100 to 400 amino acids were excised, ligated and transformed into *E. coli*.

E. coli BL21 AI cells (Thermo Fisher Scientific) were transformed with the library and 27,642 clones picked robotically into 72 384-well plates, where cells were grown, and then arrayed in duplicate onto nitrocellulose membranes (Cytiva) that were laid over LB agar with kanamycin and chloramphenicol for colony growth. After induction of protein expression colonies on membranes were lysed and probed using anti-His (Cytiva) and rabbit anti-mouse Alexa Fluor 532 conjugate (Thermo Fisher Scientific) to detect the N-terminal hexahistidine tag, and streptavidin Alexa Fluor 488 (Thermo Fisher Scientific) to detect the C-terminal biotinylation of soluble fragments. The 3545 clones with the highest anti-His signal were ranked based on their streptavidin signal, with the best 96 signals selected for expression and nickel-NTA purification analyses in larger scale cultures.

The selected 96 clones were grown, their expression induced, and the protein was robotically purified using a 96-well filter plate with 60 µL Ni-NTA Agarose (Qiagen) in

each well. After elution, the proteins were analysed by streptavidin blot using Alexa Fluor 488 labelled streptavidin, from which the 20 most promising clones were selected for Sanger DNA sequencing and scale-up expression in 50 mL cultures. The protein from such expressions was purified by IMAC in batch mode using 300 µL Ni-NTA Agarose, being consecutively eluted with 100 mM and 300 mM imidazole, followed by Coomassie-stained SDS-PAGE analysis. The most promising constructs were selected for larger-scale protein production.

Protein expression and purification

E. coli BL21 AI (Thermo Fisher Scientific) transformed with the desired construct were grown in 1 L Luria Broth (Miller's Base) supplemented with kanamycin and 50 µM biotin at 37°C. After cell culture OD_{600} reached 0.6 – 0.8, the temperature was lowered to 25 °C, L-arabinose added to a final concentration of 0.2% (w/v), and protein expression was allowed to occur overnight. The cells were harvested by centrifugation and resuspended in lysis buffer (20 mM sodium phosphate, pH 7.4, 500 mM NaCl, 10% (v/v) glycerol, 25 mM imidazole, 0.1 M phenylmethylsulfonyl fluoride (PMSF; Thermo Fisher, Waltham, MA, USA) followed by mechanical disruption using a high-pressure homogeniser (Emulsiflex-C3 – Avestin, Ottawa, Canada), with 3 passages at 1 000 bar.

The cellular extract was clarified by centrifugation, and the supernatant was applied onto a 5 mL HisTrap HP column (Cytiva), previously equilibrated with the lysis buffer, using an ÄKTA system (Cytiva). After baseline stabilisation, protein elution was performed by a four-step gradient of imidazole (50, 150, 300, 500 mM). The fractions eluting at 150 mM imidazole gradient step were pooled and applied onto a HiLoad Superdex 200 26/60 prep-grade size exclusion chromatography (SEC) column (Cytiva),

previously equilibrated at 2 mL/min in 20 mM sodium phosphate pH 7.4, 150 mM NaCl, 10% (v/v) glycerol. The fractions from the main peak were pooled, and a final polishing step was done by ion-exchange chromatography (IEX) using a Mono S 5/50 GL column (Cytiva), equilibrated with 20 mM sodium phosphate pH 7.4, 10% (v/v) glycerol. Protein elution was obtained with a 0-1 M NaCl linear gradient.

Protein quality control

Protein quality was evaluated by SDS-PAGE. The protein oligomerization state was evaluated by analytical SEC in an HPLC (Shimadzu Prominence), equipped with a photodiode array, using a Superdex 200 Increase 5/150 GL column (Cytiva) equilibrated with 20 mM sodium phosphate pH 7.4, 300 mM NaCl, 10% (v/v) glycerol.

Secondary structure content was evaluated by Circular Dichroism using a Jasco J-815 spectropolarimeter, with spectra acquired between 190 and 260 nm. Each spectrum was the average of six scans with the average buffer spectrum subtracted.

SWATH-MS data acquisition and analysis

Eight µL of each denatured sample were pooled to generate the proteome library (protein composition of each condition), and 22 µL of each sample were used separately for SWATH-MS analysis to extract quantitative information. Samples were alkylated with acrylamide and subjected to in-gel digestion by using the short-GeLC approach **[1]**. The generated peptides were subjected to SPE using OMIX tips with C18 stationary phase (Agilent Technologies) as recommended by the manufacturer and then resuspended in 30 µL of liquid chromatography mobile phase (2% (v/v) acetonitrile and 0.1% (v/v) formic acid).

Samples were analysed on a Triple TOF^{TM} 5600 System (Sciex, Framingham, MA - USA) in two phases: information-dependent acquisition (IDA) of the pooled samples for protein identification and SWATH (Sequential Windowed data-independent Acquisition of the Total High-resolution Mass Spectra) acquisition of each individual sample for protein quantification. Peptides were resolved by liquid chromatography (nanoLC Ultra 2D, Eksigent®) on a ChromXP™ C18 CL reverse phase column (300 µm ID × 15 cm length, 3 µm particles, 120 Å pore size, Eksigent®) at 5 µL/min. Peptides were eluted into the mass spectrometer with an acetonitrile linear gradient in 0.1% formic acid (5% to 35% acetonitrile for 45 min), using an electrospray ionization source (DuoSpray™ Source, Sciex).

For IDA experiments the mass spectrometer was set to scanning full spectra (350-1250 m/z) for 250 ms, followed by up to 100 MS/MS scans (100–1500 m/z from a dynamic accumulation time – minimum 30 ms for precursor above the intensity threshold of 1000 – in order to maintain a cycle time of 3.3 s). Candidate ions with a charge state between +2 and +5 and counts above a minimum threshold of 10 counts per second were isolated for fragmentation and one MS/MS spectra was collected before adding those ions to the exclusion list for 25 seconds (mass spectrometer operated by Analyst® TF 1.6, Sciex). The rolling collision was used with a collision energy spread of 5. An additional IDA experiment was performed for each pool to improve the sample coverage using an exclusion list of the peptides previously identified.

For SWATH-MS based experiments, the mass spectrometer was operated in a looped product ion mode **[2]** and the same chromatographic conditions used as in the DDA run described above. The SWATH-MS setup was designed specifically for the samples to be analysed using the SWATH Variable Window Calculator_V1.0 (Sciex®) (**Table S2**). A set

of 60 windows of variable width (containing 1 m/z for the window overlap) was constructed, covering the precursor mass range of 350-1250 m/z. A 100 ms survey scan (350-1500 m/z) was acquired at the beginning of each cycle for instrument calibration and SWATH-MS/MS spectra were collected from 100–1500 m/z for 50 ms resulting in a cycle time of 3.15 s from the precursors ranging from 350 to 1250 m/z. The collision energy for each window was determined according to the calculation for a charge +2 ion centred upon the window with a collision energy spread of 15.

A specific library of precursor masses and fragment ions was created by combining all files from the IDA experiments, and used for subsequent SWATH processing. Libraries were obtained using Protein Pilot^{TM} software (v5.0, Sciex) using the following search parameters: database of *Rattus norvegicus* protein sequences (from UniProt database release in January 2015) and MBP-GFP protein sequence; trypsin as digestion type; and acrylamide alkylated cysteines as a fixed modification. An independent False Discovery Rate (FDR) analysis using the target-decoy approach provided with Protein Pilot software was used to assess the quality of the identifications, and positive identifications were considered when identified proteins and peptides reached a 5% local FDR **[3,4]**.

SWATH data processing was performed using SWATH^{TM} processing plug-in for PeakView^{TM} (v2.0.01, Sciex). Briefly, peptides were selected automatically from the library, and up to 15 peptides with up to 5 fragment ions were chosen per protein. Quantitation was attempted for all proteins in the library file that were identified below 5% local FDR from ProteinPilot^{TM} searches by extracting the peak areas of the target fragment ions of those peptides using an extracted-ion chromatogram (XIC) window of 3 minutes with 20 mDa XIC width. All the peptides that met the 1% FDR threshold in at least three biological replicates were retained, and the levels of the proteins were

estimated by summing all the respective transitions for the peptides that met the criteria (an adaptation of **[5]**. The mass spectrometry proteomics data have been deposited at the ProteomeXchange Consortium via the PRIDE **[6]** partner repository with the dataset identifier: PXD023969.

For comparisons between experiments, the protein levels were normalised to the internal standard (MBP-GFP) **[7,8]**. To identify the potential interactors of SAPAP3_G^{754}-I^{968} domain, the normalised protein levels were processed using the Perseus 1.6.14.0 Software (Max Planck Institute of Biochemistry). After data $\log_2$ transformation, a Student's t-test was used to test the differences between groups, and a false discovery rate (FDR or q-value) below 0.05 was considered to be significant. Gene ontology analysis was performed with BiNGO app (v. 3.0.4 **[9]**) in Cytoscape (v. 3.8.2) for the list of the 30 genes identified with a statistically significant difference between the two experimental conditions. The analysis was done using the hypergeometric statistical test, with Benjamini & Hochberg FDR correction, a significance level of 0.05, with the whole annotation as reference set, *Rattus novergicus* as organism, and GO_cellular component as ontology file.

Genotyping

Embryos were genotyped by a PCR standard procedure using LyA1 (CCTGCTCGCTTCGCTACTTGGAGC), LyA2 (GTCTTGCGCCTTAAACCAACTTGG), RyA1 (CTTGAGATCGGGCGTTCGACTCGC), RyA2 (CCGCACCTGTGGCGCCGGTGATGC) and actin (F-GGAGACGGGGTCACCCACAC, R-AGCCTCAGGGCATCGGAACC) primers (Thermo Fisher Scientific). PCR protocol consisted in 35 cycles, with the following temperatures and times for each step: denaturation at 95 °C for 30 s, annealing at 61 °C for 30 s and

elongation at 72 °C for 30 s. PCR products were analyzed on 2% (w/v) agarose gel and visualized under a UV trans-illuminator Gel Doc XR system (Bio-Rad).

Cellular metabolic and death assays

For analysis of cell metabolic activity, striatal cells were incubated with 0.5 mg/mL 3-4,5- dimethylthiazol-2-yl)-2,5diphenyltetrazolium bromide (MTT; Sigma, St Louis, MO, USA) in Na^+ medium (140 mM NaCl, 5 mM KCl, 0.4 mM KH_2PO_4, 1 mM $CaCl_2$, 1 mM $MgCl_2$, 10 mM glucose, 10 mM HEPES, pH 7.4/NaOH) for 4 h at 33 °C and the resulting formazan crystals were dissolved in 0.04 M HCl in isopropanol. Absorbance was measured at 570 nm using a SpectraMAX Plus microplate reader (Molecular Devices). MTT reduction was normalized to the respective control.

For cell death analysis, cells were incubated with 4 µg/mL Hoechst 33342 (Thermo Fisher, Waltham, MA USA) and 2 µg/mL propidium iodide (PI, Thermo Fisher, Waltham, MA USA) in the dark for 10 min at room temperature, then washed with phosphate-buffered saline (PBS: 137 mM NaCl, 2.7 mM KCl, 1.8 mM KH_2PO_4, 10 mM $Na_2HPO_4.2H_2O$, pH 7.4), fixed with 4% (w/v) paraformaldehyde for 15 minutes and mounted using Mowiol 40-88 (Sigma, St Louis, MO, USA). Images were taken using a plan-apochromat/0.95NA 40x lens on a Carl Zeiss Axio Observer.Z1 upright microscope. Nuclear condensation was used to distinguish apoptotic cell and PI-positive cells were identified as necrotic cells using FIJI software (ImageJ).

Total extracts

Total extracts from *postmortem* HD or control human caudate and parietal cortex or YAC128 mice or WT striatum and cortex at 3-, 9 and 10-12 month old were performed

in Ripa buffer (containing: 150 mM NaCl, 50 mM Tris, 5 mM EGTA, 1% Triton X- 100, 0.5% DOC, 0.1% SDS) freshly supplemented with 1 mM dithiothreitol (DTT), 1 mM phenylmethylsulphonyl fluoride (PMSF), 100 nM okadaic acid, 25 mM sodium fluoride (NaF), 1 mM Na_3VO_4 and 1 µg/mL protease inhibitors (chymostatin, pepstatin A, leupeptin and antipain). Then, cell extracts were further collected in chilled clean tubes and centrifuged at 20,800 *x* g for 10 min at 4 °C. The supernatant (total extracts) was then submitted to protein quantification by BioRad protein assay and stored at −80°C until use. Samples were then subjected to western botting analysis as described in "Mitochondrial-enriched extracts and western blotting" described below.

Mitochondrial-enriched extracts and western blotting

Mitochondrial samples were boiled in 1% (w/v) SDS for 10 min and then denatured with 6x loading buffer (300 mM Tris-HCl pH 6.8, 12% (w/v) SDS, 30% (v/v) glycerol, 600 mM DTT, 0.06% (w/v) bromophenol blue) at 95 °C for 5 min. Equivalent amounts of protein (20 µg) were separated in 10% SDS-PAGE gel electrophoresis and electroblotted onto polyvinylidene difluoride (PVDF) membranes (BD Biosciences, Franklin Lakes, NJ, USA). The membranes were blocked with 5% (w/v) skim milk prepared in TBS-Tween (25 mM Tris-HCl, 150 mM NaCl, pH 7.6 / 0.1% (v/v) Tween-20), for 1 h at room temperature and further incubated overnight at 4 °C (with gentle agitation) with primary antibodies prepared in 5% BSA (w/v) (rabbit SAPAP3, 1:250) or 1% skim milk (w/v) [VDAC1, 1:500, (Abcam, Cambridge, UK); 70 kDa-CxII subunit, 1:10,000 (Abcam, Cambridge, UK); MCU, 1:1,000; Mic60, 1:500; OPA1, 1:500 (BD Biosciences, Franklin Lakes, NJ, USA0) and total OXPHOS antibody cocktail, 1:1,000 (Abcam; #ab110413). Membranes were then incubated with anti-mouse and anti-rabbit alkaline phosphatase secondary antibodies

(GE Healthcare, Chicago, IL, USA) prepared in 1% (w/v) skim milk-containing TBS-Tween for 1 h, at room temperature. To normalise the amount of protein *per* lane, anti-VDAC1 was used as the loading control. Immunoreactive bands were visualised by alkaline phosphatase activity after incubation with ECF substrate (GE Healthcare, Chicago, IL, USA), in a Bio-Rad ChemiDoc Imaging System (Bio-Rad). Bands were quantified using the Image Lab 6.0.1 software (Bio-Rad).

Mic60 affinity purification and SAPAP3 detection by western blotting

Immunoprecipitation (IP) of Mic60 was performed using the Dynabeads® Co-Immunoprecipitation Kit (Invitrogen), according to the manufacture instructions. Antibody-bead coupling was carried out in advanced using 5 µg of antibody per mg of beads, and 1 mg of Dynabeads® M-270 Epoxy was used per IP, as recommended. The antibodies used were a rabbit polyclonal anti-Mic60 (10179-1-AP; Proteintech® Europe) and normal rabbit IgG (sc-2027; Santa Cruz Biotechnology, Inc.) as a negative control.

Striatal samples of WT mice at 2 and 6 months of age were resuspended in PBS buffer supplemented with 1 mM of sodium fluoride (NaF) and 1 mM of sodium orthovanadate (Na_3VO_4) and further homogeneized. Lysates were then diluted 1:1 with 2x IP buffer (supplied with the Co-Immunoprecipitation kit) supplemented with 2% (v/v) Triton X-100 plus protease inhibitor mixture (cOmplete™, Roche). Samples were incubated on ice for 15 min to enhance membrane lysis, followed by centrifugation at 500 × *g* for 5 min at 4 °C. Supernatants were collected and protein concentration determined using the Pierce™ Protein Assay (Thermo Fisher Scientific). Sample extracts were equally divided into IP Mic60 and negative control (IP-). Samples were incubated overnight at 4 °C under constant agitation with the antibody coupled beads. After incubation, beads

were washed three times with 500 µL of IP buffer to minimize nonspecific binding. Bound proteins, including the antibodies, were eluted by adding 30 µL of Laemmli sample buffer and heating at 95 °C for 5 min. The eluted proteins were loaded and separated by SDS-PAGE on a 7.5% polyacrylamide gel. Electrophoresis was stopped to retain the antibodies heavy chain (IgH) to be used as a control to confirm equal antibody amounts in the IP Mic60 and IP- samples. Proteins were transferred onto PVDF membrane. The membrane was then incubated with a rabbit anti-SAPAP3 antibody (kindly provided by the Guoping Feng lab; 1:250 dilution) overnight at 4 °C followed by 1 h at RT and further with a fluorescence-conjugated secondary antibody (StarBright Blue 700 Goat Anti-Rabbit IgG, reference 12004162, Bio-Rad; 1:1,000 dilution).

After signal acquisition, the membrane was re-incubated with anti-Mic60 antibody (110329, Abcam; 1:500 dilution). After, the membrane was incubated with a phosphatase-conjugated mouse secondary antibody (115-055-003, Jackson ImmunoResearch Laboratories, Inc; 1:10,000 dilution) and the immunoreactive bands were detected using the "Enhanced Chemifluorescence (ECF) detection system" (GE Healthcare).

Fluorescent signal was acquired after complete membrane drying using an Azure c600 imaging system (Azure Biosystems). All the antibodies were prepared in blocking solution.

Transmission electron microscopy (TEM)

About 1 mm^3 of striatal tissue were fixed with 2.5% glutaraldehyde in 0.1 M sodium cacodylate buffer (pH 7.2) for 2 h or overnight at 4°C. Following rinsing in the same buffer, post-fixation was performed using 1% osmium tetroxide for 1 h. After rinsing, 1%

aqueous uranyl acetate was added during 1 h for contrast enhancement and then dehydrated in a graded ethanol series (70–100%). Following embedding in 2% molten agar, cell pellets were re-dehydrated in ethanol (30-100%), impregnated and included in Epoxy resin (Fluka Analytical). Ultrathin sections were mounted on copper grids and stained with lead citrate 0.2%, for 7 min. Observations were carried out on a FEI Tecnai G2 Spirit BioTwin electron microscope at 100 kV at the Bio-Imaging and Electron Microscopy Service at Institute for Biomedical Imaging and Life Sciences, University of Coimbra. Mitochondria was defined by free-hand tool and analysed with ROI manager of area, perimeter, Feret's diameter, circularity, roundness and aspect ratio by Fiji-imageJ software with the scale of 0.2pixel/nm. **[10].**

References

1. Anjo SI, Santa C, Manadas B. Short GeLC-SWATH: a fast and reliable quantitative approach for proteomic screenings. Proteomics. 2015;15:757-62.

2. Gillet LC, Navarro P, Tate S, Röst H, Selevsek N, Reiter L, et al. Targeted data extraction of the MS/MS spectra generated by data-independent acquisition: a new concept for consistent and accurate proteome analysis. Mol Cell Proteomics. 2012;11:O111.016717.

3. Sennels L, Bukowski-Wills J-C, Rappsilber J. Improved results in proteomics by use of local and peptide-class specific false discovery rates. BMC Bioinformatics. 2009;10:179.

4. Tang WH, Shilov Iv, Seymour SL. Nonlinear fitting method for determining local false discovery rates from decoy database searches. J Proteome Res. 2008;7:3661-7.

5. Collins BC, Gillet LC, Rosenberger G, Röst HL, Vichalkovski A, Gstaiger M, Aebersold R. Quantifying protein interaction dynamics by SWATH mass spectrometry: application to the 14-3-3 system. Nat Methods. 2013;10:1246-53.

6. Perez-Riverol Y, Csordas A, Bai J, Bernal-Llinares M, Hewapathirana S, Kundu DJ, et al. The PRIDE database and related tools and resources in 2019: improving support for quantification data. Nucleic Acids Res. 2019;47:D442-50.

7. Anjo SI, Simões I, Castanheira P, Grãos M, Manadas B. Use of recombinant proteins as a simple and robust normalization method for untargeted proteomics screening: exhaustive performance assessment. Talanta. 2019;205:120163.

8. Anjo SI, Martins-Marques T, Pereira P, Girão H, Manadas B. Elucidation of the dynamic nature of interactome networks: A practical tutorial. J Proteomics. 2018;171:116-26.

9. Maere S, Heymans K, Kuiper M. BiNGO: a Cytoscape plugin to assess overrepresentation of Gene Ontology categories in biological networks. Bioinformatics. 2005;21:3448-9.

10. Lam J, Katti P, Biete M, Mungai M, AshShareef S, Neikirk K, Garza Lopez E, Vue Z, Christensen TA, Beasley HK, Rodman TA, Murray SA, Salisbury JL, Glancy B, Shao J, Pereira RO, Abel ED, Hinton A Jr. A Universal Approach to Analyzing Transmission Electron Microscopy with ImageJ. Cells. 2021 Aug 24;10(9):2177.

2. Supplementary Figures (S1-S15) and Tables plus Legends

```
1     GGCGCGCCAT GCGTGGCTAT CATGGTGACC GTGGCTCGCA CCCGCGTCCG
51    GCCCGTTTTG CAGACCAGCA GCACATGGAT GTCGGCCCGG CAGCGCGCGC
101   CCCGTATCTG CTGGGTTCGC GTGAAGCATT TAGCACCGAA CCGCGCTTCT
151   GTGCACCGCG CGCTGGTCTG GGCCATATTT CCCCGGAAGG CCCGCTGTCG
201   CTGAGCGAAG GTCCGTCAGT GGGCCCGGAA GGCGGTCCGG CCGGTGCCGG
251   CGTTGGCGGT GGCAGCTCTA CCTTTCCGCG TATGTATCCG GGTCAGGGTC
301   CGTTTGACAC CTGCGAAGAT TGTGTTGGTC ATCCGCAGGG TAAAGGCGCC
351   CCGCGTCTGC CGCCGACCCT GCTGGACCAG TTTGAAAAGC AACTGCCGGT
401   TCAGCAAGAT GGTTTCCACA CGCTGCCGTA TCAGCGTGGT CCGGCAGGTG
451   CTGGTCCGGG TCCGGCTCCG GGCACCGGTA CGGCGCCGGA ACCGCGTTCT
501   GAAAGTCCGT CCCGTATCCG CCATCTGGTG CACAGCGTTC AGAAACTGTT
551   TGCCAAGTCA CATTCGCTGG AAGCACCGGG CAAGCGCGAC TACAACGGTC
601   CGAAAGCGGA AGGCCGTGGT GGCTCTGGTG GCGATAGTTA TCCGGGTCCG
651   GGTTCCGGTG GCCCGCACAC CTCACATCAC CACCATCATC ACCACCACCA
701   CCACCATCAC CAGTCTCGTC ATGGTAAACG CAGCAAATCT AAGGACCGTA
751   AAGGCGATGG TCGCCACCAG GCGAAGAGTA CCGGTTGGTG GAGTTCCGAT
801   GACAATCTGG ACAGTGATTC CGGTTTTCTG GCCGGTGGCC GCCCGCCGGG
851   TGAACCGGGT GGCCCGTTCT GCCTGGAAGG CCCGGATGGC TCTTACCGTG
901   ATCTGAGTTT TAAAGGTCGC AGCGGTGGCT CTGAAGGCCG TTGCCTGGCG
951   TGTACCGGCA TGTCAATGTC GCTGGACGGC CAGTCCGTTA AACGCTCAGC
1001  TTGGCATACG ATGATGGTCA GCCAGGGTCG TGATGGTTAT CCGGGTGCCG
1051  GTCCGGGTAA AGGTCTGCTG GGCCCGGAAA CCAAAGCGAA GGCCCGCACG
1101  TATCACTACC TGCAGGTGCC GCAAGATGAC TGGGGTGGCT ACCCGACCGG
1151  TGGCAAAGAT GGCGAAATTC CGTGTCGTCG CATGCGTAGC GGTTCTTATA
1201  TCAAAGCGAT GGGCGATGAA GAAAGTGGTG ACTCCGATGG CTCACCGAAA
1251  ACGAGCCCGA AGGCAGTTGC TCGTCGCTTC ACCACGCGTC GCTCATCGAG
1301  CGTCGATCAG GCACGCATTA ACTGCTGTGT GCCGCCGCGT ATTCATCCGC
1351  GCTCTAGTAT CCCGGGTTAC AGTCGTTCCC TGACCACGGG CCAGCTGAGC
1401  GATGAACTGA ATCAGCAACT GGAAGCCGTC TGCGGTTCCG TGTTCGGCGA
1451  ACTGGAATCA CAGGCCGTGG ACGCCCTGGA TCTGCCGGGT TGTTTTCGTA
1501  TGCGCTCACA CTCGTATCTG CGCGCAATTC AGGCTGGCTG CAGCCAAGAT
1551  GACGATTGTC TGCCGCTGCT GGCGACCCCG GCCGCAGTTA GCGGTCGCCC
1601  GGGCTCCTCA TTTAACTTCC GTAAAGCACC GCCGCCGATC CCGCCGGGCT
1651  CGCAGGCACC GCCGCGTATT AGCATCACCG CTCAATCGAG CACGGATTCG
1701  GCGCATGAAT CTTTTACCGC AGCTGAAGGT CCGGCCCGTC GCTGCTCTAG
1751  TGCAGATGGT CTGGATGGTC CGGCGATGGG CGCACGCACG CTGGAACTGG
1801  CTCCGGTGCC GCCGCGTGCG TCTCCGAAGC CGCCGACCCT GATTATCAAA
1851  ACGATTCCGG GTCGTGAAGA ACTGCGCTCT CTGGCGCGTC AGCGCAAATG
1901  GCGCCCGAGT ATTGGCGTGC AAGTTGAAAC CATCAGCGAC TCTGATACGG
1951  AAAACCGTTC GCGTCGCGAA TTTCACAGCA TCGGTGTCCA GGTGGAAGAA
2001  GATAAGCGTC GCGCACGTTT TAAACGCAGT AATTCCGTTA CCGCCGGCGT
2051  CCAGGCAGAC CTGGAACTGG AAGGTCTGGC AGGCCTGGCT ACCGTGGCCA
2101  CCGAAGATAA AGCGCTGCAG TTTGGTCGTT CTTTCCAACG CCATGCTAGT
2151  GAACCGCAGC CGGGTCCGCG TGCCCCGACC TACAGCGTGT TCCGTACCGT
2201  TCACACGCAG GGTCAATGGG CCTATCGCGA AGGCTACCCG CTGCCGTATG
2251  AACCGCCGGC AACCGATGGT TCTCCGGGTC CGGCCCCGGC ACCGACGCCG
2301  GGTCCGGGTG CGGGCCGTCG CGATAGTTGG ATTGAACGTG GTTCACGCTC
2351  GCTGCCGGAC TCTGGCCGTG CCAGTCCGTG CCCGCGTGAT GGTGAATGGT
2401  TTATCAAAAT GCTGCGCGCA GAAGTGGAAA AGCTGGAACA TTGGTGTCAG
2451  CAAATGGAAC GTGAAGCTGA AGATTATGAA CTGCCGGAAG AAATTCTGGA
2501  AAAAATCCGC TCCGCAGTCG GTTCAACCCA GCTGCTGCTG TCGCAAAAAG
2551  TGCAGCAATT TTTCCGTCTG TGTCAGCAAA GCATGGACCC GACCGCTTTT
2601  CCGGTTCCGA CCTTCCAGGA CCTGGCCGGT TTCTGGGATC TGCTGCAACT
2651  GAGCATTGAA GATGTCACCC TGAAATTTCT GGAACTGCAG CAACTGAAAG
2701  CCAATAGCTG GAAGCTGCTG GAACCGAAAG AAGAAAAGAA AGTGCCGCCG
2751  CCGATCCCGA AAAAGCCGCT GCGTGGTCGC GGCGTTCCGG TCAAAGAACG
2801  TTCGCTGGAC AGCGTTGATC GTCAGCGCCA AGAAGCGCGT AAACGTCTGC
2851  TGGCGGCAAA ACGTGCAGCG AGCTTTCGCC ATTCCTCAGC AACCGAATCA
2901  GCGGACTCCA TTGAAATCTA CATCCCGGAA GCCCAAACCC GTCTGTGAGC
2951  GGCCGC
```

Figure S1 | Synthetic cDNA sequence of SAPAP3 codon-optimised for E. coli expression. The 5’ end AscI and the 3’ end NotI restriction endonuclease recognition sites are highlighted in bold and italic.

a

SAPAP3 sequence identified using the *Rattus norvergicus* database

MRGYHGDRGSHPRPARFADQQHMDVGPAARAPYLLGSREAFSTEPRFCAPRAGLGHLSPEGPLSLSEGPSSV
GPEGGPGGVGAGGSSSTFPRMYPGQGPFDTCEDCVGHPQQKGATRLLPTFLDQFEKQLPVQQDGFHTLPYQR
GPAGPGPGPGSGAAPEARSESPSRIRHLVHSVQKLFAKSHSLEAPGKRDYNGPKAEGRSSSGGDSYSGPGSG
GPPTSHHHHHHHHHHHHQSRHGKRSKSKDRKGDGRHQTKATGWWSSDDNLDSDSGFLGGRPPGEPGGPFCLD
APDGSYRDLSFKGRSGGSEGRCLACTGMSMSLDGQSVKRSAWHTMMVSQGRDGYPGAGPGKGLLGPETKAKA
RTYHYLQVPQDDWGGYPTGGKDGEIPCRRMRSGSYIKAMGDEESGDSDGSPKTSPKALARRFASRRSSSVDT
ARINCCVPPRIHPRSSIPGYSRSLTTGQLSEEFNQQLEAVCGSVFGELESQAVDALDLPGCFRMRSHSYLRA
IQAGCSQDDDCLPLLAAPASVSGRPGSSFNFRKAPPPIPPGSQAPPRISITAQSSTDSAHESFTAAEGPARR
CSSADGLDGPTMGARTLELAPVPPRASPKPPTLIIKTIPGREELRSLARQRKWRPSIGVQVETISDSDTENR
SRREFHSIGVQVEEDKRRARFKRSNSVTAGVQADLELEGLAGLATVATEDKALQFGRPFQRQASEPQPGPRA
PTYSVFRTVHTQGQWAYREGYPLPYEPPATDGSPGPPPVPAPGPGSGRRDSWMERGSRSLPDSGRTSPCPRD
GEWFIKMLRAEVEKLEHWCQQMEREAEDYELPEEILEKIRSAVGSTQLLLSQKVQQFFRLCQQSLDPTAFPV
PTFQDLAGFWDLLQLSIEDVTLKFLELQQLKANSWKLLEPKEEKKVPPPIPKKPSRGRGVPVKERSLDSVDR
QRQEARKRLLAAKRAASFRHSSATESADSIEIYIPEAQTRL

b

Identification using a database with the sequence of the recombinantly-produced human SAPAP3_G^{754}-I^{968} domain

MGHHHHHHDYDDIPTTENLYFQGSPGPAPAPTPGPGAGRRDSWIERGSRSLPDSGRASPCPRDGEWFIKMLR
AEVEKLEHWCQQMEREAEDYELPEEILEKIRSAVGSTQLLLSQKVQQFFRLCQQSMDPTAFPVPTFQDLAGF
WDLLQLSIEDVTLKFLELQQLKANSWKLLEPKEEKKVPPPIPKKPLRGRGVPVKERSLDSVDRQRQEARKRL
LAAKRAASFRHSSATESADSISGNGSGGGLNDIFEAQKIEWHE

Figure S2 | Comparison of the SAPAP3 sequence identified in the interactomics approach with the sequence of the recombinant SAPAP3_G^{754}-I^{968} domain. Sequence coverage of SAPAP3 in the pull-down experiment using the *Rattus norvegicus* protein database. The underlined region corresponds to the sequence of the recombinant-SAPAP3 domain (**a**). Sequence coverage using a database containing the sequence of the recombinant protein. The double-underlined region corresponds to the sequence of the human SAPAP3_G^{754}-I^{968} domain (**b**). Colour code indicates peptides confidence: green residues correspond to peptides with 95% confidence; yellow for peptides with

confidence between 50 and 95%; red for peptides with confidence below 50%; and grey corresponds to unidentified residues.

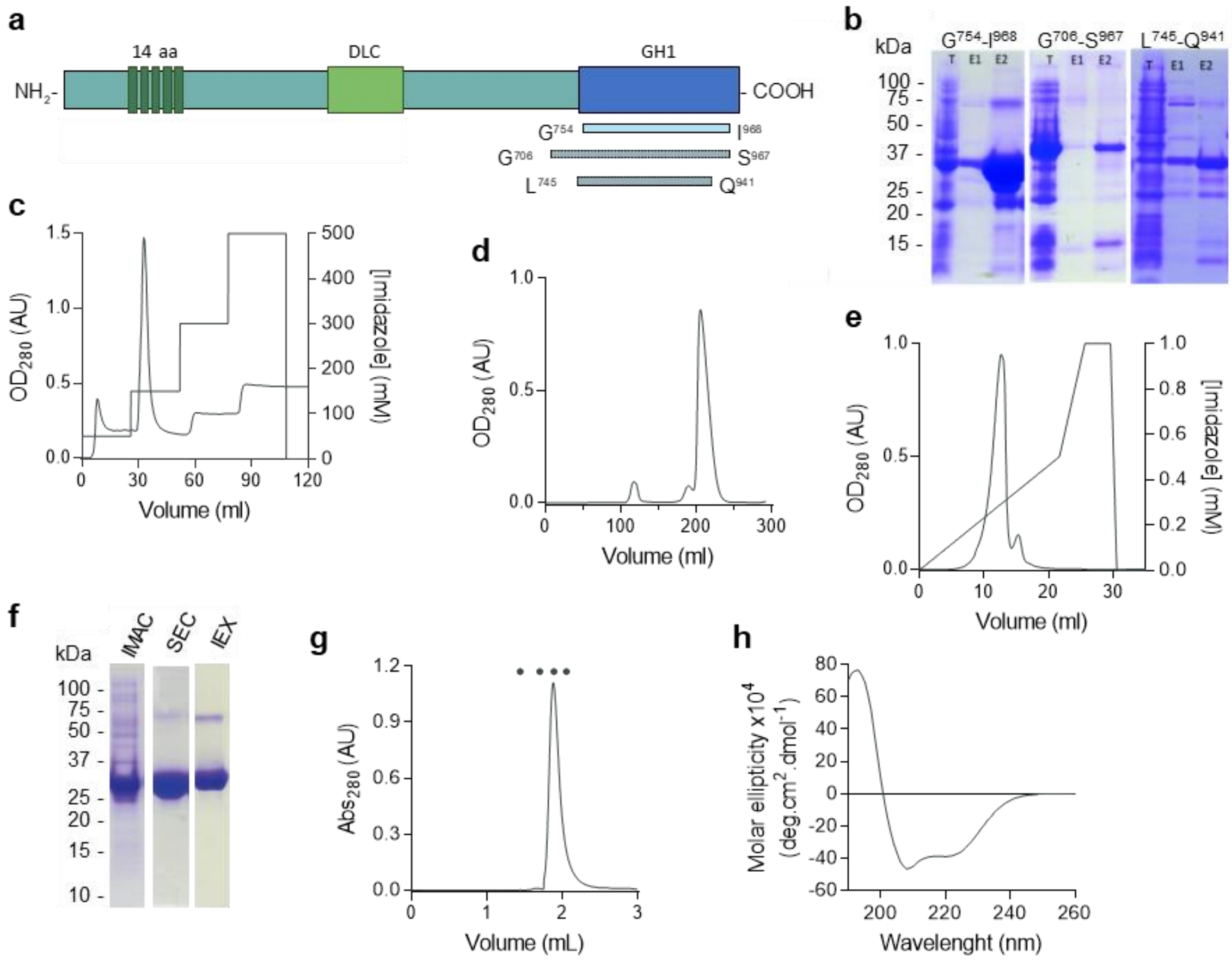

Figure S3 | SAPAP3 soluble domain determination, protein expression, and quality control of SAPAP3_G^{754}-I^{968} domain. SAPAP3 amino acid sequence reveals several domains, namely 14 aa_14 amino acid domain, DLC_dynein light chain, and an alpha-helical rich GH1_ guanylate kinase-associated protein (GKAP) homology 1 domains at the C-terminus with high homology to other members of the SAPAP protein family (e.g. **Lee et al., 2018**). SAPAP3 soluble domains boundaries obtained from ESPRIT screening (**a**). SDS-PAGE analysis of the three best expressing clones from ESPRIT screening (T - Total cell extract; E1 - IMAC elution with 100 mM imidazole; E2 - IMAC elution with 300 mM imidazole) (**b**). Chromatograms from the purification of SAPAP3_G^{754}-I^{968} domain

from 1 L expression cultures (**c**-**e**): IMAC (**c**) Preparative SEC of IMAC fractions eluted at 150 mM imidazole (**d**) and IEX chromatography of the SEC fractions eluted after 200 mL (**e**). SDS-PAGE analysis of purified SAPAP3_G^{754}-I^{968} domain (**f**). Analytical SEC of purified SAPAP3_G^{754}-I^{968} domain (**g**) - dots indicate the elution volume of column calibration proteins [aldolase (158 kDa), conalbumin (75 kDa), carbonic anhydrase (29 kDa) and ribonuclease A (13.7 kDa)], and CD spectrum of the purified SAPAP3_G^{754}-I^{968} domain (**h**).

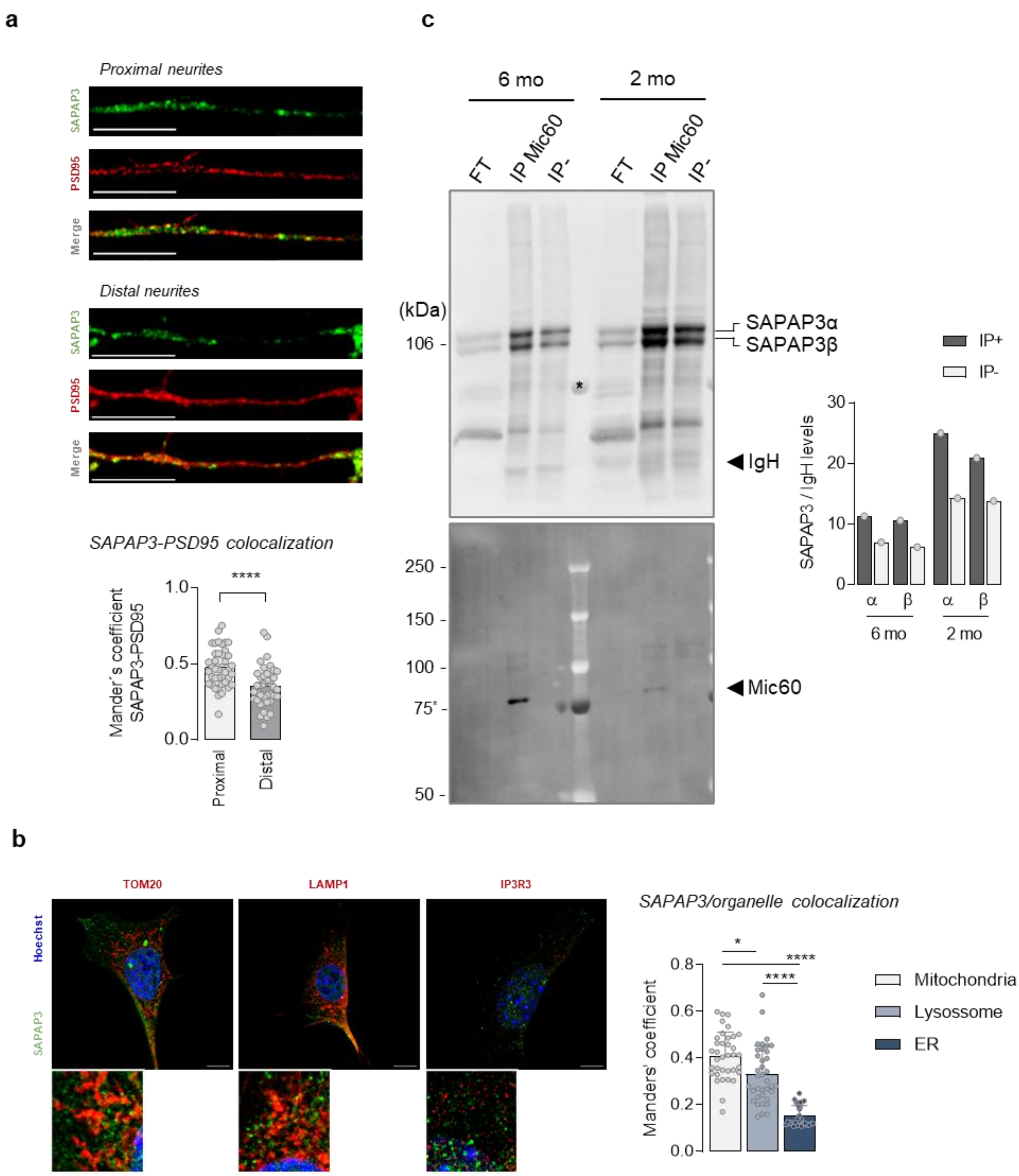

Figure S4 | Colocalization of SAPAP3 with postsynaptic density and organelle markers and SAPAP3-Mic60 immunoprecipitation. SAPAP3 co-localization with postsynaptic protein PSD95 in WT striatal mouse proximal and distal neurites and respective quantification by Manders' coefficient (**a**). Colocalization analysis between SAPAP3 and markers of mitochondria, lysosomes, and endoplasmic reticulum (ER) in striatal cells (Q7 cells); representative images of signal overlap between SAPAP3 (green) and TOM20- , LAMP1- or IP3R3 (red) and respective quantification using Manders' correlation

coefficient (**b**). Nuclei are presented in blue (Hoechst staining). Scale bar = 10 µm. Data are the mean ± SD of 3 independent experiments, considering 10-15 cells *per* condition. Statistical analysis: *$p<0.05$ and ****$p<0.0001$ by Kruskal-Wallis's followed by uncorrected Dunn's test. Endogenous Mic60 was immunopurified (IP Mic60) from striatal samples of WT mice at 2 and 6 months of age using a rabbit polyclonal anti-Mic60 antibody. Equivalent amounts of total protein were subjected to a negative control immunoprecipitation (IP-) using a normal rabbit IgG to assess nonspecific interactions. Western blotting analysis using a SAPAP3 antibody revealed both the α and β isoforms, as well as the retained antibody heavy chain (IgH), which served as a control to confirm equal antibody amounts in the IP Mic60 and IP- samples. * in the upper membrane indicates the 75 kDa molecular weight marker. The SAPAP3/IgH values are presented in the graph for both α- and β- SAPAP3 subunits for samples at 6 and 2 months of age (mo). (FT): Flow-through fraction (unbound fraction) **(c)**.

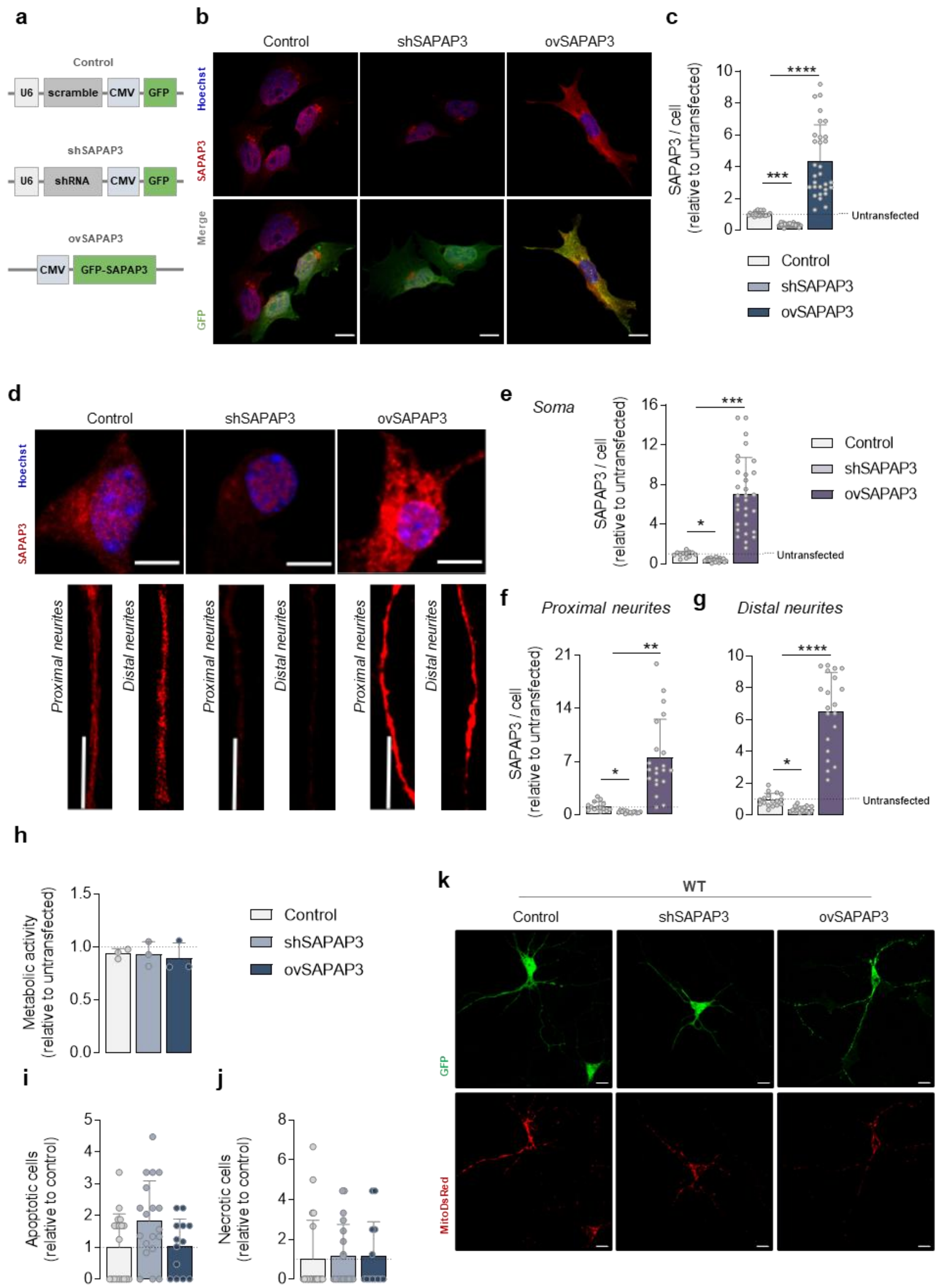

Figure S5 | Striatal cells modulation of SAPAP3 levels, cell viability and neuronal morphology. Schematic representation of the GFP-control, -shSAPAP3 and -ovSAPAP3 plasmid constructs (**a**). Striatal cells (Q7 cells and WT neurons) were transfected with

either GFP-control, -shSAPAP3 or -ovSAPAP3 plasmids. Representative images of SAPAP3 levels (red) in Q7 transfected cells (green, GFP signal) (**b**) and respective quantification (**c**). Representative images of SAPAP3 levels (red) in WT transfected neurons (green, GFP signal) (**d**) and quantification at soma (**e**) and proximal (**f**) and distal (**g**) neurites. Proximal neurites were defined within 50 µm of the soma (< 50 µm), whereas distal neurites were analyzed at distances greater than 50 µm from the soma (> 50 µm). Nuclei are presented in blue (Hoechst staining). Scale bar = 10 µm. Data are the mean ± SD of 3-4 independent experiments, considering 5-8 cells per condition. Analysis of cell metabolic activity (**h**) and quantification of apoptotic (**i**) and necrotic (**j**) in Q7 striatal cells transfected with GFP-control, -shSAPAP3 or -ovSAPAP3 plasmids. Data are the mean ± SD of 3 independent experiments performed in quadruplicates for metabolic activity (**h**) and of 13-21 cells, randomly selected across three independent replicates per condition (**i** and **j**). WT striatal neurons were transfected with either GFP-control, -shSAPAP3 or -ovSAPAP3 plasmids plus MitoDsRed and low-magnification immunocytochemistry images obtained to show the overall cellular morphology of neurites shown in Fig. 2j (**k**). Statistical analysis: **p < 0.01 ***p < 0.001 and ****p < 0.0001 by Kruskal-Wallis test followed by uncorrected Dunn's test.

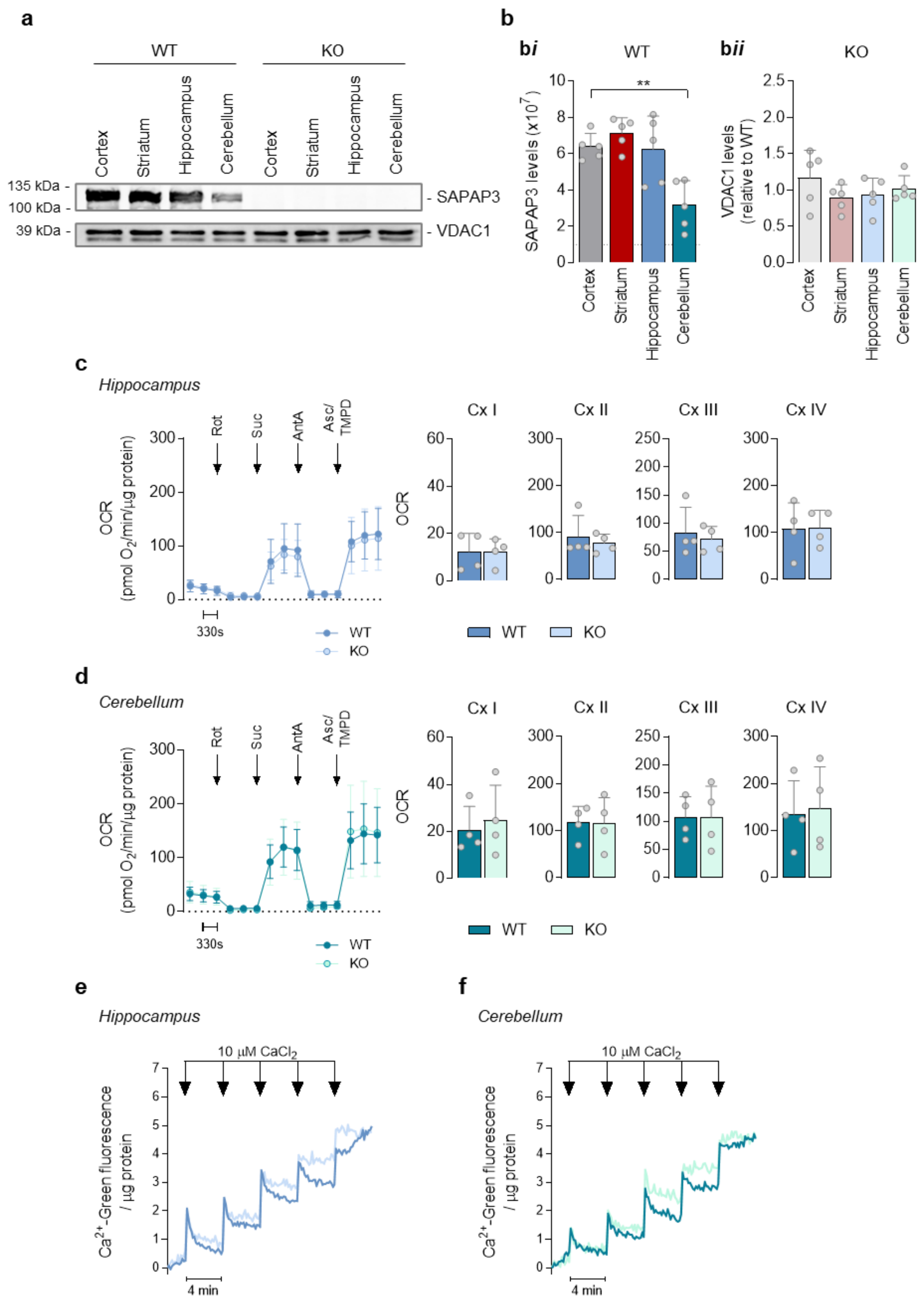

Figure S6 | SAPAP3 levels, activity of respiratory chain complexes and Ca^{2+}-handling in SAPAP3-KO vs WT mouse brain mitochondria. Mitochondrial fractions prepared from cortex, striatum, hippocampus and cerebellum of WT or SAPAP3-KO mouse brain were

probed with the affinity-purified SAPAP3 antibody that recognizes two major bands of approximately 110 and 130 kDa. VDAC1 was used as the loading control (**a**). Densitometric analysis of SAPAP3 levels in WT mice (**bi**). Densitometric analysis of VDAC1 levels in SAPAP3-KO mice relative to WT (**bii**). The activity of mitochondrial respiratory chain complexes Cx I-IV was evaluated in 3-month-old SAPAP3-KO and WT mice isolated hippocampal (**c**) and cerebellar (**d**) mitochondria following sequential additions of rotenone (2 µM), succinate (10 mM), antimycin A (4 µM) and ascorbate (10 mM) plus TMPD (100 µM). Mitochondria obtained from the hippocampus (**e**) and cerebellum (**f**) were also analysed for their capacity to handle Ca^{2+} after five pulses of 10 µM Ca^{2+} (arrows). Data are the mean ± SD of independent mitochondrial preparations derived from 4-5 mice. Statistical analysis: **$p<0.01$ by one-way ANOVA followed by uncorrected Fisher's LSD test.

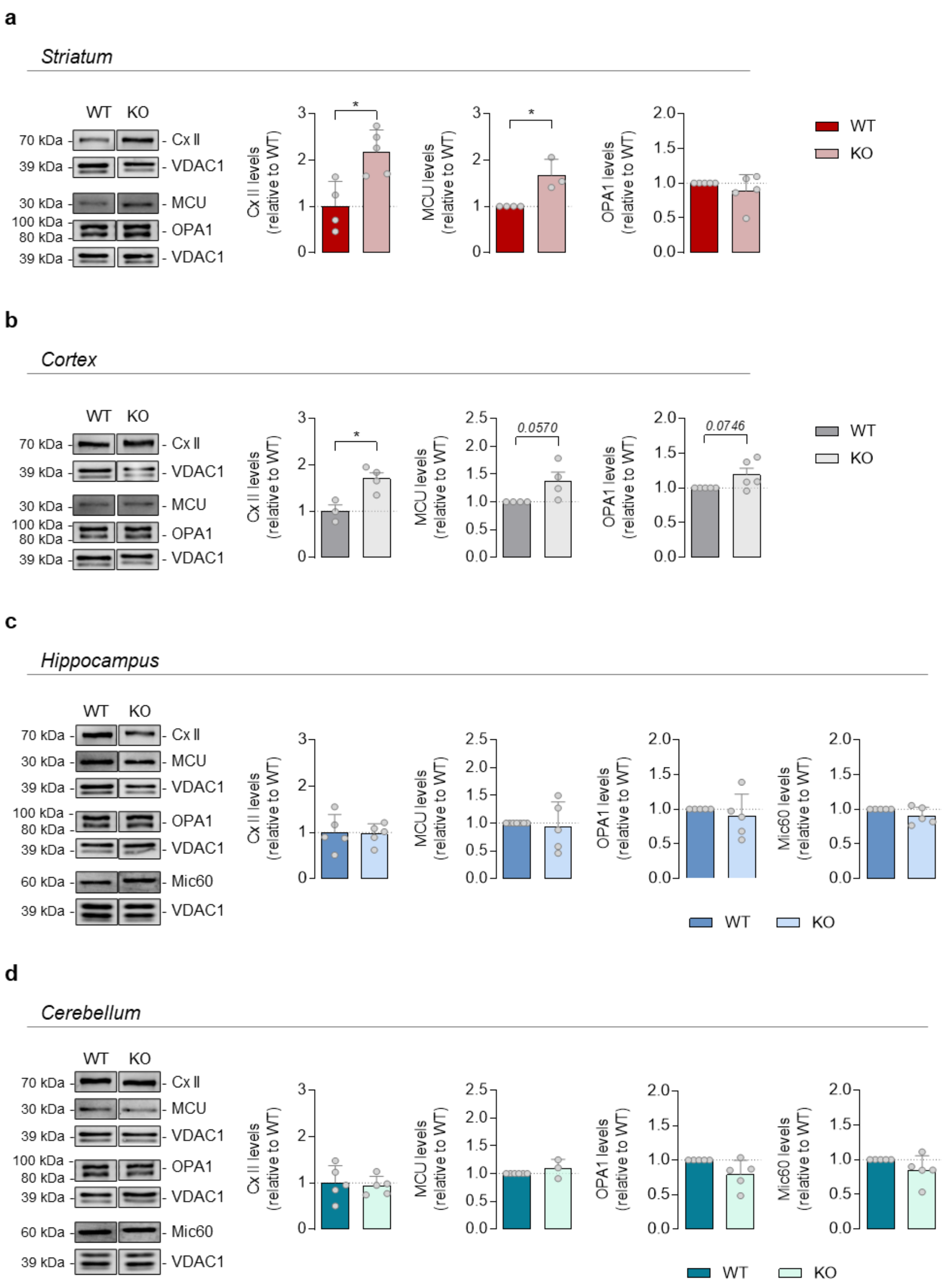

Figure S7 | Protein levels of Cx-II subunit, MCU, OPA1 and Mic60 in SAPAP3-KO and WT brain mitochondria. Levels of 70 kDa-Cx II subunit, MCU, Mic60 and OPA1 in isolated mitochondria from striatum (**a**), cortex (**b**), hippocampus (**c**) and cerebellum (**d**) were analysed by western blotting. VDAC1 was used as loading control. Data are the mean ±

SD of independent mitochondrial preparations derived from 5 mice. Statistical analysis: *p<0.05 by Mann Whitney test.

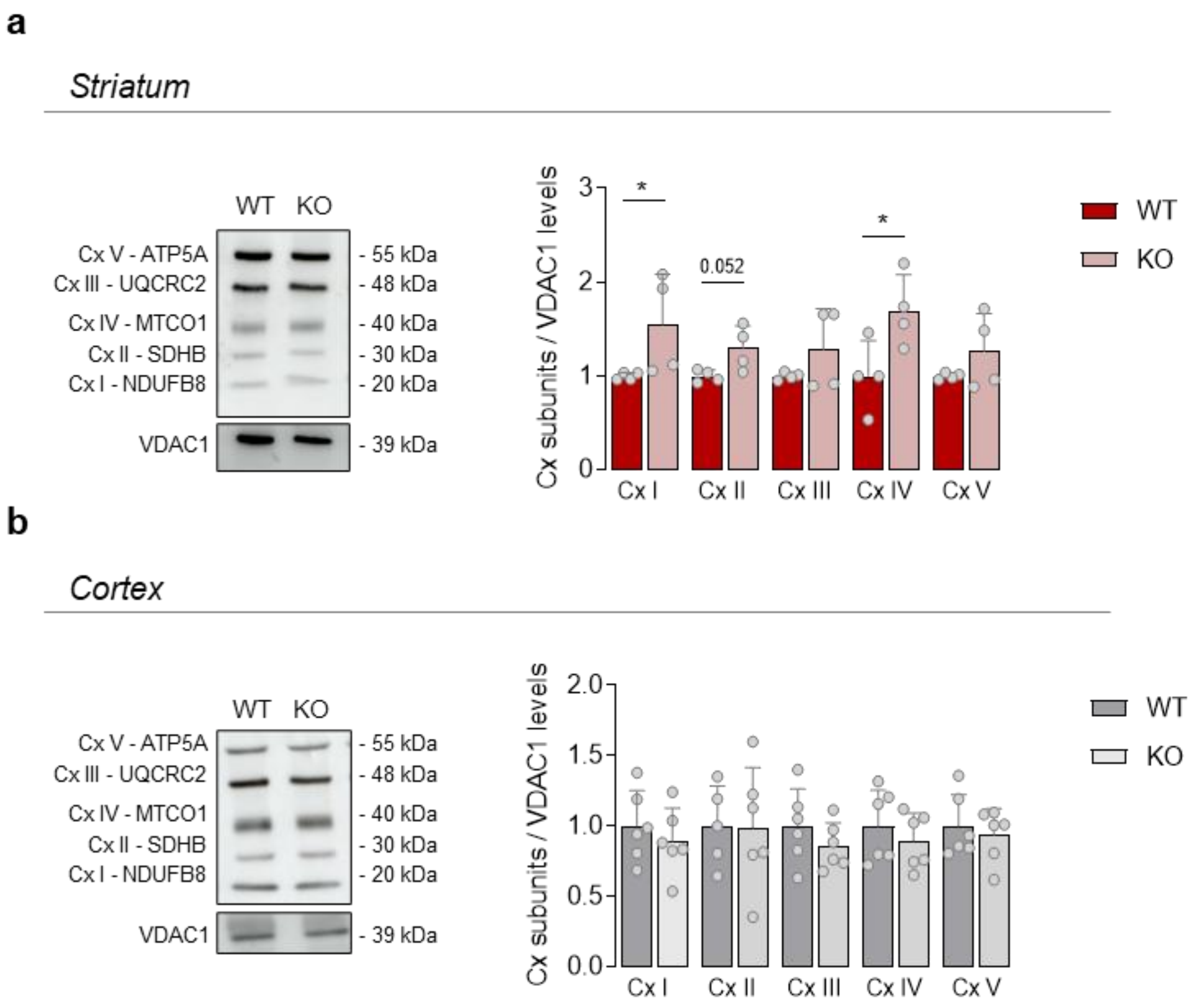

Figure S8 | Region-specific regulation of mitochondrial OXPHOS protein levels in SAPAP3-KO mice. Mitochondrial fractions prepared from striatum (**a**) or cortex (**b**) of WT or SAPAP3-KO mice were probed with the affinity-purified total OXPHOS antibody, as described in methods section, representing the five respiratory chain complexes, of approximately 20 kDa (CI-NDUFB8), 30 kDa (CII-SDHB), 40 kDa (CIV-MTCO1), 48 kDa (CIII-UQCRC2) and 55 kDa (CV-ATP5A). VDAC1 was used as the loading control. Data are the mean ± SD of independent mitochondrial preparations derived from 4-6 mice. Statistical analysis: *p<0.05 by unpaired Student's t-test with Welch's correction.

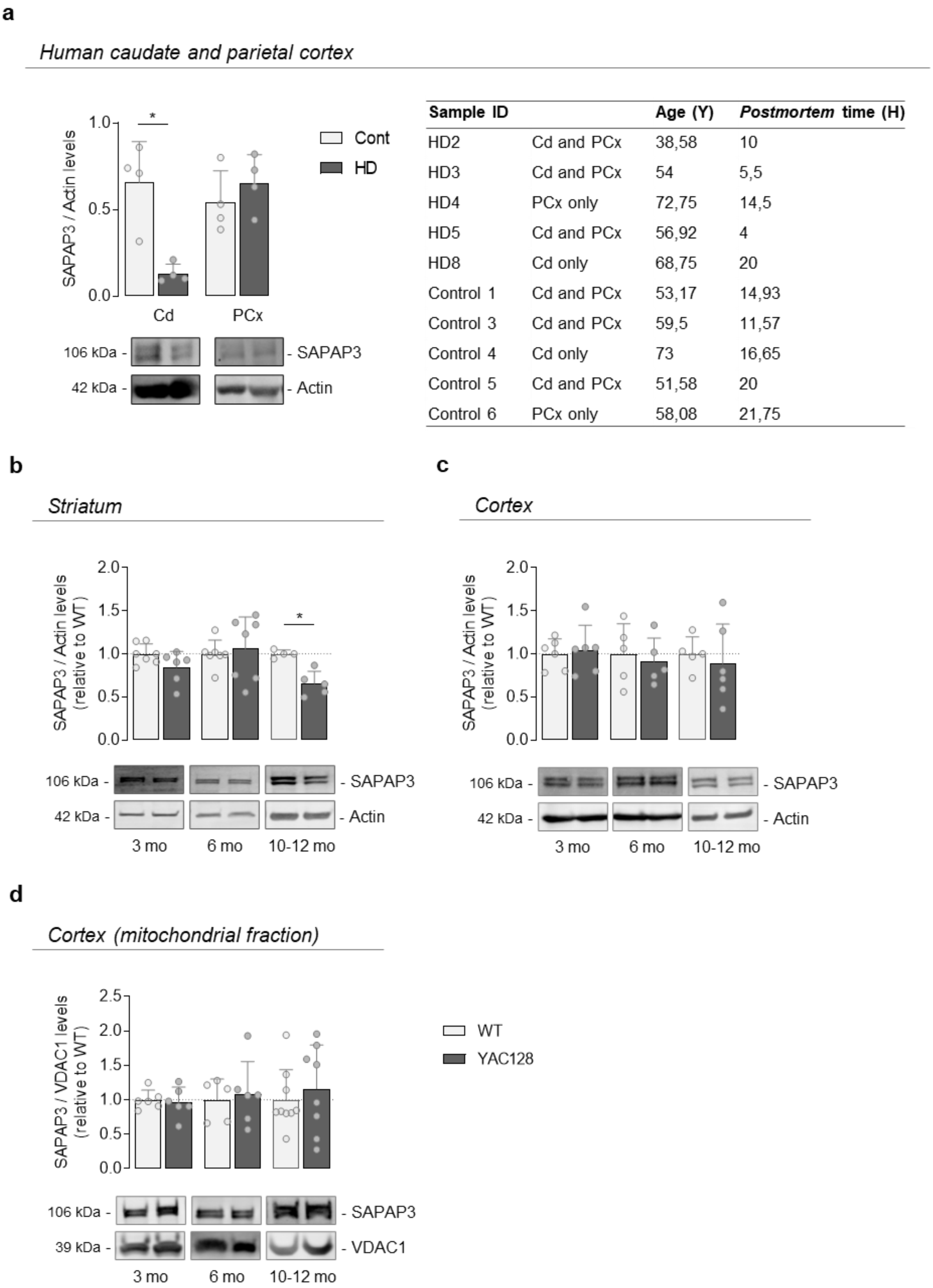

**Sample ID**		**Age (Y)**	***Postmortem* time (H)**
HD2	Cd and PCx	38,58	10
HD3	Cd and PCx	54	5,5
HD4	PCx only	72,75	14,5
HD5	Cd and PCx	56,92	4
HD8	Cd only	68,75	20
Control 1	Cd and PCx	53,17	14,93
Control 3	Cd and PCx	59,5	11,57
Control 4	Cd only	73	16,65
Control 5	Cd and PCx	51,58	20
Control 6	PCx only	58,08	21,75

Figure S9 | SAPAP3 total protein levels in HD postmortem human caudate and parietal cortex and in YAC128 striatum and cortex, and cortical mitochondria. Total fractions

prepared from human caudate (Cd) and parietal cortex (PCx) identified for age at death and *postmortem* time for sample collection of control subjects (Cont) and HD patients (**a**), WT or YAC128 mouse total striatum (**b**) and cortex (**c**) and isolated mmitochondrial fractions from WT or YAC128 mouse cortex (**d**) were probed with the affinity-purified SAPAP3 antibody, using actin or VDAC1 as the loading control. Densitometric analysis of SAPAP3 in YAC128 mice relative to WT at 3, 6 and 10-12 months. Data are the mean ± SD of independent preparations derived from 4 human samples or 5-9 mice.

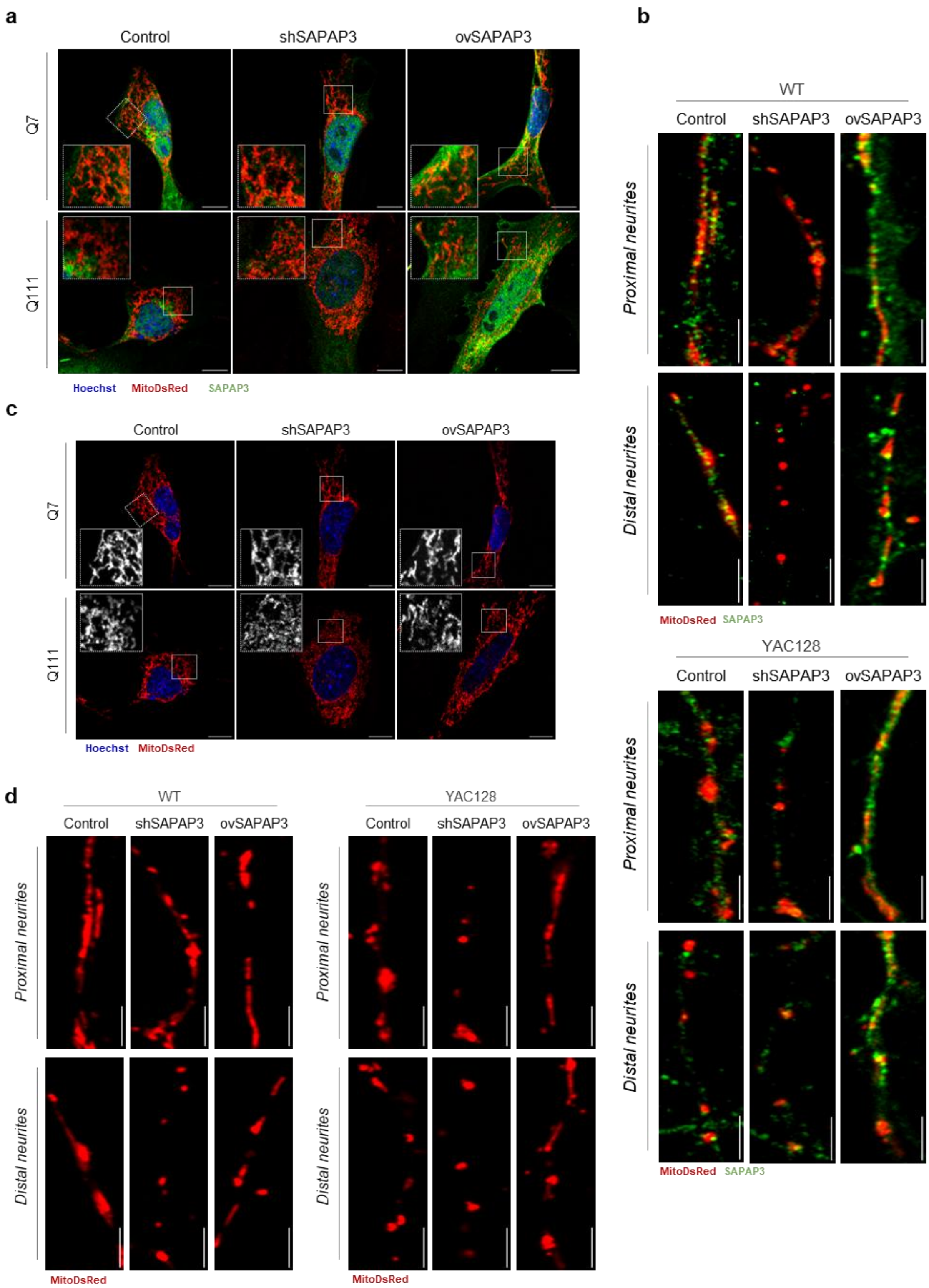

Figure S10 | Representative images of SAPAP3 and mitochondrial labeling in HD striatal cells and neurons subjected to modulation of SAPAP3.

Mouse striatal cells (Q7 *and* Q111) (**a, c**) and primary neurons (WT *and* YAC128) (**b,d**) were transfected with either control-GFP, shSAPAP3 or ovSAPAP3, or MitoDsRed to label mitochondria, and immunocytochemistry was performed to label SAPAP3. Representative images show MitoDsRed in red, SAPAP3 in green and nuclei in blue (Hoechst staining) (**a,b**). Representative MitoDsRed labeling is shown to allow the visualization of mitochondrial morphology (**c,d**). Proximal neurites were defined within 50 µm of the soma (< 50 µm), whereas distal neurites were analyzed at distances greater than 50 µm from the soma (> 50 µm) (**b,d**). Quantification of SAPAP3 co-localizing with mitochondria and mitochondrial morphology phenotypes are detailed in Figure 4. Scale bar = 10 µm (**a,c**); 5 µm (**b,d**).

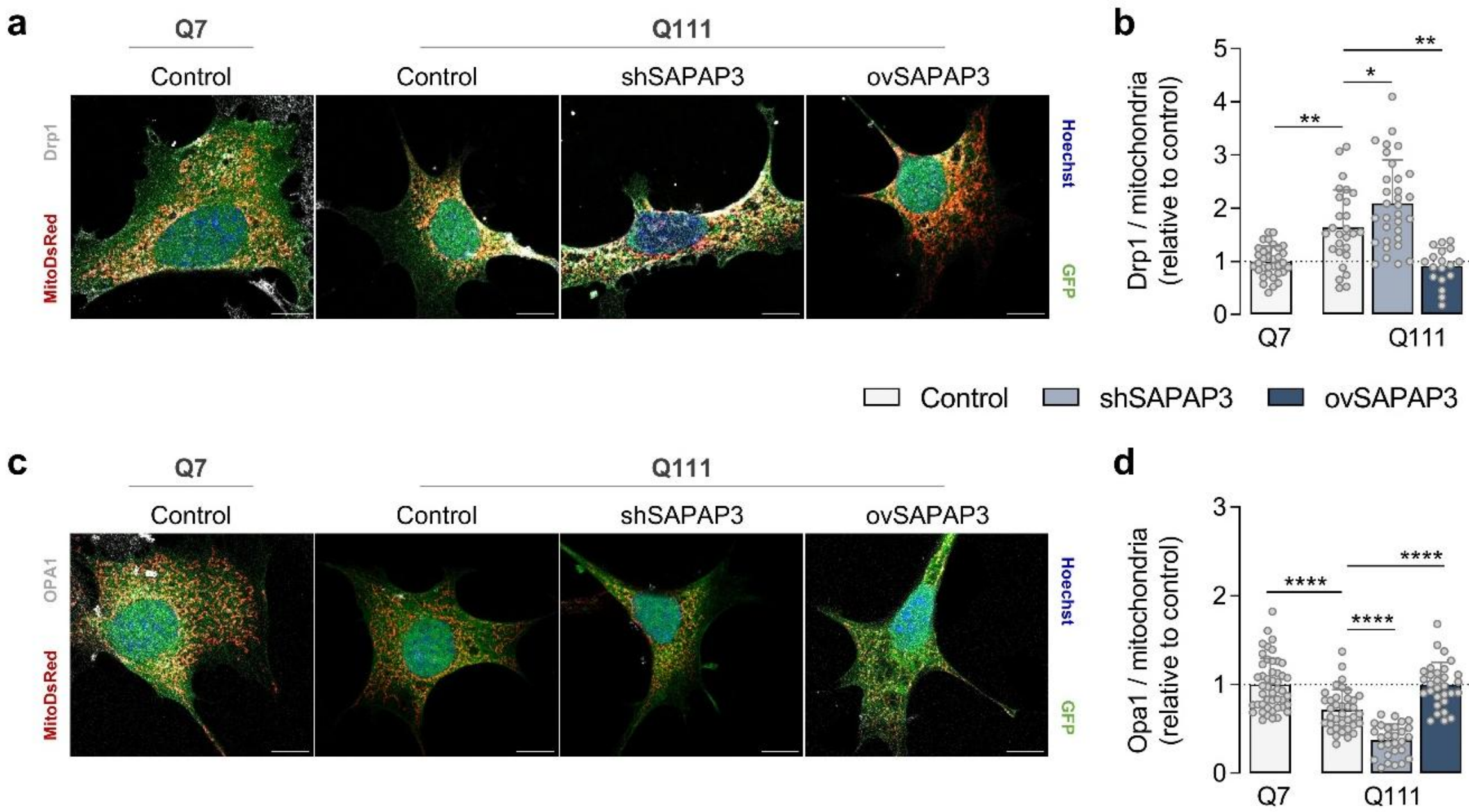

Figure S11 | Drp1 and OPA1 levels within mitochondria in striatal cells. Striatal Q7 and Q111 cells were transfected with either GFP-control, -shSAPAP3 or -ovSAPAP3 plasmids. Representative images of Drp1 (grey) within mitochondria (red, labelled by MitoDsRed)

in in GFP-transfected cells (green) (**a**) and quantification (**b**). Representative images of OPA1 (grey) within mitochondria (red, labelled by MitoDsRed) in in GFP-transfected cells (green) (**c**) and quantification (**d**). Nuclei are presented in blue (Hoechst staining). Scale bar = 10 µm. Data are the mean ± SD of 3-4 independent experiments, considering ~8 cells per condition. Statistical analysis: *$p < 0.05$ and ****$p < 0.0001$ by two-way ANOVA followed by Tukey's multiple comparison test.

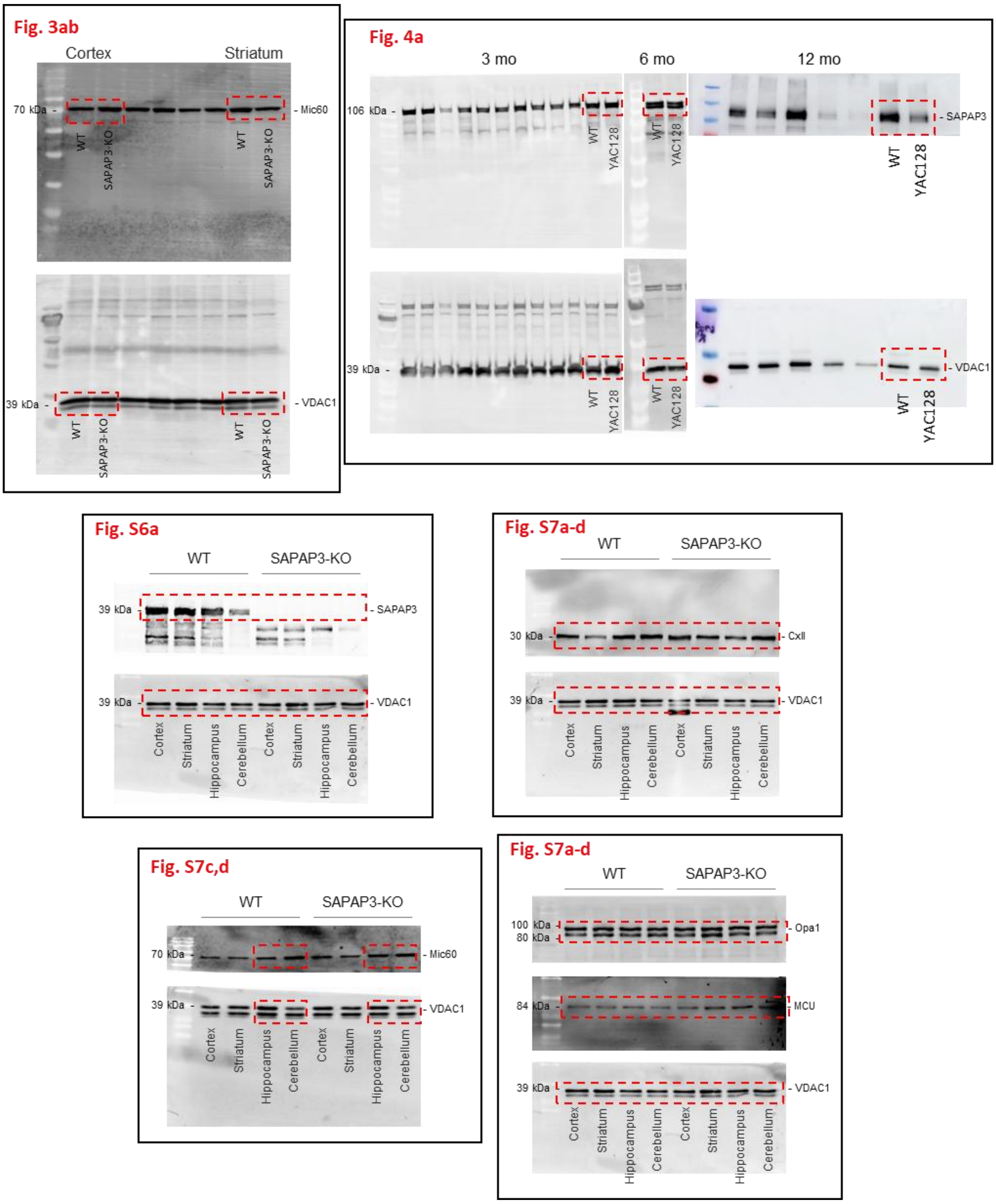

Figure S12 | Full blots. Red boxes regions represent the portion used in representative Western blot images for Figure 3a,b, Figure 4a, Figure 6a, Figure S7a-d, Figure S7c,d and Figure S7a-d.

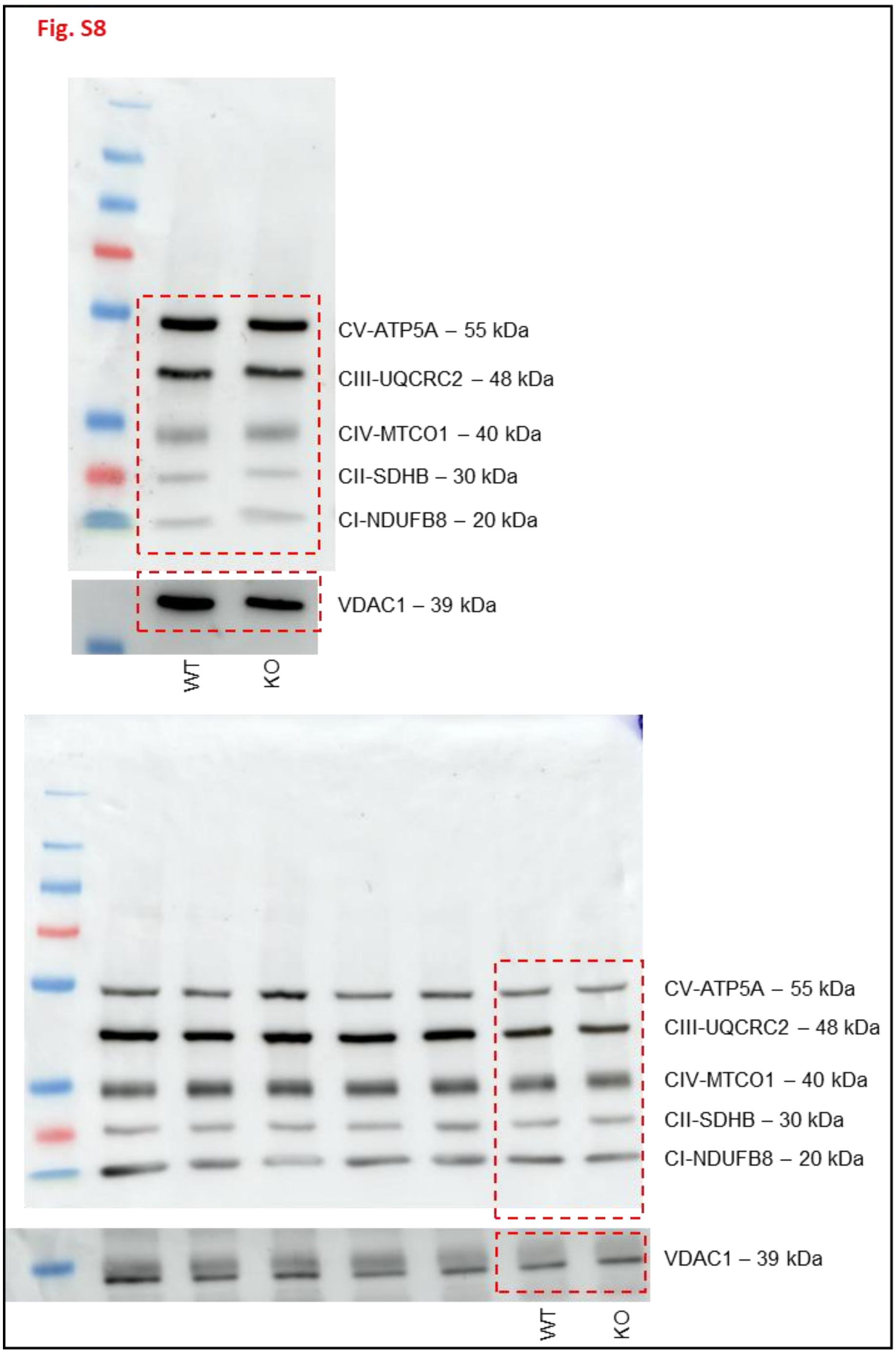

Figure S13 | Full blots. Red boxes regions represent the portion used in representative Western blot images for Figure S8.

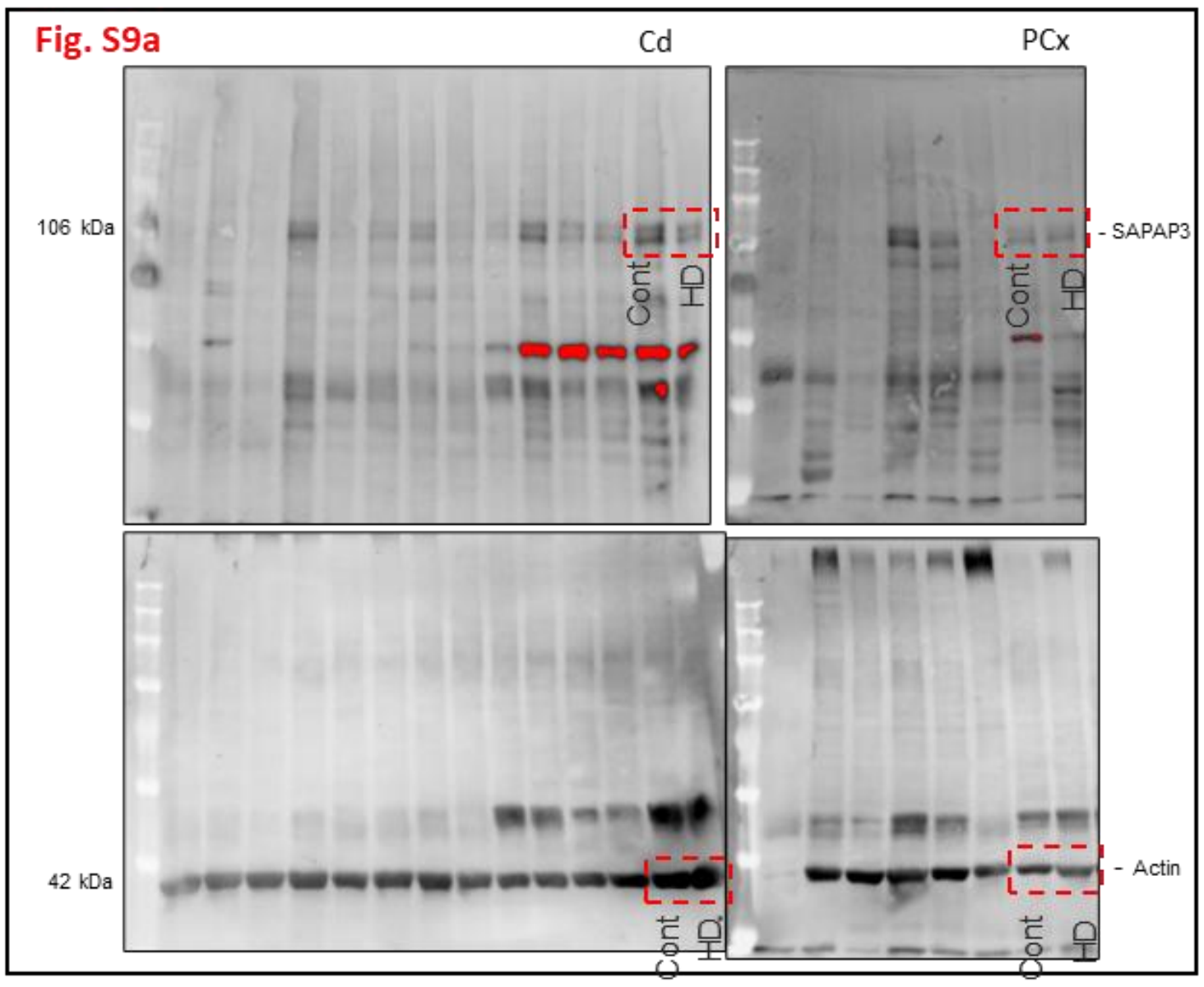

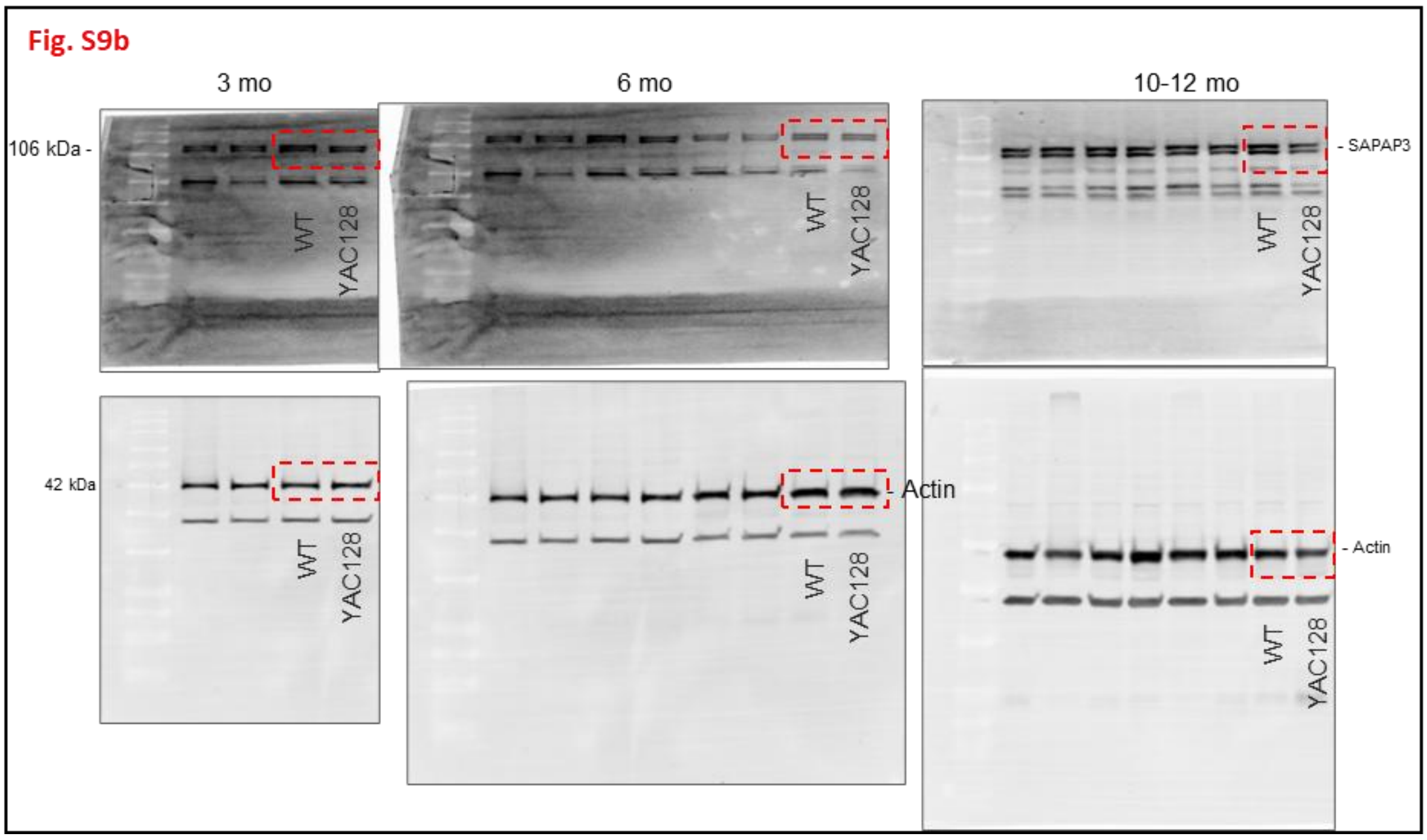

Figure S14 | Full blots. Red boxes regions represent the portion used in representative Western blot images for Figure S9a and Figure S9b.

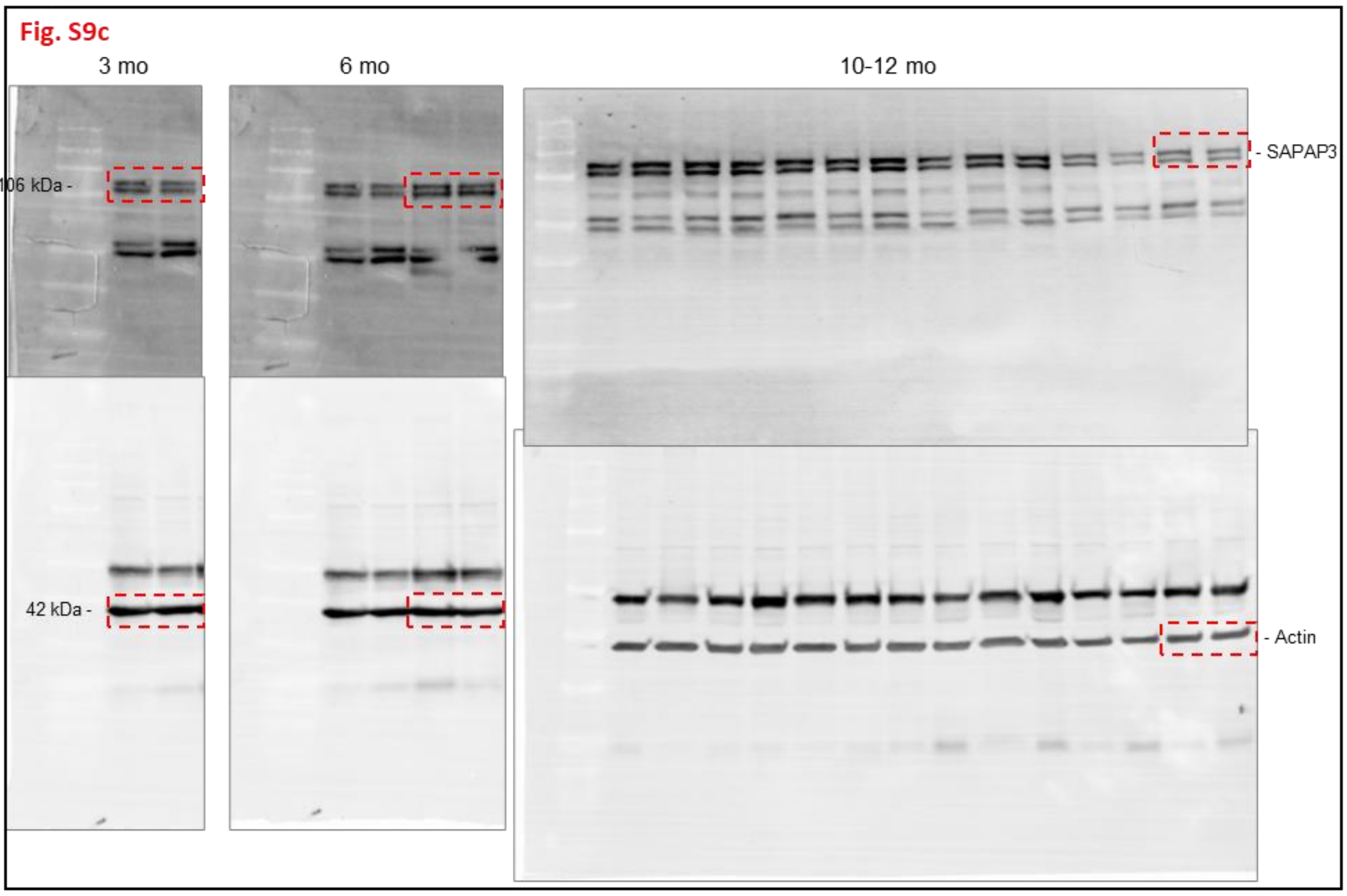

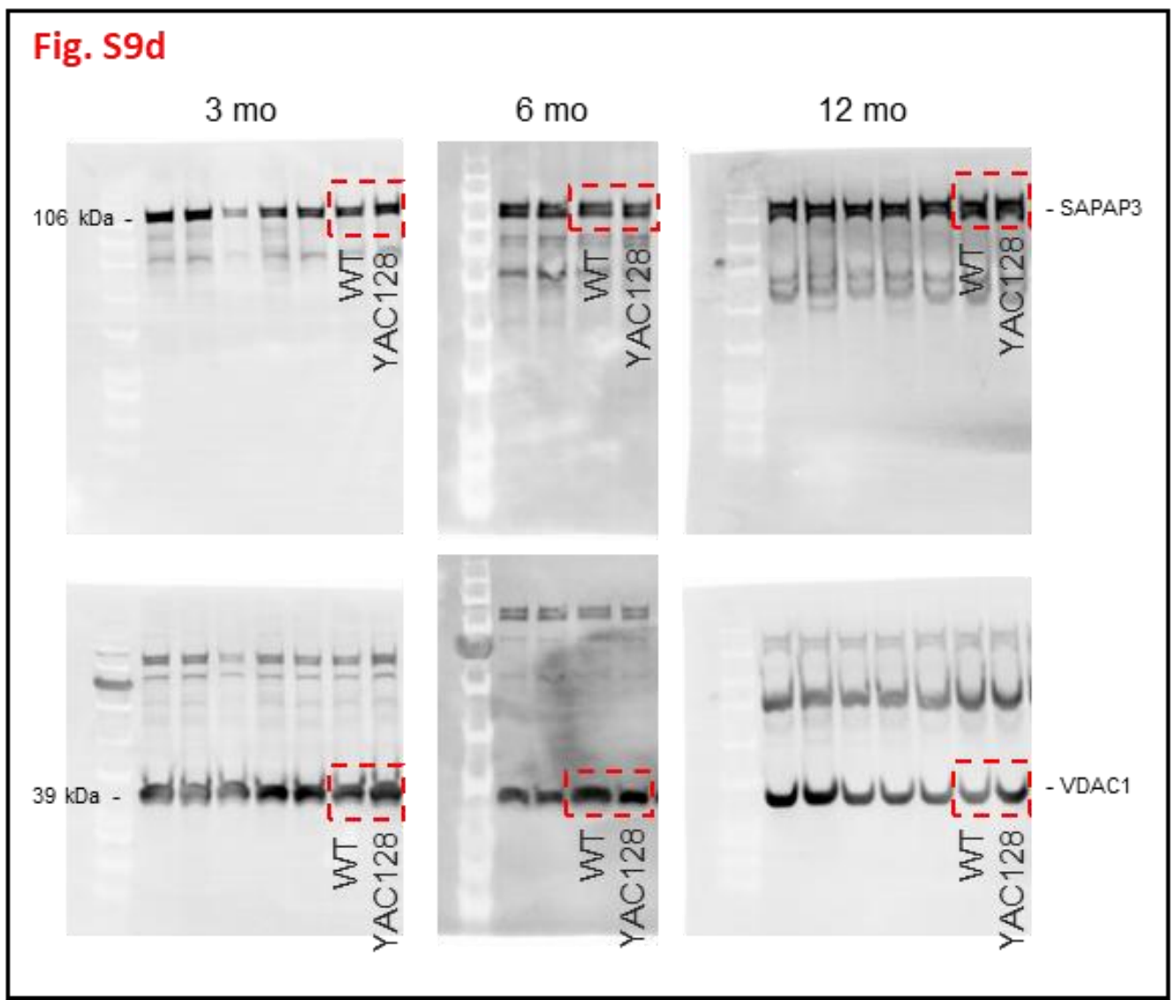

Figure S15 | Full blots. Red boxes regions represent the portion used in representative Western blot images for Figure S9c and Figure S9d.

Table S1 | Summary of the potential synaptoneurosomal-interactors of SAPAP3_ G^{754}-I^{968} domain, detected by SWATH-MS (proteins highlighted in the Volcano plot - Fig. 1)

UNIPROT accession number	Gene name	Protein names	Difference (SAPAP3-Strep)	Two-tailed Student's t-test	
				-Log_{10} (p-value)	q-value*
**Q3KR86**	*Immt Mic60*	MICOS complex subunit Mic60	3.4	6.54	0.00
**Q68FY0**	*Uqcrc1*	Cytochrome b-c1 complex subunit 1, mitochondrial	3.0	5.35	0.00
**P97838**	*Dlgap3 Dap3*	Disks large-associated protein 3	2.5	5.68	0.00
**Q63505**	*Gtf3c1*	General transcription factor 3C polypeptide 1	6.6	4.02	0.00
**P97849**	*Slc27a1 Fatp Fatp1*	Long-chain fatty acid transport protein 1	1.9	2.11	0.00
**Q5XIF6**	*Tuba4a Tuba4*	Tubulin alpha-4A chain	2.2	2.16	0.00
**P10888**	*Cox4i1 Cox4 Cox4a*	Cytochrome c oxidase subunit 4 isoform 1, mitochondrial	0.9	3.05	0.00
**P69897**	*Tubb5*	Tubulin beta-5 chain	1.5	2.57	0.00
**Q3KRE0**	*Atad3 Atad3a*	ATPase family AAA domain-containing protein 3	1.8	2.57	0.00
**Q1AAU6**	*Asap1 Ddef1*	Arf-GAP with SH3 domain, ANK repeat and PH domain-containing protein 1	2.0	2.56	0.00
**P81155**	*Vdac2*	Voltage-dependent anion-selective channel protein 2	1.9	2.77	0.00
**O35796**	*C1qbp Gc1qbp*	Complement component 1 Q subcomponent-binding protein, mitochondrial	1.9	2.76	0.00
**Q66HF8**	*Aldh1b1 Aldhx*	Aldehyde dehydrogenase X, mitochondrial	2.5	2.62	0.00
**P09606**	*Glul Glns*	Glutamine synthetase	3.1	2.84	0.01
**P63045**	*Vamp2 Syb2*	Vesicle-associated membrane protein 2	1.9	3.68	0.01
**P32551**	*Uqcrc2*	Cytochrome b-c1 complex subunit 2, mitochondrial	2.4	3.47	0.01
**Q7TSA0**	*Rhot2 Arht2*	Mitochondrial Rho GTPase 2	2.5	3.49	0.01
**P32851**	*Stx1a Sap*	Syntaxin-1A	2.3	3.79	0.01
**P21396**	*Maoa*	Amine oxidase [flavin-containing] A	2.0	3.97	0.01
**P60881**	*Snap25 Snap*	Synaptosomal-associated protein 25	2.8	4.13	0.01
**Q6P9T8**	*Tubb4b Tubb2c*	Tubulin beta-4B chain	2.2	1.88	0.01
**Q4QRB4**	*Tubb3*	Tubulin beta-3 chain	1.5	2.02	0.01
**Q6AXV4**	*Samm50*	Sorting and assembly machinery component 50 homolog	2.8	4.29	0.01
**Q5RJY4**	*Dhrs7b Sdr32c1*	Dehydrogenase/reductase SDR family member 7B	1.2	2.12	0.01
**P61265**	*Stx1b Stx1b2*	Syntaxin-1B	2.0	1.80	0.01
**P12075**	*Cox5b*	Cytochrome c oxidase subunit 5B, mitochondrial	2.2	1.67	0.01
**P21707**	*Syt1*	Synaptotagmin-1	0.9	1.97	0.02
**Q64428**	*Hadha*	Trifunctional enzyme subunit alpha, mitochondrial	1.3	1.73	0.02
**P11951**	*Cox6c2 Cox6c*	Cytochrome c oxidase subunit 6C-2	1.3	1.64	0.02
**Q9R1Z0**	*Vdac3*	Voltage-dependent anion-selective channel protein 3	0.9	1.87	0.02

*The q-value corresponds to the p-value corrected for multiple comparisons using a 5% FDR.

Table S2 | SWATH Variable Window

	m/z range	*Width (Da)*
**Window 1**	349.5-363.1	13.6
**Window 2**	362.1-378.4	16.3
**Window 3**	377.4-392.8	15.4
**Window 4**	391.8-405.4	13.6
**Window 5**	404.4-417.1	12.7
**Window 6**	416.1-427.4	11.3
**Window 7**	426.4-437.3	10.9
**Window 8**	436.3-446.8	10.5
**Window 9**	445.8-456.2	10.4
**Window 10**	455.2-465.2	10
**Window 11**	464.2-474.2	10
**Window 12**	473.2-482.8	9.6
**Window 13**	481.8-490.4	8.6
**Window 14**	489.4-498.1	8.7
**Window 15**	497.1-505.3	8.2
**Window 16**	504.3-512.5	8.2
**Window 17**	511.5-519.7	8.2
**Window 18**	518.7-526.4	7.7
**Window 19**	525.4-533.2	7.8
**Window 20**	532.2-540.4	8.2
**Window 21**	539.4-547.1	7.7
**Window 22**	546.1-554.4	8.3
**Window 23**	553.4-561.6	8.2
**Window 24**	560.6-568.8	8.2
**Window 25**	567.8-576.4	8.6
**Window 26**	575.4-584.1	8.7
**Window 27**	583.1-591.7	8.6
**Window 28**	590.7-599.4	8.7
**Window 29**	598.4-607	8.6
**Window 30**	606-614.7	8.7
**Window 31**	613.7-622.8	9.1
**Window 32**	621.8-631.3	9.5
**Window 33**	630.3-639.4	9.1
**Window 34**	638.4-647.5	9.1
**Window 35**	646.5-655.6	9.1
**Window 36**	654.6-663.7	9.1
**Window 37**	662.7-672.7	10
**Window 38**	671.7-681.7	10
**Window 39**	680.7-690.7	10
**Window 40**	689.7-700.2	10.5
**Window 41**	699.2-709.6	10.4
**Window 42**	708.6-719.5	10.9
**Window 43**	718.5-729	10.5
**Window 44**	728-738.4	10.4
**Window 45**	737.4-748.3	10.9
**Window 46**	747.3-758.2	10.9
**Window 47**	757.2-768.6	11.4
**Window 48**	767.6-780.3	12.7
**Window 49**	779.3-792.4	13.1
**Window 50**	791.4-806.8	15.4
**Window 51**	805.8-822.1	16.3
**Window 52**	821.1-838.8	17.7
**Window 53**	837.8-858.1	20.3
**Window 54**	857.1-879.3	22.2
**Window 55**	878.3-903.1	24.8
**Window 56**	902.1-931.9	29.8
**Window 57**	930.9-968.8	37.9
**Window 58**	967.8-1016.5	48.7
**Window 59**	1015.5-1091.2	75.7
**Window 60**	1090.2-1249.6	159.4
