# Supplementary material for: The postsynaptic scaffolding protein SAPAP3 shapes mitochondrial activity: the case of Huntington’s disease
Source: Cell Death Dis. 2026 Aug 7;17(1):936. doi: 10.1038/s41419-026-09156-4 (PMC13731651; doi:10.1038/s41419-026-09156-4)
Supplement: Supplementary file 2 [file 41419_2026_9156_MOESM2_ESM.pdf]

Fig. 3ab

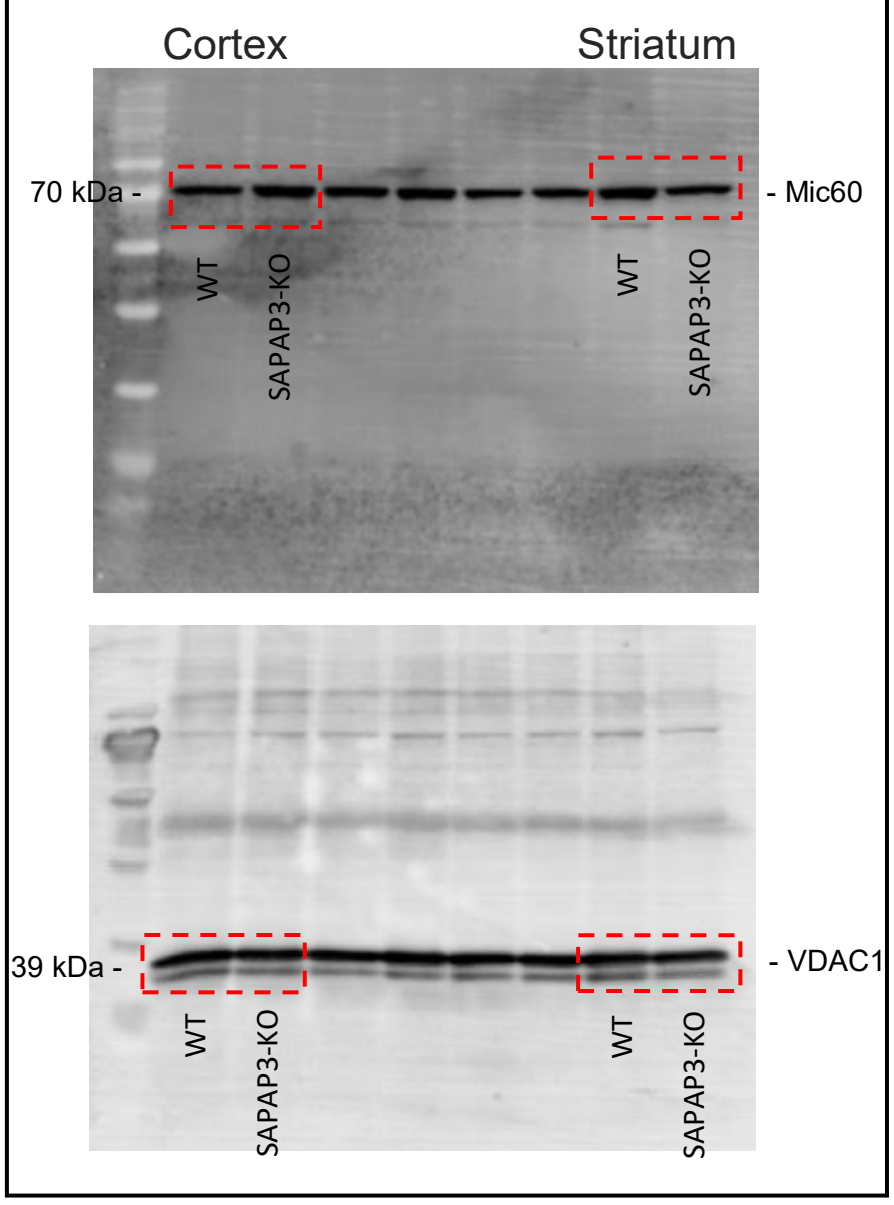

Fig. 4a

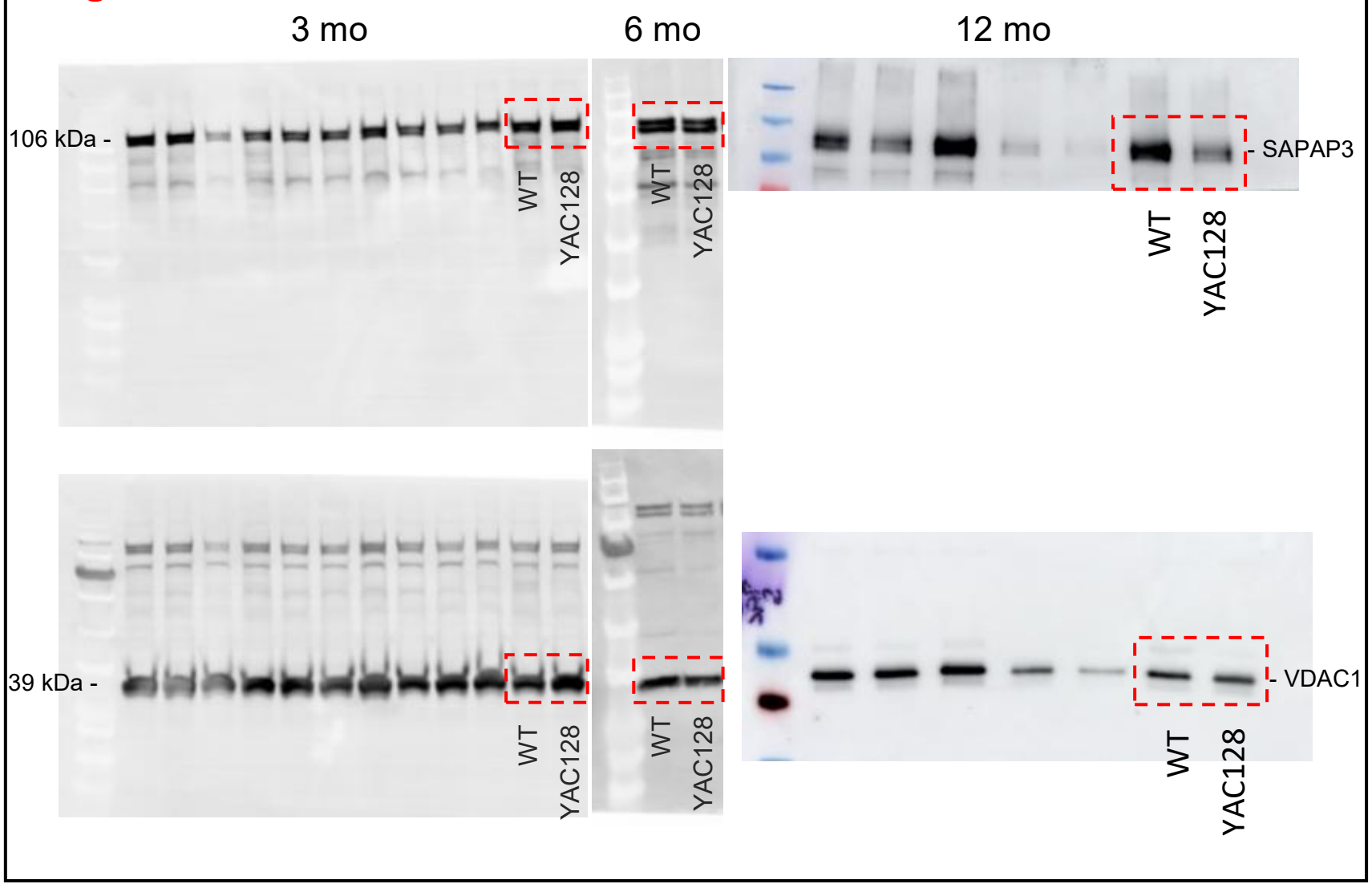

Fig. S6a

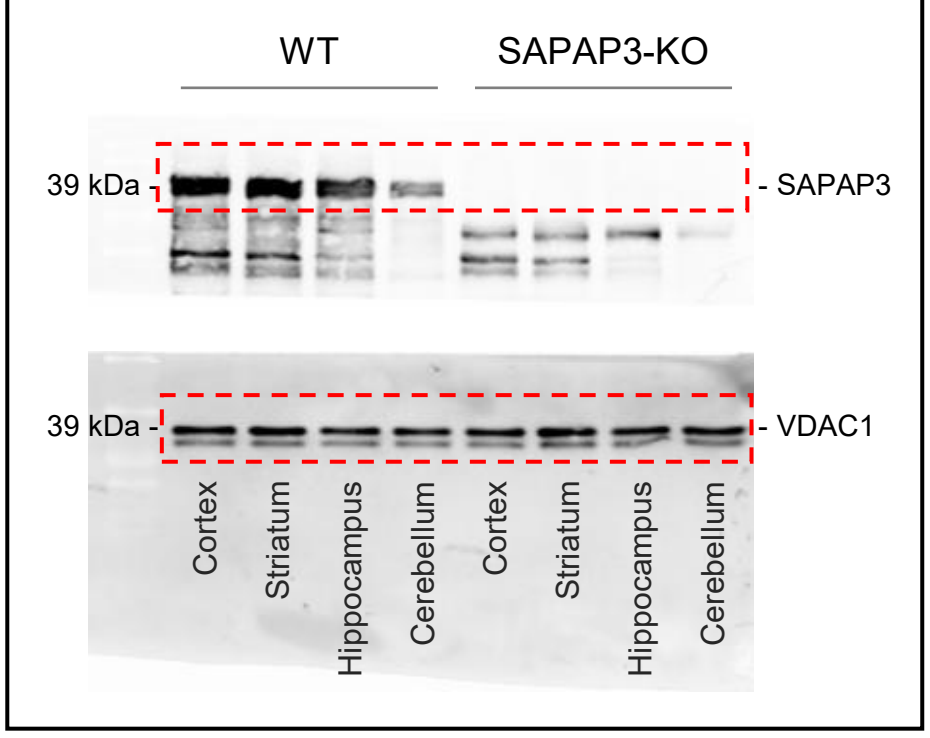

Fig. S7a-d

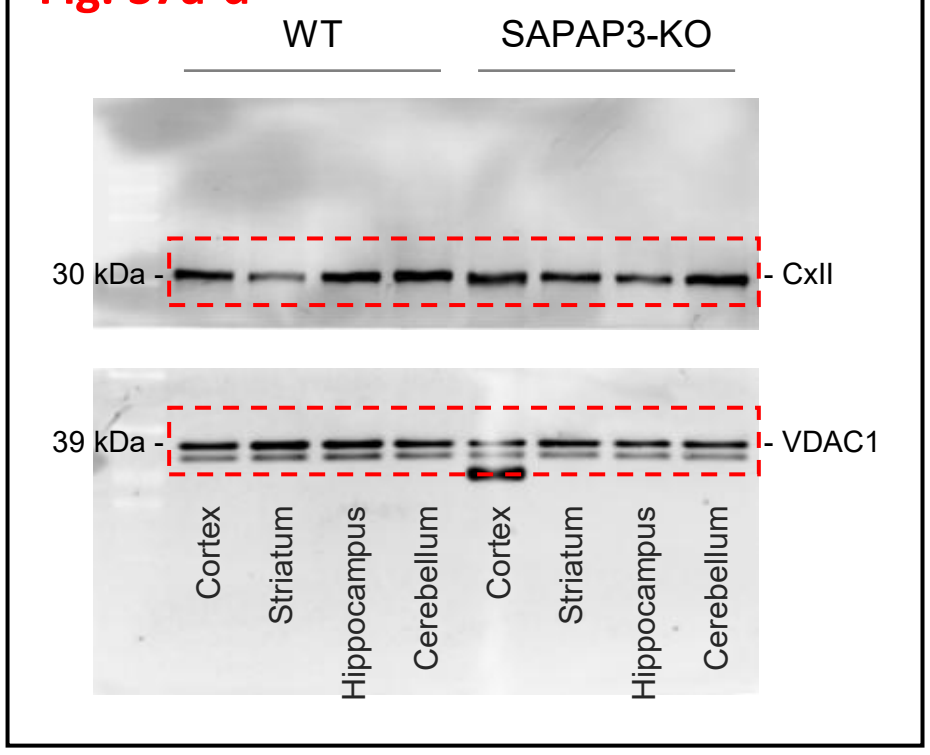

Fig. S7c,d

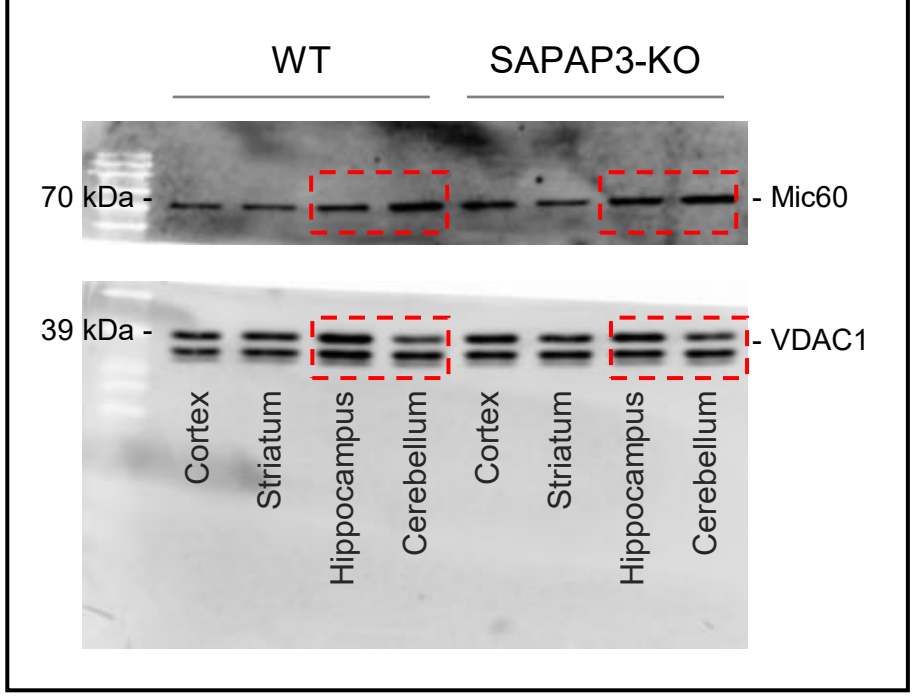

Fig. S7a-d

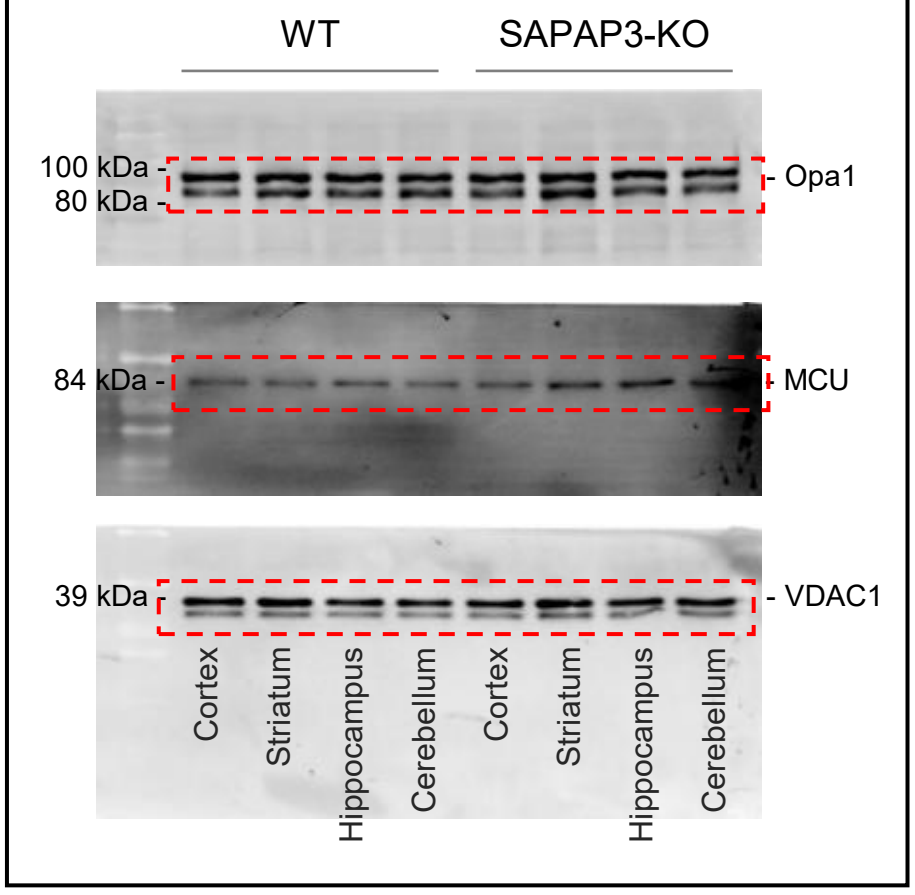

Fig. S8

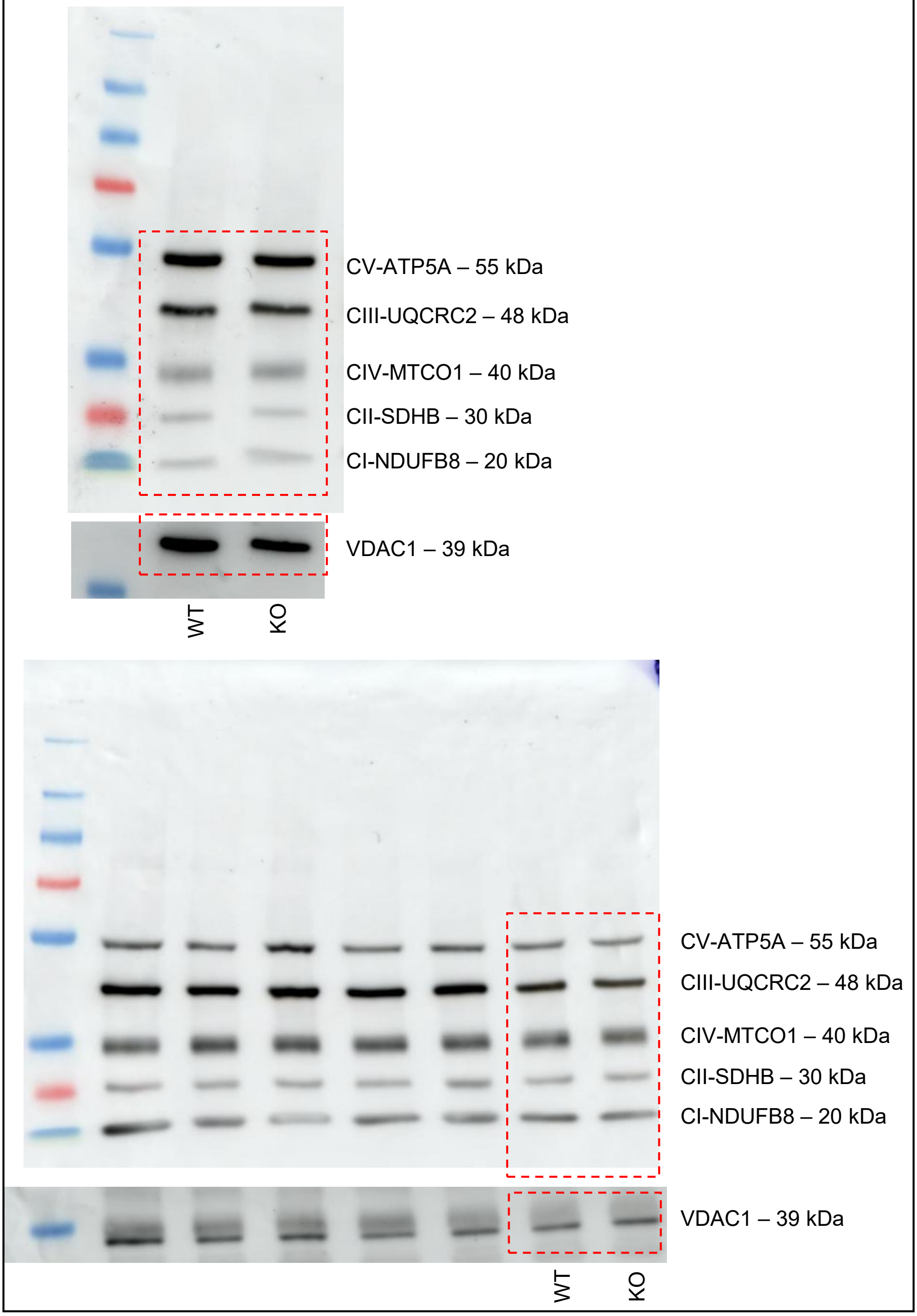
CV-ATP5A – 55 kDa
CIII-UQCRC2 – 48 kDa
CIV-MTCO1 – 40 kDa
CII-SDHB – 30 kDa
CI-NDUFB8 – 20 kDa
VDAC1 – 39 kDa
WT
KO
CV-ATP5A – 55 kDa
CIII-UQCRC2 – 48 kDa
CIV-MTCO1 – 40 kDa
CII-SDHB – 30 kDa
CI-NDUFB8 – 20 kDa
VDAC1 – 39 kDa
WT
KO

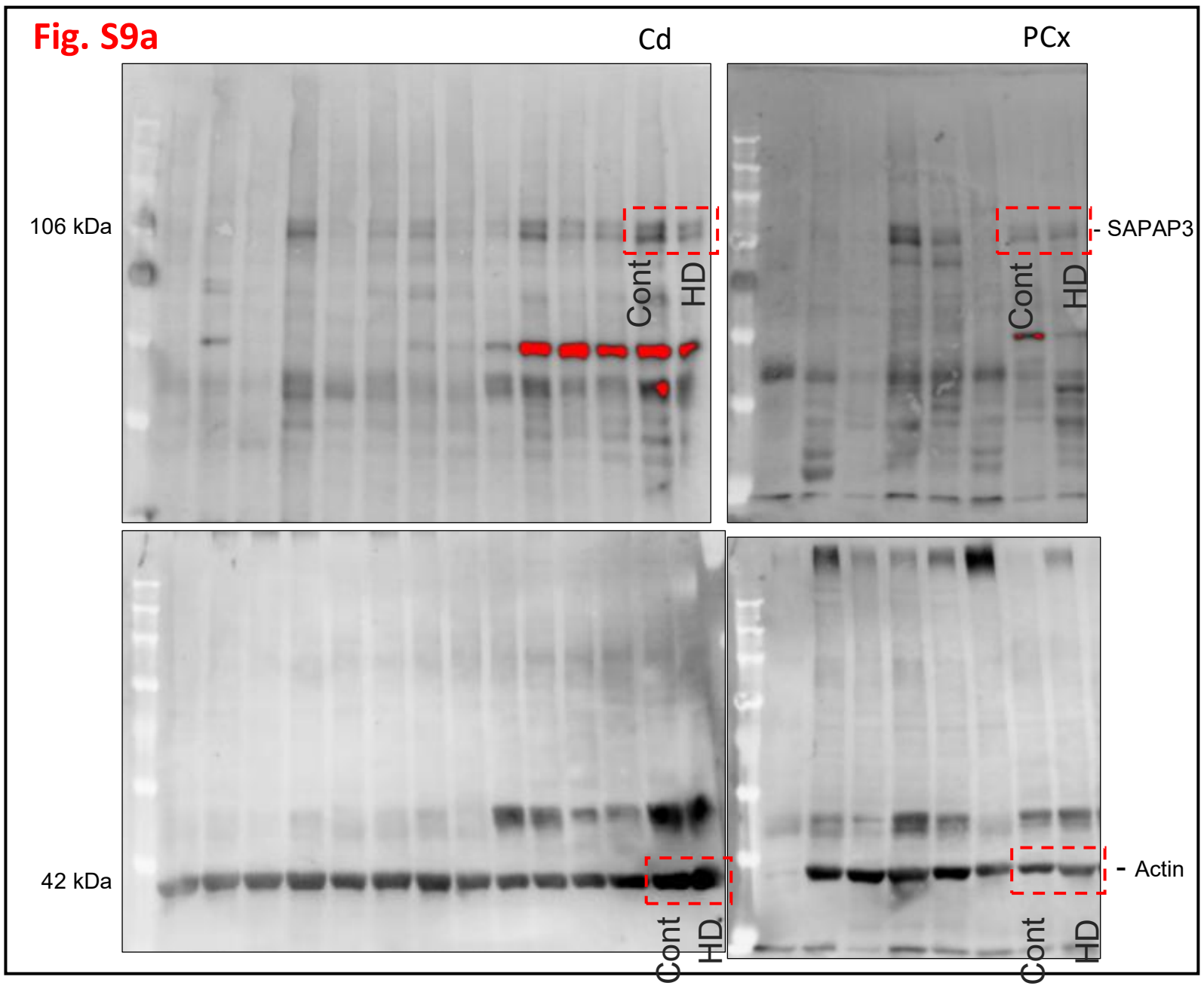
Fig. S9a
Cd
PCx
106 kDa
SAPAP3
Cont
HD
Cont
HD
42 kDa
Actin
Cont
HD
Cont
HD

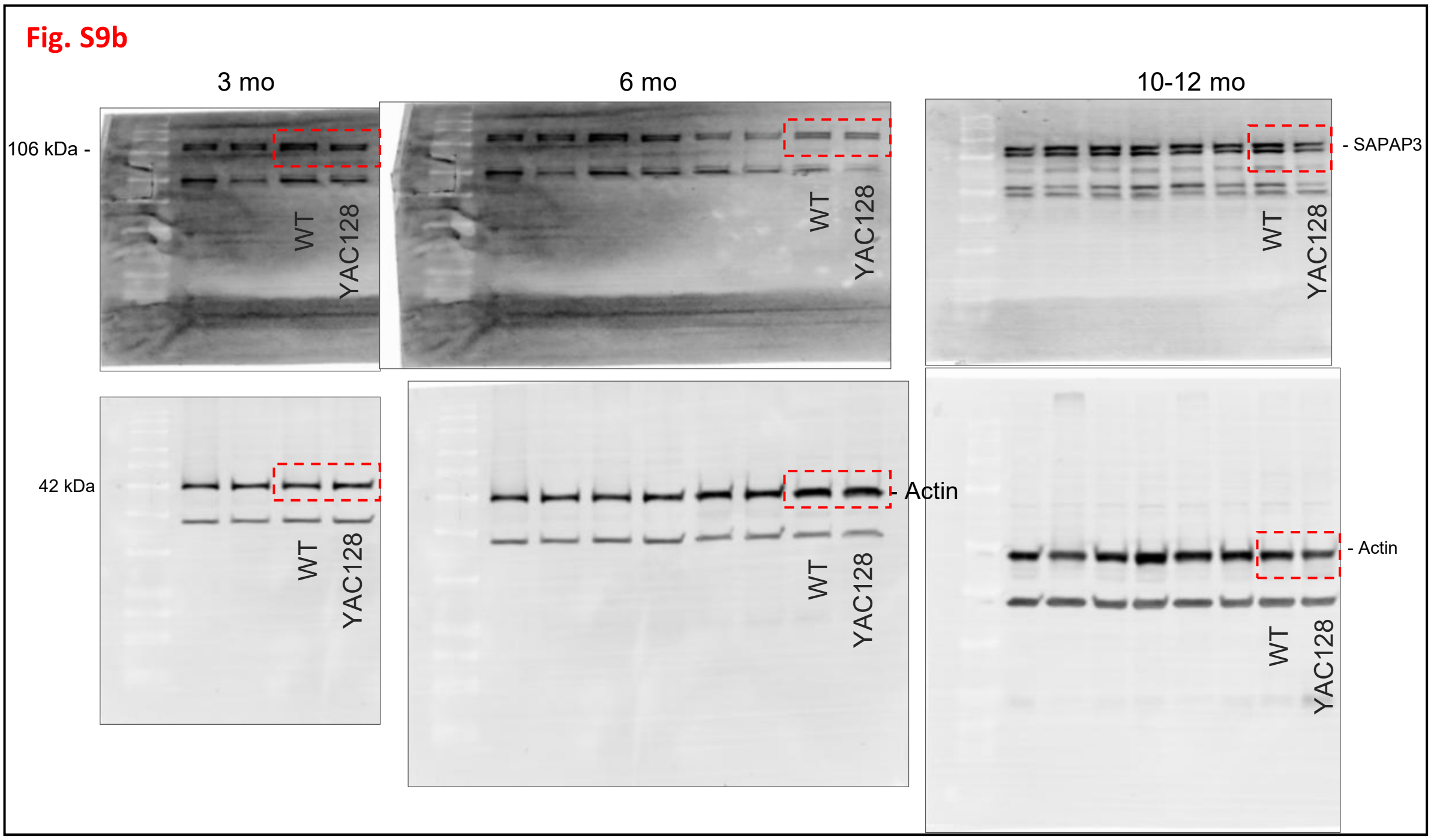
Fig. S9b
3 mo
6 mo
10-12 mo
106 kDa -
- SAPAP3
WT
YAC128
WT
YAC128
WT
YAC128
42 kDa
- Actin
- Actin
WT
YAC128
WT
YAC128
WT
YAC128

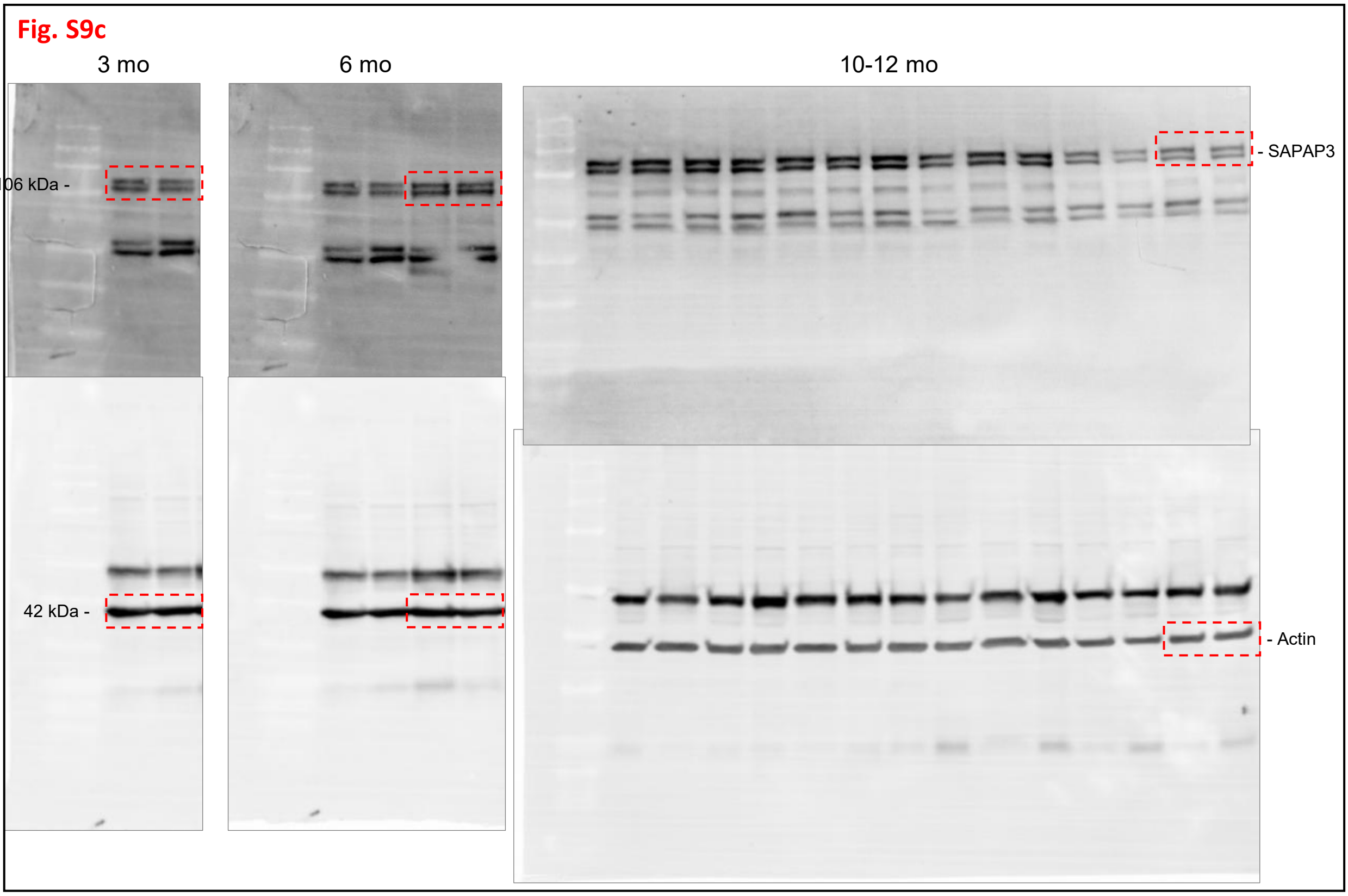
Fig. S9c
3 mo
6 mo
10-12 mo
106 kDa -
42 kDa -
- SAPAP3
- Actin

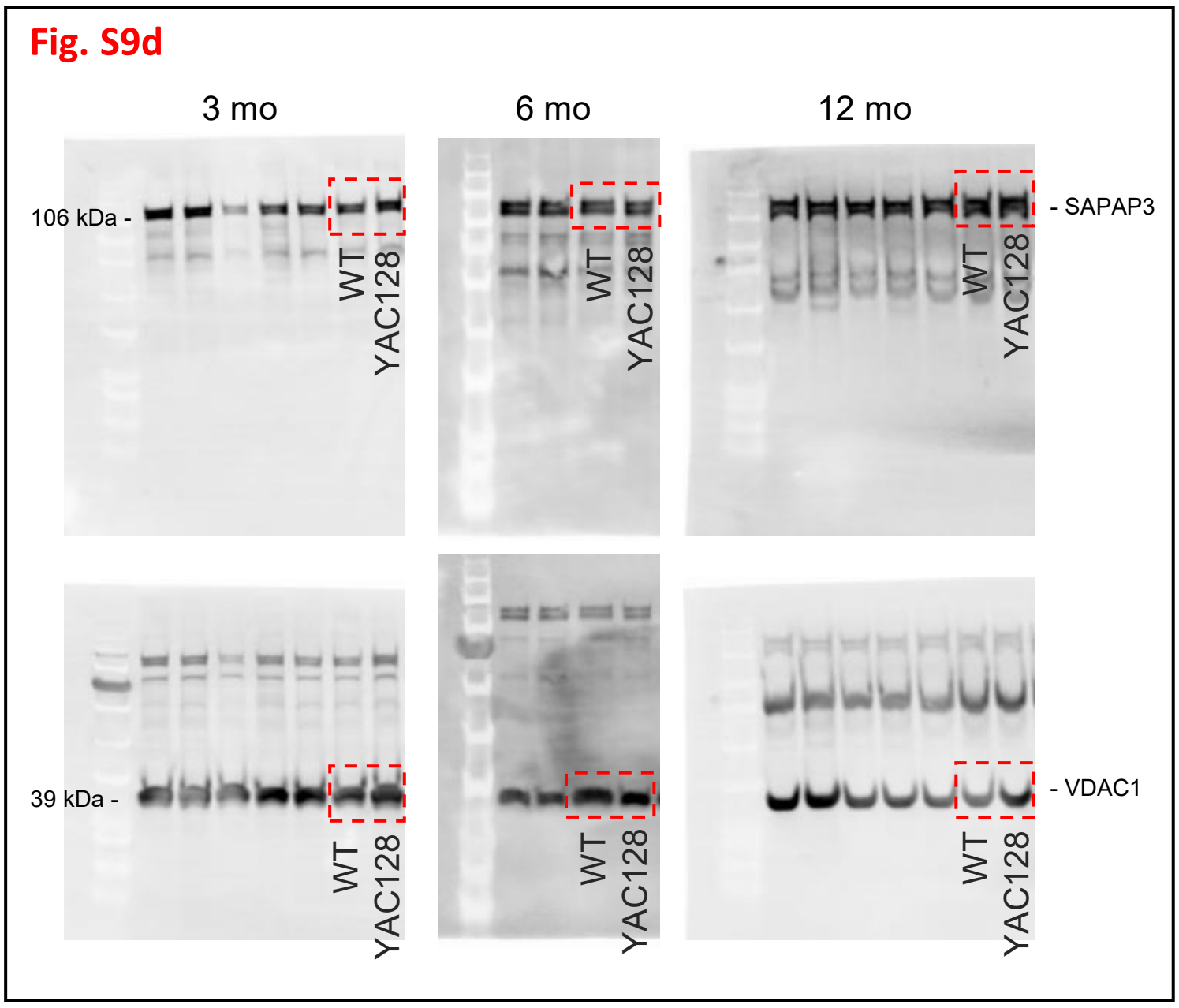
Fig. S9d
3 mo
6 mo
12 mo
106 kDa -
39 kDa -
- SAPAP3
- VDAC1
WT
YAC128
